# Diagnostic Accuracy of Self‐Reported Questionnaires for Detecting Periodontitis Across Multiple Cultures and Geographic Locations: A Systematic Review and Meta‐Analysis

**DOI:** 10.1111/jcpe.70002

**Published:** 2025-08-08

**Authors:** Mengning Bi, Yu Xie, Xiaoyu Yu, Hairui Li, Yuan Li, Maurizio S. Tonetti

**Affiliations:** ^1^ Shanghai PerioImplant Innovation Center, Institute for Oral, Craniofacial and Sensory Research, Ninth People's Hospital Shanghai Jiao Tong University School of Medicine Shanghai China; ^2^ National Clinical Research Center of Oral Diseases and National Center of Stomatology, College of Stomatology Shanghai Jiao Tong University School of Medicine Shanghai China; ^3^ Department of Oral Implantology Ninth People's Hospital Shanghai China; ^4^ European Research Group on Periodontology (ERGOPerio) Genoa Italy

**Keywords:** diagnostic trials, meta‐analysis, periodontitis/diagnosis, screening, self‐reported questionnaires, systematic review

## Abstract

**Objective:**

To evaluate self‐reported questionnaires' intrinsic validity and diagnostic accuracy for detecting periodontitis across diverse cultural and geographic contexts.

**Materials and Methods:**

Electronic searches were conducted in PubMed, Embase and the Cochrane Library until 4 March 2025. Studies included those using the Centers for Disease Control and Prevention and the American Academy of Periodontology (CDC/AAP) criteria or the 2017 World Workshop classifications as reference standards and reporting the diagnostic accuracy of self‐reported questionnaire items. The validity of the eight‐item questionnaire developed by the CDC/AAP was assessed by analysing the proportion of answers, and bias was evaluated using the QUADAS‐2 tool. Owing to the limited number of studies using the 2017 World Workshop classifications, the quantitative synthesis was restricted to those using the CDC/AAP criteria. Sensitivity, specificity, diagnostic odds ratio (DOR) and 95% confidence intervals (CIs) were calculated for total periodontitis (including mild, moderate and severe cases), moderate‐to‐severe periodontitis and severe periodontitis.

**Results:**

Totally, 23 studies (from 22 articles) were included in the systematic review, with 16 studies (from 15 articles) eligible for meta‐analysis. The ‘Don't know/Refused’ option across the eight items of the CDC/AAP questionnaire ranged from 0% to 46.1%, indicating variable comprehension of certain items. Pooled sensitivity and specificity across the questionnaire items ranged from 17% to 82% and from 23% to 97%, respectively. DOR varied from 0.75 (95% CI: 0.54–1.03) to 8.01 (95% CI: 2.33–27.45). Significant heterogeneity was observed for most questions.

**Conclusions:**

Self‐reported questionnaires show potential for monitoring periodontitis but face issues with sensitivity and cross‐cultural validity. Future research should focus on culturally adaptive designs and standardised validation protocols to enhance diagnostic accuracy and improve their effectiveness in global periodontal health screening.

## 1 Introduction

Periodontitis is a significant cause of tooth loss and is associated with an increased risk of systemic diseases, significantly impacting oral and general health (Papapanou et al. 2018; Tonetti et al. 2007). Recognised as one of the foremost global public health challenges, it has significant socio‐economic consequences (Tonetti et al. 2017; Vos et al. 2015).

Detecting periodontitis relies on clinical and radiographic examinations and patient history (Eke et al. 2012; Tonetti et al. 2018). While these clinical assessments are highly accurate, they are time‐consuming and resource‐intensive and require skilled professionals, multiple manual recordings and specialised dental equipment (Kingman and Albandar 2002). These limitations hinder large‐scale epidemiological surveillance and periodontitis screening in non‐clinical settings (Eke and Dye 2009). Therefore, there is a pressing need to develop faster, user‐friendly and cost‐effective detection methods to meet these demands and enhance the early diagnosis of periodontitis, enabling early intervention and cost containment.

Over the past few decades, self‐reported questionnaires have emerged as a promising non‐invasive method for screening periodontitis. Among these, the eight‐item questionnaire developed by the Centers for Disease Control and Prevention and the American Academy of Periodontology (CDC/AAP) (Eke and Genco 2007) is the most widely used tool (Montero et al. 2020). Beyond the original English version, the CDC/AAP questionnaire has been translated into several other languages, including French, Chinese, Japanese and Spanish, among others, and has been validated in diverse cultural contexts (Carra et al. 2018; Deng et al. 2021; Iwasaki et al. 2021; Montero et al. 2020). The validity of various self‐reported questionnaires, including the CDC/AAP instrument, has been established through their acceptable sensitivity and specificity (Bond et al. 2024; Carra et al. 2018; Cyrino et al. 2011; Deng et al. 2021; Kapellas et al. 2021). Furthermore, diagnostic models that combine self‐reported questionnaires with demographic characteristics and periodontitis risk factors have also shown a certain degree of diagnostic accuracy (Carra et al. 2018; Deng et al. 2021; Eke and Dye 2009; Enevold et al. 2024). These questionnaires provide a rapid and cost‐effective method for large‐scale periodontitis screening and surveillance. They are also helpful in non‐dental clinical settings, enabling preliminary diagnosis in high‐risk patients (e.g., diabetes, hypertension) to improve early detection and treatment.

Despite extensive research on self‐reported questionnaires for periodontitis, persistent challenges hinder their optimisation and standardisation. First, regarding questionnaire design and item selection, although the CDC/AAP questionnaire is widely adopted, many studies have incorporated additional self‐designed questions. However, few studies have focused on—and even fewer have systematically assessed—how respondents comprehend these items, as often reflected in the distribution of answers—and evaluated the diagnostic performance of individual items. This complicates identifying the most effective questions for accurate detection.

Second, although most validation studies involve English‐speaking populations, growing research in diverse cultural settings highlights how geographic and cultural differences affect individuals' perception and symptom‐reporting. Cross‐cultural validation is thus essential.

Finally, substantial heterogeneity exists in the reference standards used to validate self‐reported questionnaires (Herrera et al. 2025). Some studies compare questionnaire answers with clinical parameters such as bleeding on probing (BOP) or probing pocket depth (PPD) (Birkeholm Jensen and Haubek 2022; Gilbert and Nuttall 1999; Midwood et al. 2019), while others rely on the Community Periodontal Index of Treatment Needs (CPITN) (Chatzopoulos et al. 2016; Lai et al. 2015; Nijland et al. 2021), CDC/AAP criteria (Eke et al. 2013; Genco et al. 2007; Heaton et al. 2017) or the 2017 World Workshop classifications (Deng et al. 2021; Enevold et al. 2024; Sim et al. 2022). In addition, there are variations in examination protocols—such as full‐mouth (Saka‐Herrán et al. 2020; Taylor and Borgnakke 2007) versus half‐mouth (Enevold et al. 2024; Zhan et al. 2014) assessments—or reliance solely on radiographic evaluation (Callhoff et al. 2019; Dietrich et al. 2005). This methodological inconsistency undermines the comparability of findings across studies and limits the reliability of pooled analyses (Herrera et al. 2025).

Therefore, this systematic review and meta‐analysis aimed to evaluate the existing literature to (i) comprehensively analyse the geographic distribution of studies on self‐reported questionnaires of periodontitis; (ii) assess the distribution of answers to individual questionnaire items to evaluate their intrinsic validity; and (iii) summarise the diagnostic accuracy of individual items for detecting total, moderate‐to‐severe and severe periodontitis.

## 2 Materials and Methods

### 2.1 Protocol Development and Focused Question

The review protocol was registered in the International Prospective Register of Systematic Reviews (PROSPERO) under CRD420250651850. This study was conducted following the *Cochrane Handbook for Systematic Reviews of Diagnostic Test Accuracy* v2.0 (Bossuyt et al. 2023) and is reported following the Preferred Reporting Items for Systematic Reviews and Meta‐Analyses of Diagnostic Test Accuracy Studies (PRISMA‐DTA) guidelines (McInnes et al. 2018). The focused research question was, ‘In the general population, how does the diagnostic performance of individual questions in a self‐reported questionnaire for periodontitis compare with that of a full‐mouth periodontal examination?’

### 2.2 Criteria for Considering Studies

#### 2.2.1 Inclusion Criteria

- *Participants*: General population that included both individuals with and without periodontitis.
- *Target and control conditions*: Clinical diagnoses of periodontitis based on either the CDC/AAP criteria (Eke et al. 2012) or the 2017 classification (Tonetti et al. 2018). The target conditions were classified according to the severity of periodontitis, including total periodontitis (including mild, moderate and severe cases), moderate‐to‐severe periodontitis and severe periodontitis. The corresponding control conditions were defined as (i) periodontal health and gingivitis, (ii) periodontal health, gingivitis and mild periodontitis, and (iii) periodontal health, gingivitis and mild/moderate periodontitis, respectively.
- *Reference standard*: Standard for diagnosing periodontitis based on full‐mouth periodontal examinations, according to either the CDC/AAP criteria or the 2017 classification.
- *Index test (self‐reported questionnaire items)*: The binary results of individual items from the self‐reported periodontitis questionnaire.
- *Outcome*: Diagnostic accuracy measures, including sensitivity, specificity and diagnostic odds ratio (DOR).
- *Study design*: Cross‐sectional studies that reported diagnostic accuracy results for individuals with clinically diagnosed periodontitis.

#### 2.2.2 Exclusion Criteria

- Non‐primary research (theses, dissertations, reviews, letters, opinion pieces, book chapters, conference abstracts).
- Studies lacking a clear periodontitis definition or using criteria other than CDC/AAP or the 2017 World Workshop classifications. Studies employing reference standards without full‐mouth probing.
- Studies that did not report diagnostic performance for individual questions in detecting periodontitis.
- Studies specifically recruiting participants with particular medical conditions that may influence periodontal status or self‐report accuracy, such as rheumatoid arthritis.

### 2.3 Search Methods for the Identification and Selection of Studies

#### 2.3.1 Information Sources and Search Strategy

On 4 March 2025, an electronic search was performed in PubMed, Embase, and Cochrane CENTRAL. The search strategies and results are listed in Table S1.

#### 2.3.2 Selection of Studies

After removing duplicates, two independent reviewers (M.N.B. and Y.X.) screened titles and abstracts based on the eligibility criteria, then assessed the full texts of the included studies. Disagreements were resolved through discussion or, if needed, by a third reviewer (Y.L.). Inter‐reviewer reliability was calculated.

### 2.4 Data Extraction Methods

We extracted essential information about each study, including (i) basic characteristics, (ii) reference standards (diagnostic criteria and clinical examination details), (iii) the context and answer distribution of the self‐reported questionnaire and (iv) the confusion matrix, detailing true positives (TP), false positives (FP), true negatives (TN) and false negatives (FN) for each question.

### 2.5 Quality Assessment of Individual Studies

Quality assessment of the included studies was performed by M.N.B. and Y.X. using the revised version of the Quality Assessment of Diagnostic Accuracy Studies‐2 (QUADAS‐2) tool (Whiting et al. 2011). Each study was assessed across four key domains: patient selection, which examines how study participants were identified and enrolled; index test, which evaluates whether the questionnaire items were applied and interpreted without knowledge of the reference standard; reference standard, which considers whether the clinical diagnosis was applied consistently and independently of the questionnaire results; and flow and timing, which addresses the interval between administration of the questionnaire and the reference standard, as well as whether all participants received both assessments and were included in the analysis.

### 2.6 Quantitative Data Analysis

Quantitative analyses were performed using R (v 4.3.1; The R Foundation for Statistical Computing Platform).

- *Detection of between‐study heterogeneity*: Cochran's *Q* test assessed the heterogeneity between studies. The Higgins *I* ^2^ test determined the proportion of variability attributed to between‐study heterogeneity.
- *Data synthesis*: To address zero‐cell counts in 2 × 2 tables, which render the DOR undefined, a continuity correction of 0.5 was added to all cells. This approach allows for the calculation of an approximate DOR, facilitating inclusion of all studies in the meta‐analysis (Glas et al. 2003; Haldane 1956; Littenberg and Moses 1993). To estimate and visualise the hierarchical summary receiver operating characteristic (HSROC) curves, we used a Bayesian hierarchical model implemented via the *meta4diag* R package, fitting a Bayesian bivariate random‐effects model using the integrated nested Laplace approximation (INLA) for inference. HSROC curves were generated based on posterior means, accompanied by 95% credible and prediction regions (Guo and Riebler 2018; Rue et al. 2009).
- *Influence analysis*: A Baujat plot and leave‐one‐out analysis were made to identify any studies or effect sizes that significantly impacted the meta‐analysis results or between‐study heterogeneity. These analyses used a random‐effects model with inverse variance weighting for pooling.
- *Publication bias*: Deeks's funnel plot asymmetry test was used to evaluate publication bias by performing a weighted regression of the DOR against the inverse square root of the effective sample size (Bossuyt et al. 2023; Deeks et al. 2005).

## 3 Results

### 3.1 Study Selection

**FIGURE 1:**
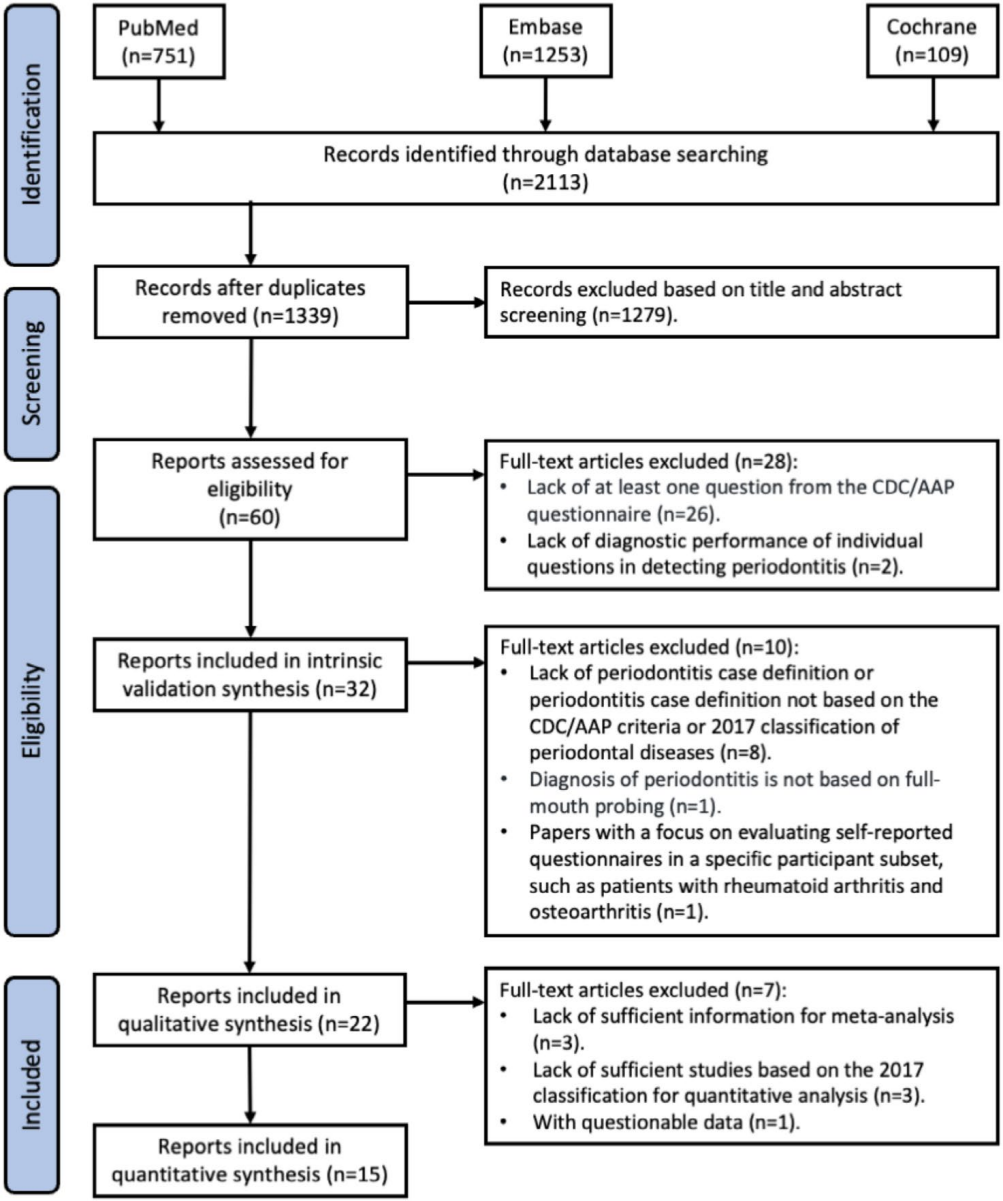
Flow diagram of study selection. PRISMA diagram of the included studies.

A total of 2113 articles were identified from PubMed, the Cochrane Library and Embase. After removing duplicates, 1339 titles and abstracts were screened, and 60 full texts were assessed. Of these, 32 articles were included in the validation synthesis and 22 (23 studies) in the qualitative synthesis. Authors were contacted for missing data; studies without adequate data were excluded. As shown in Figure 1, 15 articles (16 studies) were ultimately included in the quantitative synthesis.

### 3.2 Intrinsic Validity of the CDC/AAP Questionnaire

**FIGURE 2:**
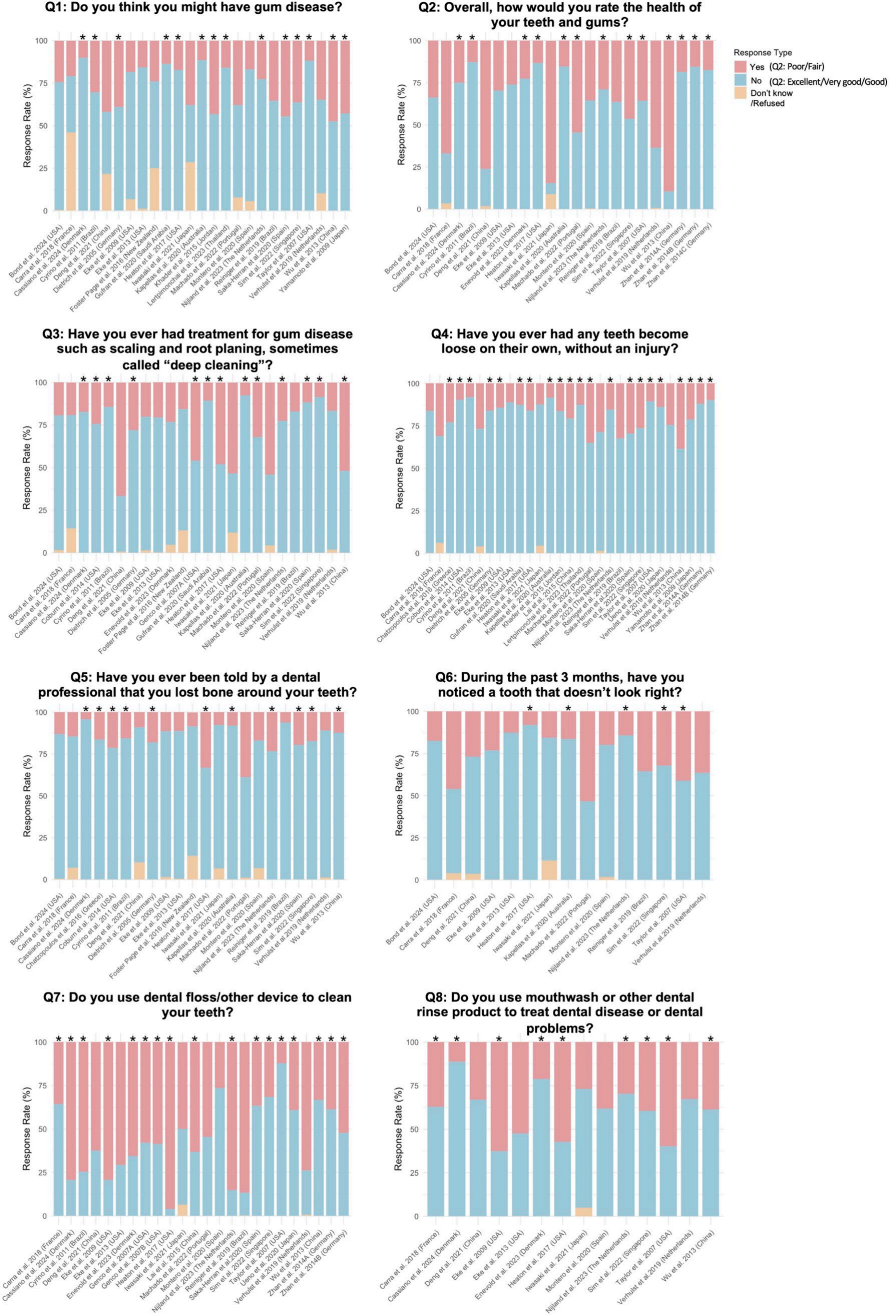
Answer distribution for items in the CDC/AAP questionnaire. The multiple panels illustrate the proportion of answers to individual items in the CDC/AAP questionnaire reported in the included studies. Red shows the rate of ‘Yes’ answers, blue the rate of ‘No’ answers and yellow the rate of ‘Don't Know/Refused’ questions. Asterisks indicate that the ‘Don't know/Refused’ option was not provided or reported.

The proportion of answers for each item in the CDC/AAP questionnaire (specific content of the eight questions shown in Table S2) was first analysed to evaluate the validity of the questionnaire items (Figure 2). A high proportion of ‘Don't know/Refused’ answers indicates that the question may be difficult for the public to understand, impacting its diagnostic accuracy. More than half of the studies did not include or report the ‘Don't know/Refused’ option. Among all questions, Q1 (perceived gum disease) had the highest proportion of ‘Don't know/Refused’ answers, with some studies reporting nearly 50%, highlighting challenges in public self‐awareness of periodontitis. Q3 (periodontal treatment history) and Q5 (bone loss diagnosis) also elicited a relatively high proportion of uncertain answers, suggesting that participants in many studies struggled with questions related to professional periodontal diagnosis and treatment.

### 3.3 Study Characteristics

The 23 studies included in the qualitative synthesis had sample sizes ranging from 77 to 6966 (Table 1). Regarding the reference standards for periodontal diagnosis, 17 studies used the CDC/AAP criteria (Eke et al. 2012), 3 applied the 2017 classification (Tonetti et al. 2018) and 3 used both diagnostic criteria. Some studies reported detailed staging, while others focused only on moderate‐to‐severe or severe periodontitis, grouping the rest as controls.

**TABLE 1:** Characteristics of the included studies in the qualitative synthesis.

Study	Country	Population	Diagnostic criteria	Category (*n*)	Age, years	Female sex (%)	Current smoking (%)	Diabetes (%)	Cardiovascular disease (%)	High blood pressure (%)
Bond et al. (2024)	U.S.A.	General population, 6966 participants	CDC/AAP criteria	No: 3600 Mild: 135 Moderate: 2477 Severe: 754	48.9 ± 13.5 43.7 ± 11.2 55.8 ± 14.4 56.1 ± 11.5	59 45.2 46.4 29.2	12.4 11.9 22.6 35	11.6 13.3 21.5 19.4	NR	NR
Carra et al. (2018)	France	General population, 232 participants	CDC/AAP criteria	No: 18 Moderate: 105 Severe: 109	46.7 ± 12.68	40.1	29.1	1.7	3.4	11.6
Cassiano et al. (2024)	Denmark	General population, 197 participants	CDC/AAP criteria	No: 129 Total periodontitis: 68 Moderate/severe: 50	NR	73.6 69.1 66	28.7 47.1 58	NR	NR	2.3 7.4 8
Cyrino et al. (2011)	Brazil	Employees of a large corporation in Brazil, 284 participants	CDC/AAP criteria	No/Mild: 202 Moderate: 49 Severe: 33	34.52 ± 0.81 42.45 ± 1.58 47.34 ± 1.63	29.7 22.4 15.2	11.4 24.5 21.2	1.5 6.1 6.1	NR	NR
Deng et al. (2021)	Hong Kong SAR, China	General population, 408 participants	2017 classification of periodontal diseases	H: 66 G: 62 I: 65 II: 65 III: 121 IV: 29	30 ± 11 26 ± 5 28 ± 8 45 ± 15 58 ± 12 57 ± 13	62.1 54.8 49.2 43.1 50.9 41.4	0 1.6 1.5 7.7 14.9 24.1	0 0 0 7.7 8.3 17.2	1.5 0 1.5 9.2 25.6 20.7	NR
Eke and Dye (2009)	U.S.A.	General population, 456 participants	CDC/AAP criteria	Total periodontitis: 102 Moderate: 80 Severe: 22	For all participants: ≥ 65: 28.9, 23.7, 5.3 50–64: 25.8, 19.4, 6.5 35–49: 18.9, 15.3, 3.6	32.4 35.0 22.7	35.3 31.3 50.0	6.9 6.3 9.1	NR	NR
Enevold et al. (2024)	Denmark	General population, 1476 participants	2017 classification of periodontal diseases	All participants: 1476 Stage III/IV: 342	56 62	28.9 22.5	65.4 80.4	NR	NR	NR
Genco et al. (2007)	U.S.A.	Group A: 1578 patients with/without myocardial infarction; Group B: general population, 1438 participants	CDC/AAP criteria	Group A: No/Mild: 297 Moderate: 813 Severe: 468 Group B: No/Mild: 391 Moderate: 603 Severe: 444	For all participants: Group A: 35–39: 3 40–54: 43 55–64: 29 ≥ 65: 25 Group B: 25–29: 10 30–39: 25 40–54: 34 55–64: 12 ≥ 65: 19	Group A: 44 Group B: 53	Group A: 13 Group B: 28	Group A: 10 Group B: 5	NR	NR
Heaton et al. (2017)	U.S.A.	77 American Black women	CDC/AAP criteria	No/Mild: 11 Moderate: 46 Severe: 18	56 59.2 61.1	100	0 6.5 26.7	18.2 28.3 16.7	NR	NR
Iwasaki et al. (2021)	Japan	Workers living in Fukuoka Prefecture, Japan, 949 participants	CDC/AAP criteria	No: 541 Mild: 64 Moderate: 285 Severe: 59	41.1 ± 11.3 41.1 ± 11.8 45.9 ± 12.7 51.7 ± 11.7	79.5 67.2 62.1 54.2	9.6 17.2 18.2 28.8	4.6 3.1 10.9 11.9	NR	NR
Kapellas et al. (2021)	Australia	General population, 3630 participants	CDC/AAP criteria	Moderate/severe: 1174	For all participants: 15–44: 19.0 45–64: 53.6 ≥ 65: 27.4	50.5	20.2	7.2	NR	NR
Lertpimonchai et al. (2023)	Thailand	Employees of the Electricity Generating Authority of Thailand, 1393 participants	CDC/AAP criteria	Severe: 483	61.1 ± 4.4	20.7	21.4	16.2	NR	NR
Machado et al. (2022)	Portugal	General population, 103 participants	2017 classification of periodontal diseases and CDC/AAP criteria	2017 Classification total periodontitis: 63 2017 Classification severe (stage III/IV) periodontitis: 39 CDC/AAP 2012 total periodontitis: 71 CDC/AAP 2012 severe periodontitis: 71	56.3 ± 13.2 59.2 ± 11.5 54.7 ± 13.9 58.3 ± 11.7	49.2 43.6 53.5 44.6	30.2 30.8 29.6 32.1	NR	NR	NR
Montero et al. (2020)	Spain	General population, 231 participants	CDC/AAP criteria	No/Mild: 21 Moderate: 82 Severe: 128	For all participants: < 35: 5.2 35–49: 24.7 50–64: 31.6 ≥ 65: 38.5	46.8	33.3 19.5 22.7	4.8 14.6 23.4	NR	NR
Reiniger et al. (2020)	Brazil	Residents in a rural area of Brazil, 585 participants	2017 classification of periodontal diseases and CDC/AAP criteria	2017 Classification severe periodontitis: 236 CDC/AAP severe periodontitis: 198	54.98 ± 13.02 52.47 ± 13.11	43.2 40.4	21.2 22.2	7.9 5.7	NR	NR
Sim et al. (2022)	Singapore	Adult population, 155 participants	2017 classification of periodontal diseases	Severe: 90	For all participants: < 45: 19.4 45–54: 23.9 55–64: 31.6 ≥ 65: 25.2	35.6	8.9	31.1	NR	NR
Taylor and Borgnakke (2007)	U.S.A	Adult population, 455 participants	CDC/AAP criteria	No/mild: 289 Moderate: 110 Severe: 56	For all participants: 18–29: 23.1 30–39: 28.1 40–54: 28.4 55–64: 9.0 ≥ 65: 11.4	59.5 60.9 39.3	29.4 34.5 32.1	2.4 6.4 12.5	NR	NR
Ueno et al. (2020)	Japan	Adult population, 2404 participants	CDC/AAP criteria	No: 636 Mild: 65 Moderate: 1328 Severe: 375	For all participants: 40–49: 20.2 50–59: 30.1 60–69: 35.4 70–75: 14.3	68.7 67.6 69.1 54.9	8.8 9.2 14.4 18.7	NR	NR	NR
Wu et al. (2013)	Mainland China	Adult population, 114 participants	CDC/AAP criteria	Mild: 58 Moderate/severe: 56 Severe: 28	35 ± 11 54 ± 14 58 ± 13	63.8 55.4 46.4	6.9 26.8 46.4	NR	NR	NR

*Note*: Data are presented as mean ± SD or median with interquartile range (IQR).

Abbreviations: CDC/AAP, the Centers for Disease Control and Prevention and the American Academy of Periodontology; G, gingivitis; H, periodontal health; I, Stage I periodontitis; II, Stage II periodontitis; III, Stage III periodontitis; IV, Stage IV periodontitis; Mild, mild periodontitis; Moderate, moderate periodontitis; No, non‐periodontitis; NR, not reported; Severe, severe periodontitis.

Across 23 studies, 41 self‐reported questions were analysed, including 8 from the CDC/AAP questionnaire and 33 customised questions. These were grouped into five domains: overall self‐perception of oral health, symptom‐related questions, oral health care, information received from dental professionals and dental treatment experience. The full list is in Table S3.

### 3.4 Quality Assessment

The quality assessment results of the studies are summarised in Figure S1. Three studies had a high risk of bias in the patient selection domain, while all studies showed a low risk in the index test domain. Twelve studies had an unclear risk of bias in the reference standard domain because they did not report whether the clinical examiners were blinded to the questionnaire answers. Additionally, two studies showed an unclear risk of bias related to the timing of the clinical reference and telephone interview.

### 3.5 Geographic Distribution of Included Studies

**FIGURE 3:**
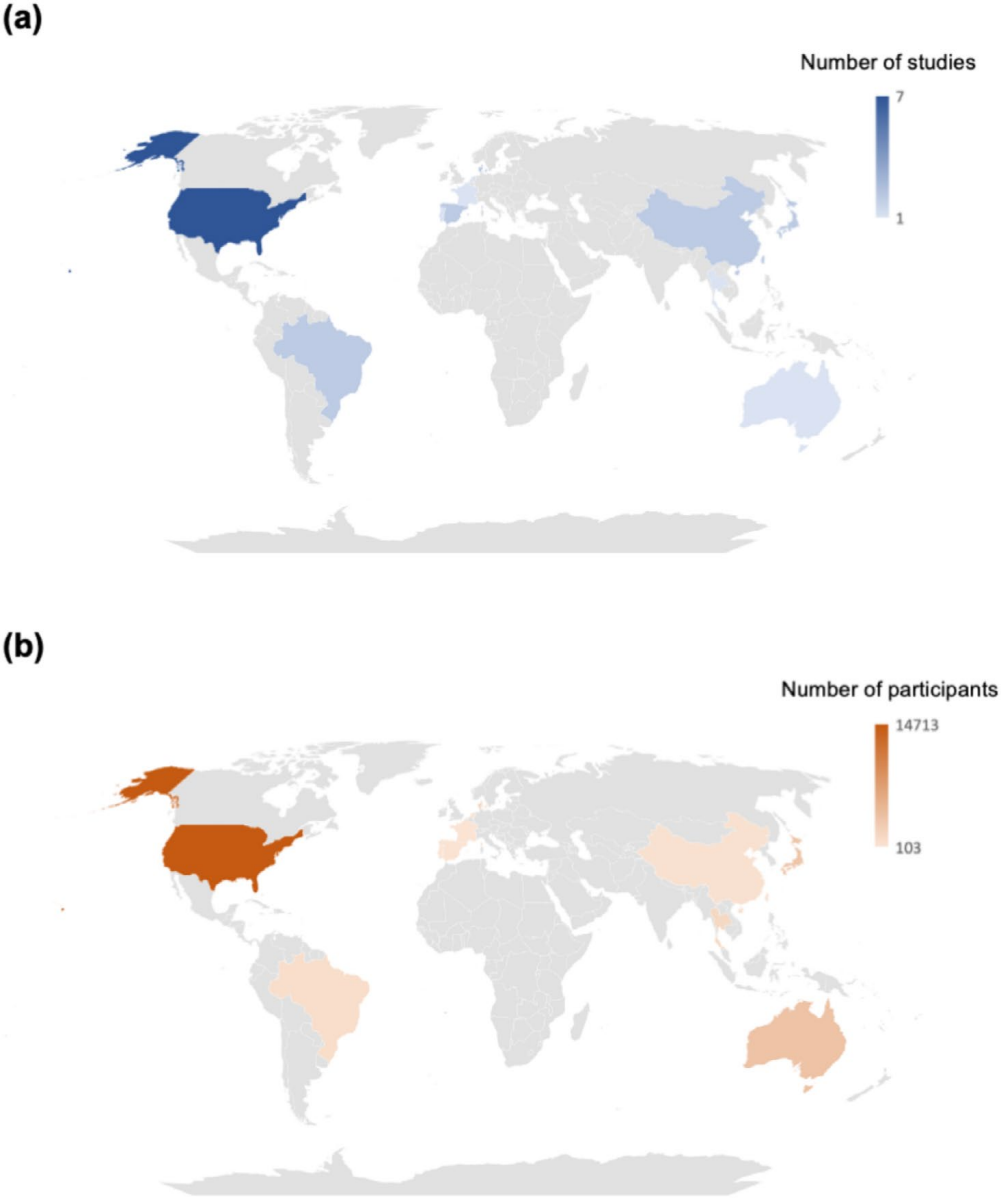
Geographic distribution of studies included in the qualitative synthesis. The world map shows the number of studies (panel a) and the number of participants (panel b) reporting samples from different locations. The colour gradient in the heatmap quantifies the observations.

The studies included in this systematic review encompass a wide geographic distribution and diverse cultural backgrounds (Figure 3). Specifically, the review comprises six studies from Asia (China = 2, Japan = 2, Singapore = 1 and Thailand = 1), seven from Europe (Denmark = 2, France = 1, Netherlands = 1, Portugal = 1 and Spain = 2), nine from the Americas (USA = 7 and Brazil = 2) and one from Oceania (Australia). Most studies and participants were from the United States, Australia and Japan. The high number of participants in these countries is primarily due to the use of large‐scale survey databases, such as the National Health and Nutrition Examination Survey (NHANES) in the United States.

### 3.6 Study Heterogeneity

Owing to the limited use of the 2017 classification (fewer than three studies per stage), the quantitative analysis included only the 16 studies using the CDC/AAP definition. Three diagnostic definitions were applied: Group A considered total periodontitis positive; Group B classified moderate‐to‐severe periodontitis as positive; and Group C defined severe periodontitis as positive. In all three definitions, cases not meeting the positive criteria were considered negative (see Section 2 for details). Diagnostic performance analysis was conducted for individual questions reported in at least three studies within each definition group, making 16 questions eligible for analysis.

Cochran's *Q* values for individual questions and definition categories ranged from 11.59 to 373.34 for sensitivity and from 1.00 to 864.89 for specificity (Table S4). Most Chi‐squared (*χ* ^2^) tests had *p*‐values < 0.05, indicating significant heterogeneity. Higgins's *I* ^2^ ranged from 73.4% to 98.4% (sensitivity) and from 0% to 99.6% (specificity), with most values over 85%, confirming substantial between‐study heterogeneity.

### 3.7 Quantitative Synthesis

Table 2 summarises the pooled sensitivity, specificity and DOR for detecting total, moderate‐to‐severe and severe periodontitis. Questions Q1 (perceived gum disease), Q3 (periodontal treatment history), Q4 (loose teeth history), Q5 (bone loss diagnosis), Q6 (abnormal tooth appearance), Q9 (bleeding gums), Q11 (tooth loss due to mobility), Q12 (breath/taste satisfaction), Q13 (periodontal surgery history), Q14 (tooth brushing frequency), Q15 (dental checkup frequency) and Q16 (sore gums) had low sensitivity but high specificity, with Q4 and Q5 consistently showing strong specificity across all severity levels. Conversely, Q2 (oral health rating), Q8 (mouthwash use) and Q10 (tooth loss) exhibited moderate sensitivity but low specificity. Overall, the questions performed better in specificity than sensitivity.

**TABLE 2:** Pooled sensitivity, specificity and diagnostic odds ratio (DOR) for each question in the self‐reported questionnaire in detecting total, moderate‐to‐severe and severe periodontitis.

Questions	Clinical diagnose	Number of included studies	Pooled sensitivity (%) (95% CI)	Pooled specificity (%) (95% CI)	Pooled DOR (95% CI)
Q1: Do you think you might have gum disease?^a^ (‘yes’ indicates a positive result)	Total periodontitis	4	48 (33, 62)	92 (74, 99)	8.01 (2.33, 27.45)
	Moderate‐to‐severe periodontitis	10	37 (27, 47)	89 (80, 96)	3.60 (2.16, 5.99)
	Severe periodontitis	12	47 (36, 59)	79 (72, 85)	3.20 (2.32, 4.43)
Q2: Overall, how would you rate the health of your teeth and gums?^a^ (‘fair/poor’ indicates a positive result)	Total periodontitis	4	73 (50, 88)	52 (22, 81)	2.82 (1.59, 5.01)
	Moderate‐to‐severe periodontitis	10	56 (39, 73)	69 (48, 85)	2.87 (2.08, 3.94)
	Severe periodontitis	11	66 (49, 81)	59 (39, 76)	2.84 (2.26, 3.57)
Q3: Have you ever had treatment for gum disease such as scaling and root planing, sometimes called ‘deep cleaning’?^a^ (‘yes’ indicates a positive result)	Total periodontitis	4	41 (26, 57)	84 (57, 97)	3.27 (0.97, 11.08)
	Moderate‐to‐severe periodontitis	10	40 (32, 49)	75 (63, 84)	1.94 (1.28, 2.95)
	Severe periodontitis	11	51 (42, 59)	71 (59, 80)	2.36 (1.67, 3.32)
Q4: Have you ever had any teeth become loose on their own, without an injury?^a^ (‘yes’ indicates a positive result)	Total periodontitis	4	27 (15, 42)	90 (85, 93)	2.35 (1.38, 4.03)
	Moderate‐to‐severe periodontitis	10	26 (19, 35)	92 (90, 93)	3.41 (2.23, 5.22)
	Severe periodontitis	13	43 (33, 54)	88 (85, 91)	5.46 (3.73, 8.01)
Q5: Have you ever been told by a dental professional that you lost bone around your teeth?^a^ (‘yes’ indicates a positive result)	Total periodontitis	4	17 (5, 40)	97 (94, 100)	3.97 (0.77, 20.41)
	Moderate‐to‐severe periodontitis	9	19 (15, 23)	92 (90, 95)	2.49 (1.75, 3.53)
	Severe periodontitis	10	32 (24, 42)	89 (85, 92)	3.73 (2.22, 6.28)
Q6: During the past 3 months, have you noticed a tooth that doesn't look right?^a^ (‘yes’ indicates a positive result)	Total periodontitis	3	52 (17, 87)	80 (61, 90)	3.70 (0.64, 21.42)
	Moderate‐to‐severe periodontitis	7	30 (21, 40)	84 (76, 91)	2.23 (1.90, 2.63)
	Severe periodontitis	9	44 (29, 60)	78 (71, 85)	2.62 (1.88, 3.65)
Q7: Do you use dental floss/other device to clean your teeth?^a^ (‘never’ indicates a positive result)	Total periodontitis	5	46 (29, 63)	56 (46, 65)	1.12 (0.79, 1.61)
	Moderate‐to‐severe periodontitis	12	47 (33, 61)	59 (43, 75)	1.28 (0.96, 1.71)
	Severe periodontitis	13	50 (39, 62)	54 (38, 69)	1.16 (0.81, 1.67)
Q8: Do you use mouthwash or other dental rinse products to treat dental disease or dental problems?^a^ (‘never’ indicates a positive result)	Total periodontitis	3	74 (60, 86)	23 (12, 37)	0.92 (0.71, 1.21)
	Moderate‐to‐severe periodontitis	8	58 (45, 69)	39 (27, 51)	0.85 (0.65, 1.12)
	Severe periodontitis	7	49 (36, 61)	44 (35, 53)	0.75 (0.54, 1.03)
Q9: Do you have bleeding gums? (‘yes’ indicates a positive result)	Total periodontitis	4	37 (24, 52)	81 (57, 98)	1.58 (1.34, 1.86)
	Moderate‐to‐severe periodontitis	7	38 (28, 48)	71 (63, 78)	1.49 (1.33, 1.68)
	Severe periodontitis	8	42 (30, 55)	73 (62, 82)	1.93 (1.54, 2.40)
Q10: Do you have tooth loss? (‘yes’ indicates a positive result)	Moderate‐to‐severe periodontitis	4	78 (69, 85)	47 (31, 63)	2.98 (1.72, 5.15)
	Severe periodontitis	3	82 (72, 90)	33 (20, 48)	2.16 (1.02, 4.55)
Q11: Do you have tooth loss because of mobility? (‘yes’ indicates a positive result)	Severe periodontitis	4	49 (23, 77)	89 (83, 95)	6.87 (3.76, 12.53)
Q12: Are you satisfied with your breath/taste? (‘no’ indicates a positive result)	Moderate‐to‐severe periodontitis	4	33 (17, 52)	64 (34, 87)	0.95 (0.56, 1.60)
	Severe periodontitis	4	41 (24, 60)	58 (29, 84)	1.07 (0.54, 2.13)
Q13: Have you ever had periodontal surgery? (‘yes’ indicates a positive result)	Moderate‐to‐severe periodontitis	4	21 (16, 27)	91 (85, 96)	2.93 (1.95, 4.39)
	Severe periodontitis	5	26 (20, 34)	87 (82, 91)	2.61 (2.05, 3.32)
Q14: Frequency of tooth brushing (‘At most once a day’ indicates a positive result)	Severe periodontitis	3	22 (7, 43)	88 (69, 98)	1.70 (1.00, 2.89)
Q15: Frequency of Dental checkups (‘Less than once a year’ indicates a positive result)	Moderate‐to‐severe periodontitis	3	40 (28, 52)	70 (61, 78)	1.56 (1.27, 1.91)
	Severe periodontitis	3	37 (22, 52)	66 (54, 76)	1.20 (0.96, 1.50)
Q16: Do you have sore gums? (‘yes’ indicates a positive result)	Moderate‐to‐severe periodontitis	4	44 (34, 53)	70 (58, 80)	1.57 (1.33, 1.86)
	Severe periodontitis	6	41 (28, 54)	75 (64, 84)	2.09 (1.80, 2.42)

*Note*: Total periodontitis is defined as the presence of mild, moderate or severe periodontitis.

^a^ Q1–Q8 are derived from the CDC/AAP questionnaire.

For total periodontitis, the most sensitive question was about dental rinse products (sensitivity = 74%, 95% CI: 60%–86%). Q10 (tooth loss) had the highest sensitivity for both moderate‐to‐severe (78%, 95% CI: 69%–85%) and severe periodontitis (82%, 95% CI: 72%–90%). Q5 (bone loss diagnosis) showed the highest specificity across all severity levels: 97% for total periodontitis, 92% for moderate‐to‐severe periodontitis and 89% for severe periodontitis.

DOR analysis showed that the 95% confidence intervals for Q1–Q2, Q4, Q9–Q11, Q13 and Q16 were all above 1, indicating their effectiveness in distinguishing between individuals with periodontitis and those without. Q1 had the highest diagnostic performance for overall periodontitis, while Q11 excelled in diagnosing severe cases. Forest plots detailing sensitivity, specificity and DOR for each question are shown in Figures S2–S40.

**FIGURE 4:**
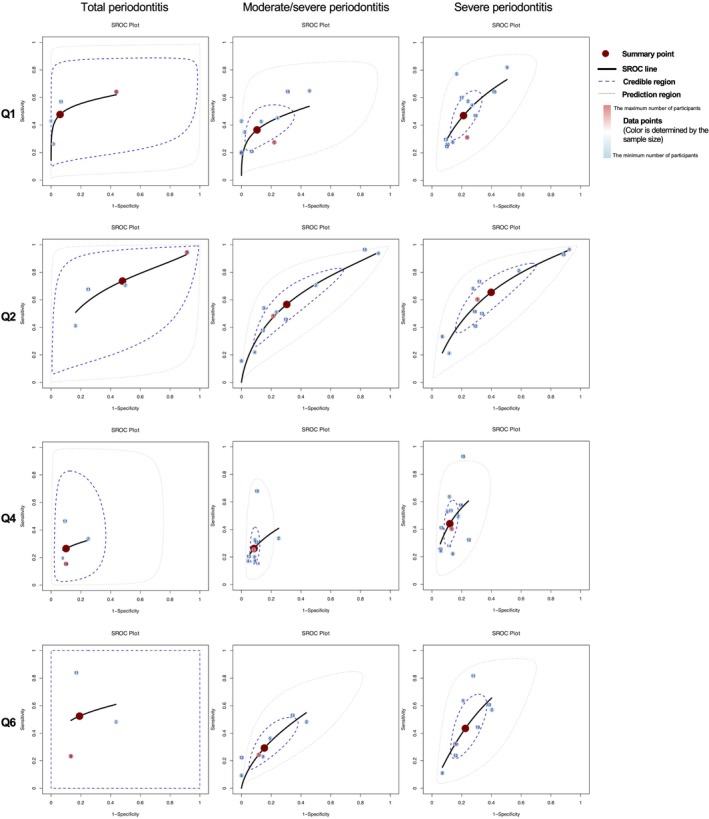
Hierarchical summary receiver operating characteristic (HSROC) curves for Q1, Q2, Q4 and Q6 in the CDC/AAP questionnaire. The question numbers correspond to those in Figure 2. The left column indicates total periodontitis (including mild, moderate, and severe cases), the middle column moderate/severe periodontitis and the right column severe periodontitis. Each point represents a study. The colour of the study points ranges from blue to red, indicating that the sample size varies from small to large. The numbers on the points correspond to the following study references: (1) Bond et al. (2024); (2) Carra et al. (2018); (3) Cassiano et al. (2024); (4), Cyrino et al. (2011); (5) Eke and Dye (2009); (6) Genco et al. (2007); (7) Genco et al. (2007); (8) Heaton et al. (2017); (9) Iwasaki et al. (2021); (10) Lertpimonchai et al. (2023); (11) Machado et al. (2022); (12) Montero et al. (2020); (13) Taylor and Borgnakke (2007); (14) Ueno et al. (2020); (15) Wu et al. (2013); (16) Reiniger et al. (2020).

**FIGURE 5:**
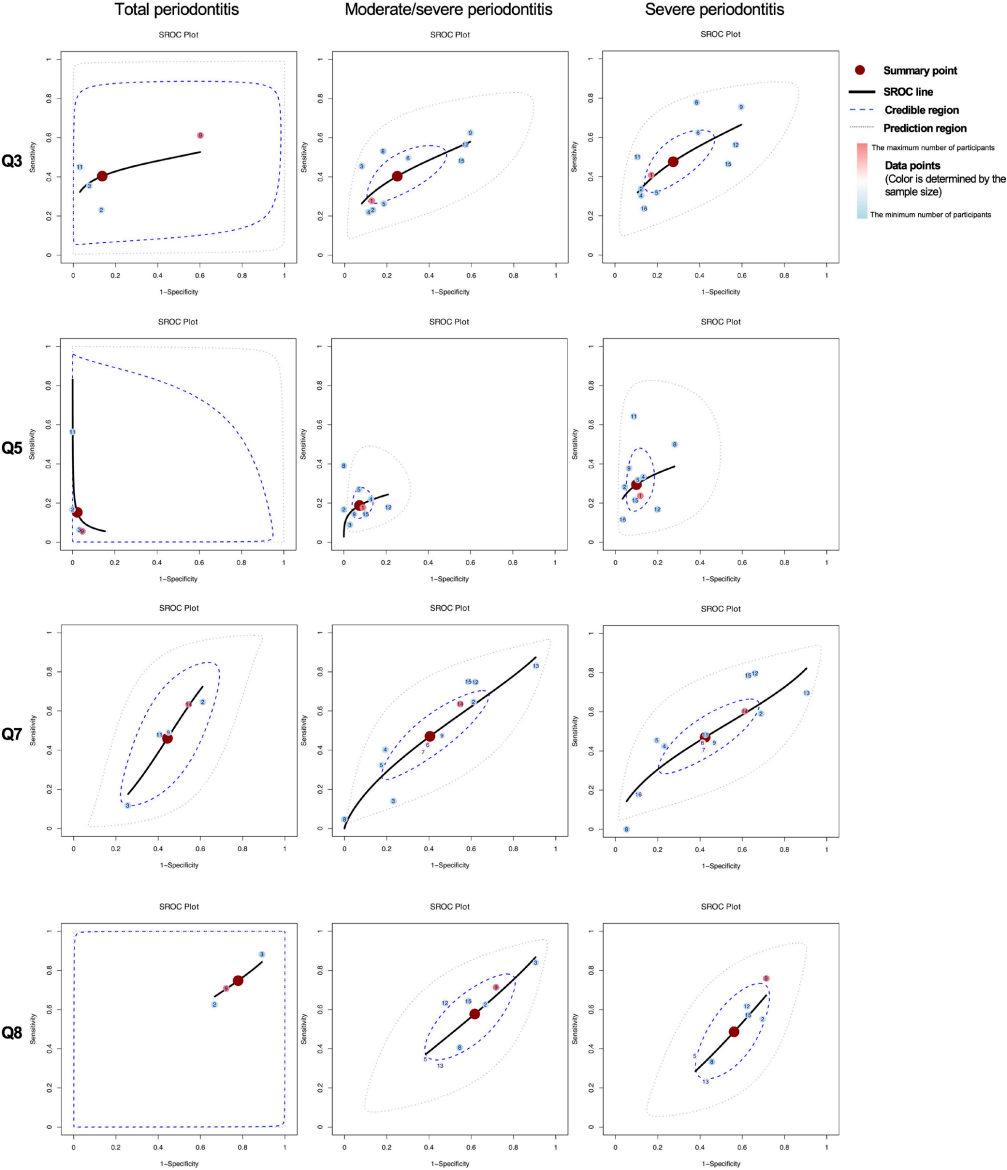
Hierarchical summary receiver operating characteristic (HSROC) curves for Q3, Q5, Q7 and Q8 in the CDC/AAP questionnaire. The question numbers correspond to those in Figure 2. The left column indicates total periodontitis (including mild, moderate, and severe cases), the middle column moderate/severe periodontitis, and the right column severe periodontitis. Each point represents a study. The colour of the study points ranges from blue to red, indicating that the sample size varies from small to large. The numbers on the points correspond to the following study references: (1) Bond et al. (2024); (2) Carra et al. (2018); (3) Cassiano et al. (2024); (4) Cyrino et al. (2011); (5)Eke and Dye (2009); (6) Genco et al. (2007); (7) Genco et al. (2007); (8) Heaton et al. (2017); (9) Iwasaki et al. (2021); (10) Lertpimonchai et al. (2023); (11) Machado et al. (2022); (12) Montero et al. (2020); (13) Taylor et al. (2007); (14) Ueno et al. (2020); (15) Wu et al. (2013); (16) Reiniger et al. (2020).

The HSROC curves, shown in Figures 4 and 5, categorise the eight questions from the CDC/AAP questionnaire into four groups: Q1–Q2 for self‐assessment of periodontal status; Q4 and Q6 for symptoms; Q3 and Q5 for professional diagnosis and treatment history; and Q7–Q8 for health behaviours.

Different definition categories exhibited similar HSROC curve patterns. For example, a convex HSROC curve for Q1 indicates strong diagnostic performance, while shorter curves, like for Q4, reflect low sensitivity but high specificity. Q5 showed high specificity with variable sensitivity, suggesting unstable diagnostic performance. Overall, most items had acceptable specificity but were limited by low sensitivity, especially at higher disease severities, causing the credible region of the HSROC curves to narrow. HSROC curves for self‐designed questions are shown in Figures S26–S40.

### 3.8 Influence Analysis and Publication Bias

The Baujat plots (Figures S2–S40) suggest that studies positioned in the upper right corner may have a significant influence, as they substantially affect both the estimated heterogeneity and the DOR. The contribution of each study to heterogeneity differs across various problem domains. Iwasaki et al. (2021) and Machado et al. (2022) accounted for the highest degree of heterogeneity. The Deeks funnel plots (Figures S2–S40) reveal no significant publication bias among the included studies, except for Q1, Q12 and Q16 in detecting moderate‐to‐severe periodontitis and Q2 in detecting severe periodontitis. We subsequently performed a leave‐one‐out analysis, with detailed results presented in Table S5. This analysis showed that although a few individual studies had a substantial impact on the results, most studies contributed fairly evenly, supporting the overall robustness of the findings. Notably, within the total periodontitis definition category for questions Q1, Q3, Q5 and Q6, some studies exerted a relatively large influence on the outcomes. This may be partly due to the small number of studies included in these analyses, which reduces result stability. Additionally, a high proportion of ‘don't know’ answers reported in Carra et al. (2018) and Iwasaki et al. (2021) for Q1, Q3 and Q5 may have further affected these results.

## 4 Discussion

This systematic review and meta‐analysis offers a comprehensive assessment of the diagnostic accuracy of individual self‐reported questions for periodontitis, integrating evidence from diverse geographic and cultural contexts. Our findings underscore both the potential utility and inherent limitations of these tools, reinforcing and expanding upon previous research in this field. Below, we discuss the key issues that merit further consideration.

A previous meta‐analysis evaluated seven questions for detecting moderate and severe periodontitis (Abbood et al. 2016). With many new studies published since then, we included additional commonly used self‐designed questions beyond the eight‐item CDC/AAP questionnaire, as long as at least three studies were available. In total, 16 questions were analysed for their diagnostic performance across three case definitions.

The eight CDC/AAP questionnaire items can be categorised into four domains in terms of content: overall self‐perception of oral health; symptoms; oral healthcare behaviours; and information received from dental professionals. In contrast, the additional commonly used questions mainly focus on symptoms and behaviours, such as bleeding gums, tooth loss and brushing or check‐up frequency.

The CDC/AAP questionnaire aims to provide a valid and reliable means for monitoring periodontitis, especially in resource‐limited public health programmes (Eke et al. 2013). This approach focuses on feasibility and effectiveness rather than strict diagnostic accuracy. Multiple questions in the survey rely on professional periodontal knowledge. For instance, Q1 (perceived gum disease) and Q3 (periodontal treatment history) require an understanding of specific terms. Some questions, like Q5 (bone loss diagnosis), depend on prior diagnoses. This reliance on terminology can lead to varying answers among participants from different healthcare backgrounds, complicating the assessment of the questionnaire's effectiveness. Consequently, it may be best suited for long‐term studies involving those with previous periodontal care or knowledge. Further research is needed to develop effective screening tools for broader populations.

Regarding sensitivity and specificity, most questions showed higher specificity than sensitivity, except Q2 (oral health rating), Q8 (mouthwash use) and Q10 (tooth loss), which demonstrated notably higher sensitivity. Given the consistently lower sensitivity compared to the specificity of individual questionnaire items, researchers should explore strategic combinations of items to enhance diagnostic validity. This approach warrants careful consideration of the methodological framework for item integration and the potential trade‐offs between sensitivity and specificity in composite measures. Some studies have compared the effectiveness of combining two questions and found that specificity increases when a case is defined as a concordant positive answer. At the same time, sensitivity decreases compared to when defining a case based on a single positive answer to either question (Bond et al. 2024). Current methodological approaches for periodontitis prediction primarily employ logistic regression models that integrate questionnaire items with demographic and risk factor data (Eke and Dye 2009; Heaton et al. 2017; Kapellas et al. 2021). Other researchers have used different approaches, such as assigning equal weights to the number of ‘yes’ answers (Khader et al. 2015; Lertpimonchai et al. 2023) or applying different weights to individual questions in modelling (Carra et al. 2018), selecting the optimal model threshold through sample analysis. Integrating demographic and risk factors to evaluate the combined diagnostic performance of multiple questionnaire items is highly valuable. However, due to the reporting formats in the included studies and limited access to original data, we could not perform such an analysis in this meta‐analysis.

Many researchers now combine questionnaires with salivary biomarkers—such as activated matrix metalloproteinase‐8, protease, chitinase and haemoglobin—to build predictive models, greatly improving diagnostic accuracy (Deng et al. 2024; Maeng et al. 2016; Verhulst et al. 2019). This approach shows promise for future periodontitis screening, especially when paired with machine learning.

This review found many studies using self‐reported questionnaires for periodontitis. However, to improve the consistency and clinical applicability of this systematic review, multiple studies were excluded based on three primary limitations: (i) absence of definitive periodontitis diagnosis; (ii) non‐compliance with either the CDC/AAP criteria or the 2017 classification system; and (iii) insufficient evaluation of diagnostic performance for individual questionnaire items. These issues highlight the need for future research to use standardised diagnostic criteria and calibrated exams to improve quality and comparability.

Although our quantitative synthesis was limited to studies using the CDC/AAP criteria based on full‐mouth periodontitis assessment through comprehensive probing, thus ensuring methodological consistency in the reference standard, significant heterogeneity remained across studies. This was mainly due to sociocultural differences across populations. Firstly, significant differences in the understanding of question content were observed. For example, in Q1, participants from France, Japan, New Zealand and China provided higher ‘Don't know/Refused’ answers than other countries. Furthermore, geographic and cultural heterogeneity significantly influenced diagnostic performance. For instance, studies by Iwasaki et al. (2021) and Wu et al. (2013), based on East Asian populations, demonstrated higher sensitivity than specificity for many questions, unlike most others. Future research should address these local differences by using cognitive interviews, clearer wording and culturally appropriate questions. Additionally, due to the large number of studies based in the United States and the inclusion of large databases such as the NHANES database, the overrepresentation of US‐based studies may limit the generalisability of this review. More studies are needed to validate questionnaires across diverse populations.

When comparing the diagnostic accuracy of the same question across different severity levels of periodontitis, we observed some variations. However, these comparisons should be interpreted cautiously, as the studies included in this meta‐analysis for different stages of periodontitis do not fully overlap. Consequently, the conclusions drawn from these comparisons remain preliminary. Furthermore, the number of studies assessing moderate‐to‐severe and severe periodontitis was significantly greater than those evaluating total periodontitis, resulting in narrower credible intervals and more stable estimates for the former, emphasising the need for further research on the diagnostic performance of self‐reported questionnaires for total periodontitis. Additionally, future studies should compare the diagnostic accuracy of self‐reported questionnaires across different stages of periodontitis within the same study. Such an approach would enable more reliable comparisons under consistent criteria and workflow.

This systematic review and meta‐analysis highlight the potential of self‐reported questionnaires as practical tools for large‐scale periodontitis surveillance while emphasising key limitations in their diagnostic accuracy and cross‐cultural applicability. The CDC/AAP questionnaire and other self‐designed items consistently exhibited higher specificity than sensitivity across diverse populations, with variations in comprehension and answer patterns influenced by geographic and sociocultural factors. Key questions related to self‐perceived periodontal health, professional diagnoses and tooth loss showed moderate discriminatory ability, yet low sensitivity remains a persistent barrier to reliable early detection. Substantial heterogeneity in reference standards and methodological inconsistencies across studies further challenge the generalisability of findings. Future efforts should prioritise culturally adaptive questionnaire designs, integrating biomarkers and machine learning models and standardised validation protocols to enhance diagnostic precision. By addressing these gaps, self‐reported tools could evolve into robust, accessible instruments for global periodontitis screening, bridging the divide between resource‐intensive clinical examinations and public health needs.

## Author Contributions

Mengning Bi and Yu Xie contributed to protocol development, data collection, analysis and interpretation and manuscript preparation. Xiaoyu Yu and Hairui Li contributed to data analysis, and manuscript preparation. Yuan Li contributed to protocol development, data interpretation, and manuscript preparation. Maurizio S. Tonetti devised this study and contributed to protocol development, data interpretation and manuscript preparation. All authors contributed to manuscript revision, gave their final approval and agreed to be accountable for all aspects of the work.

## Conflicts of Interest

Maurizio S. Tonetti received grant support and/or personal fees from Geistlich Pharma AG (Switzerland), Straumann AG (Switzerland) and Nobel Biocare SA (Sweden), which are unrelated to the present work. The other authors declare no conflicts of interest.

## Supporting information


**Data S1:** Supporting Information.

## Data Availability

Data sharing not applicable to this article as no datasets were generated or analysed during the current study.
