# Supplementary material for: Diagnostic Accuracy of Self‐Reported Questionnaires for Detecting Periodontitis Across Multiple Cultures and Geographic Locations: A Systematic Review and Meta‐Analysis
Source: J Clin Periodontol. 2025 Aug 8;52(11):1510–28. doi: 10.1111/jcpe.70002 (PMC12531362; doi:10.1111/jcpe.70002)
Supplement: Supplementary file 1 — Data S1: Supporting Information. [file JCPE-52-1510-s001.docx]

**Appendix**

**Diagnostic accuracy of self­reported questionnaires for detecting periodontitis across multiple cultures and geographic locations: A systematic review and meta-analysis**

Mengning Bi^1,2^, Yu Xie^1,2^, Xiaoyu Yu^1,2^, Hairui Li^1,2^, Yuan Li^1,2,3^, Maurizio S. Tonetti^1,2,4^

^1^ Shanghai PerioImplant Innovation Center, Institute for Oral, Craniofacial and Sensory Research, Ninth People's Hospital, Shanghai Jiao Tong University School of Medicine, Shanghai, China

^2^ National Clinical Research Center of Oral Diseases and National Center of Stomatology, College of Stomatology, Shanghai Jiao Tong University School of Medicine, Shanghai, China

^3^ Department of Oral Implantology, Ninth People's Hospital, Shanghai, China

^4^ European Research Group on Periodontology (ERGOPerio), Genova, Italy

**Correspondence**: Maurizio S. Tonetti, Perio-Implant Innovation Center, 4F Building 1, 115 Jinzun Road, Pudong Research Campus, Shanghai Jiao Tong University School of Medicine, 200115, Shanghai, China.

email: [maurizio.tonetti@ergoperio.eu](mailto:maurizio.tonetti@ergoperio.eu)

**Table S1. Search strategy (on 03/04/2025)**

| **Search strategy in PubMed** | | |
| --- | --- | --- |
| #1 | ((("Periodontitis"[Mesh]) OR (periodontitis [Title/Abstract])) OR (periodontal[Title/Abstract])) OR (periodontal disease[Title/Abstract]) | 100368 |
| #2 | (((((("Self Report"[Mesh]) OR ("Self-Assessment"[Mesh]))) OR (questionnaire[Title/Abstract])) OR (self-reported[Title/Abstract])) OR (questionnaires[Title/Abstract])) OR (Self Report[Title/Abstract])) OR (Self-Assessment[Title/Abstract]) | 934323 |
| #3 | ((((((((("Risk Assessment"[Mesh]) OR ("Prognosis"[Mesh])) OR ("Predictive Value of Tests"[Mesh])) OR ("Odds Ratio"[Mesh])) OR (sensitivity[Title/Abstract])) OR (specificity[Title/Abstract])) OR (Risk Assessment[Title/Abstract])) OR (Prognosis[Title/Abstract])) OR (Predictive Value of Tests[Title/Abstract])) OR (Odds Ratio[Title/Abstract]) | 4317122 |
| #4 | #1 AND #2 AND #3 | 751 |
| **Search strategy in Embase** | | |
| #1 | 'periodontitis' OR 'periodont*' OR 'periodontal health' OR 'periodontal disease' | 183247 |
| #2 | 'self-assessment' OR 'self-report' OR 'self-reported' OR 'questionnaire' | 1605252 |
| #3 | 'risk assessment' OR 'prognosis' OR 'predictive value of tests' OR 'odds ratio' OR 'sensitivity' OR 'specificity' | 4792833 |
| #4 | #1 AND #2 AND #3 | 1253 |
| **Search strategy in Cochrane Library** | | |
| #1 | (Periodontitis OR periodontal OR "periodontal disease"):ti,ab,kw | 16254 |
| #2 | ("Self Report" OR Self-Assessment OR self-reported OR questionnaire):ti,ab,kw | 195652 |
| #3 | ("Risk Assessment" OR Prognosis OR "Predictive Value of Tests" OR "Odds Ratio" OR sensitivity OR specificity):ti,ab,kw | 180345 |
| #4 | #1 AND #2 AND #3 | 109 |

**Table S2. Items in the CDC/AAP questionnaire**

| **ID** | **Context** |
| --- | --- |
| **1** | **Do you think you might have gum disease? (Yes, No, Refused, Don't Know)** |
| **2** | **Overall, how would you rate the health of your teeth and gums? (Excellent, Very Good, Good, Fair, Poor, Refused, Don't**  **Know)** |
| **3** | **Have you ever had treatment for gum disease such as scaling and root planing, sometimes called "deep cleaning"? (Yes, No, Refused, Don't Know)** |
| **4** | **Have you ever had any teeth become loose on their own, without an injury? (Yes, No, Refused, Don't Know)** |
| **5** | **Have you ever been told by a dental professional that you lost bone around your teeth? (Yes, No, Refused, Don't Know)** |
| **6** | **During the past 3 months, have you noticed a tooth that doesn't look right? (Yes, No, Refused, Don't Know)** |
| **7** | **Aside from brushing your teeth with a toothbrush, in the last seven days, how many times did you use dental floss or any other device to clean between your teeth? (Number of Days, Refused)** |
| **8** | **Aside from brushing your teeth with a toothbrush, in the last 7 days, how many times did you use mouthwash or other dental rinse product that you use to treat dental disease or dental problems? (Number of Days, Refused)** |

**Table S3. Question list**

| **Categories** | **Questions** | **Studies** |
| --- | --- | --- |
| **Overall self-perception** | Do you think you might have gum disease? | Bond et al. 2024, Carra et al. 2018, Cassiano et al. 2024, Cyrino et al. 2011, Deng et al. 2021, Eke et al. 2009, Heaton et al. 2017, Iwasaki et al. 2021, Kapellas et al. 2020, Lertpimonchai et al. 2023, Machado et al. 2022, Montero et al. 2020, Reiniger et al. 2020, Sim et al. 2022, Taylor et al. 2007, Ueno et al. 2020, Wu et al. 2013. |
|  | How likely is it that you will get gum disease? | Taylor et al. 2007 |
|  | Overall, how would you rate the health of your teeth and gums? | Bond et al. 2024, Carra et al. 2018, Cassiano et al. 2024, Cyrino et al. 2011, Deng et al. 2021, Eke et al. 2009, Enevold et al. 2023, Heaton et al. 2017, Iwasaki et al. 2021, Kapellas et al. 2020, Machado et al. 2022, Montero et al. 2020, Reiniger et al. 2020, Sim et al. 2022, Taylor et al. 2007 |
|  | Do you have "gingivitis" currently or have you ever had it before | Wu et al. 2013 |
| **Symptoms** | Have you ever had any teeth become loose on their own, without an injury? | Bond et al. 2024, Carra et al. 2018, Cassiano et al. 2024, Deng et al. 2021, Eke et al. 2009, Heaton et al. 2017, Iwasaki et al. 2021, Kapellas et al. 2020, Lertpimonchai et al. 2023, Machado et al. 2022, Montero et al. 2020, Reiniger et al. 2020, Sim et al. 2022, Taylor et al. 2007, Ueno et al. 2020, Wu et al. 2013 |
|  | During the past 3 months, have you noticed a tooth that doesn’t look right? | Bond et al. 2024, Carra et al. 2018, Deng et al. 2021, Eke et al. 2009, Heaton et al. 2017, Iwasaki et al. 2021, Kapellas et al. 2020, Machado et al. 2022, Montero et al. 2020, Sim et al. 2022, Taylor et al. 2007 |
|  | Do you have bleeding gums now? | Genco et al. 2007, Machado et al. 2022, Ueno et al. 2020 |
|  | Have your gums bled recently? | Carra et al. 2018, Genco et al. 2007, Iwasaki et al. 2021, Machado et al. 2022, Taylor et al. 2007, Wu et al. 2013 |
|  | Do you have bleeding on brushing? | Lertpimonchai et al. 2023, Wu et al. 2013 |
|  | Do you have food impaction between your teeth? | Carra et al. 2018 |
|  | Have you ever had a recession of your gums, so that teeth appear longer now? | Carra et al. 2018, Machado et al. 2022, Wu et al. 2013 |
|  | Do you think that you can see more roots of teeth than in the past? | Carra et al. 2018, Machado et al. 2022 |
|  | tooth loss | Cassiano et al. 2024, Genco et al. 2007, Montero et al. 2020, Taylor et al. 2007 |
|  | Have you lost teeth because of mobility? | Cyrino et al. 2011, Machado et al. 2022, Reiniger et al. 2020, Wu et al. 2013 |
|  | Upper/lower tooth count | Sim et al. 2022 |
|  | How many of your own teeth do you have left? | Enevold et al. 2023 |
|  | Have you ever had chronic malodour or bad taste? | Cassiano et al. 2024, Genco et al. 2007, Lertpimonchai et al. 2023, Wu et al. 2013 |
|  | Have you ever noticed that your front teeth have moved forward? | Cyrino et al. 2011,Reiniger et al. 2020, Wu et al. 2013 |
|  | Are you satisfied with chewing? | Genco et al. 2007 |
|  | Do you have sore gums? | Genco et al. 2007, Lertpimonchai et al. 2023, Machado et al. 2022, Taylor et al. 2007, Wu et al. 2013 |
|  | Do you have gum boil? | Genco et al. 2007 |
|  | Have you ever had any teeth being hypersensitive to external stimuli (cold, heat, acid) | Wu et al. 2013 |
|  | Thinks gums look swollen or puffy? | Lertpimonchai et al. 2023 |
|  | Do you have pus from gum? | Lertpimonchai et al. 2023 |
|  | Do you have calculous deposits? | Ueno et al. 2020, Wu et al. 2013 |
|  | Do you have hurting teeth? | Taylor et al. 2007, Wu et al. 2013 |
|  | Have you ever had oral ulcer? | Wu et al. 2013 |
|  | Have you ever had painful tongue? | Wu et al. 2013 |
| **Oral health care** | Aside from brushing your teeth with a toothbrush, in the last 7 days, how many times did you use dental floss or any other device to clean between your teeth? | Carra et al. 2018, Cassiano et al. 2024, Cyrino et al. 2011, Deng et al. 2021, Eke et al. 2009, Enevold et al. 2023, Genco et al. 2007, Heaton et al. 2017, Iwasaki et al. 2021, Kapellas et al. 2020, Machado et al. 2022, Montero et al. 2020, Sim et al. 2022, Taylor et al. 2007, Ueno et al. 2020, Wu et al. 2013, Reiniger et al. 2020, Ueno et al. 2020 |
|  | Aside from brushing your teeth with a toothbrush, in the last 7 days, how many times did you use mouthwash or other dental rinse product that you use to treat dental disease or dental problems? | Carra et al. 2018, Cassiano et al. 2024, Deng et al. 2021, Eke et al. 2009, Enevold et al. 2023, Heaton et al. 2017, Iwasaki et al. 2021, Kapellas et al. 2020, Montero et al. 2020, Sim et al. 2022, Taylor et al. 2007, Wu et al. 2013 |
|  | Frequency of tooth brushing | Cyrino et al. 2011, Enevold et al. 2023, Genco et al. 2007 |
|  | Frequency of Dental checkups | Enevold et al. 2023, Genco et al. 2007, Taylor et al. 2007 |
|  | Do you use toothpick to clean your teeth? | Enevold et al. 2023, Taylor et al. 2007, Wu et al. 2013 |
|  | Unable to brush teeth | Taylor et al. 2007 |
|  | Usual dental care source | Taylor et al. 2007 |
|  | Dental insurance status | Taylor et al. 2007 |
| Informed by dentists | Has a dentist ever told you that you have periodontal disease? | Ueno et al. 2020, Wu et al. 2013 |
|  | Have you ever been told by a dental professional that you lost bone around your teeth? | Bond et al. 2024, Carra et al. 2018, Cassiano et al. 2024, Cyrino et al. 2011, Deng et al. 2021, Eke et al. 2009, Heaton et al. 2017, Iwasaki et al. 2021, Kapellas et al. 2020, Machado et al. 2022, Montero et al. 2020, Reiniger et al. 2020, Sim et al. 2022, Wu et al. 2013 |
|  | Have you ever been told that you need periodontal or gum treatment? | Wu et al. 2013 |
| Dental treatment experience | Have you ever had treatment for gum disease, such as scaling and root planing, sometimes called "deep" cleaning? | Bond et al. 2024, Carra et al. 2018, Cassiano et al. 2024, Cyrino et al. 2011, Deng et al. 2021, Eke et al. 2009, Enevold et al. 2023, Genco et al. 2007, Heaton et al. 2017, Iwasaki et al. 2021, Kapellas et al. 2020, Machado et al. 2022, Montero et al. 2020, Reiniger et al. 2020, Sim et al. 2022, Wu et al. 2013 |
|  | Have you ever had periodontal surgery? | Cyrino et al. 2011, Deng et al. 2021, Genco et al. 2007, Reiniger et al. 2020, Wu et al. 2013 |

**Figure S1. (a) Risk of bias and applicability concerns graph assessed according to QUADAS-2 domains: patient selection, index test, reference standard, and flow and timing. (b) Summary of risk of bias and applicability concerns across included studies based on the same QUADAS-2 domains.**

**
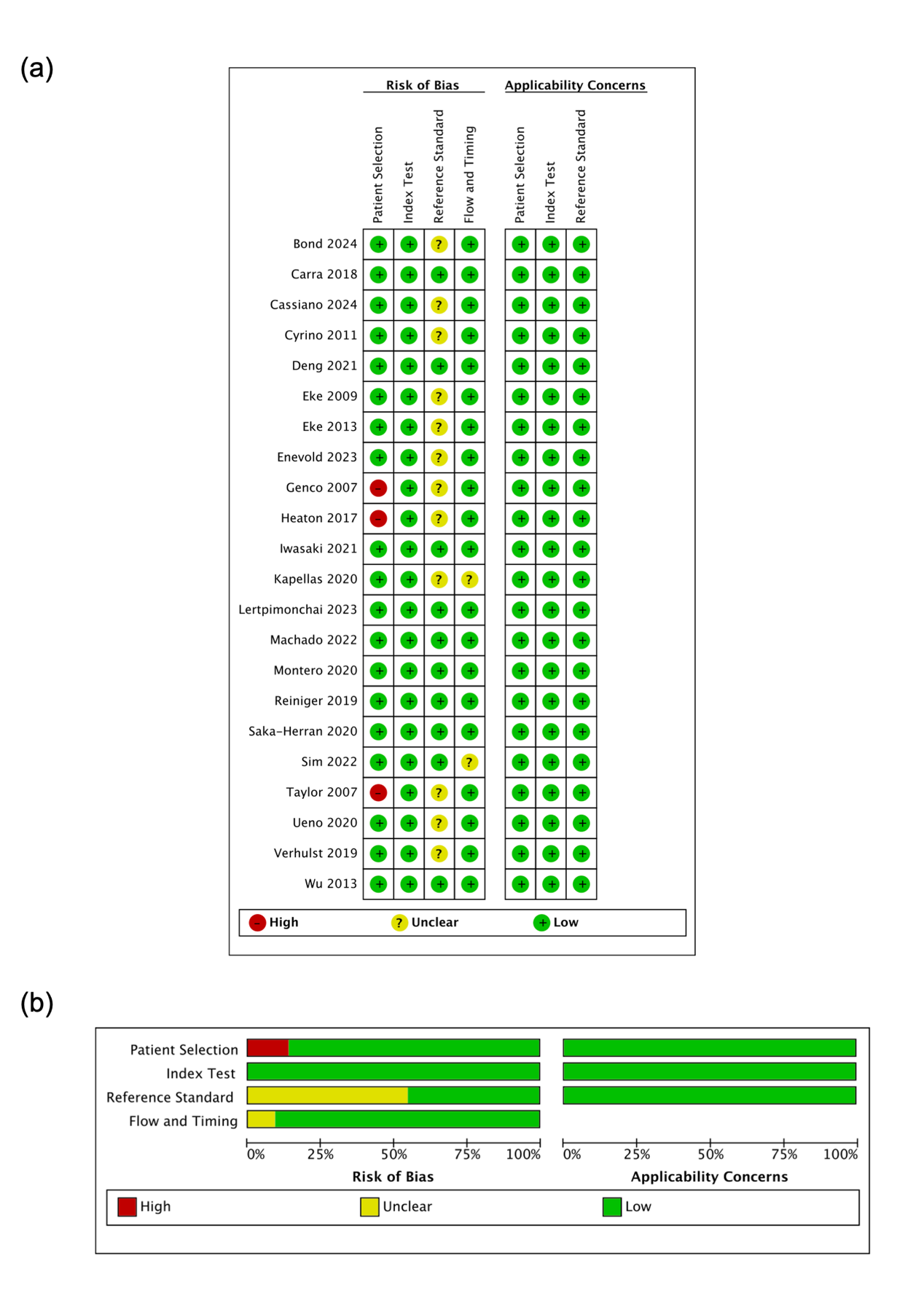
**

**Table S4. Heterogeneity between studies**

| **Questions** | **Clinical diagnose** | **Sensitivity-Q** | **Sensitivity-P_value** | **Sensitivity-I^2^** | **Specificity-Q** | **Specificity-P_value** | **Specificity-I^2^** |
| --- | --- | --- | --- | --- | --- | --- | --- |
| Q1: Do you think you might have gum disease? | Total periodontitis | 34.42 | 0 | 91.3 | 44.69 | 0 | 93.3 |
|  | Moderate-to-severe periodontitis | 201.9 | 0 | 95.5 | 197 | 0 | 95.4 |
|  | Severe periodontitis | 125.44 | 0 | 91.2 | 386.2 | 0 | 97.2 |
| Q2: Overall, how would you rate the health of your teeth and gums? | Total periodontitis | 32.52 | 0 | 90.8 | 65.74 | 0 | 95.4 |
|  | Moderate-to-severe periodontitis | 20.9 | 0 | 90.4 | 63.96 | 0 | 96.9 |
|  | Severe periodontitis | 16.54 | 0 | 81.9 | 5.29 | 0.15 | 43.3 |
| Q3: Have you ever had treatment for gum disease such as scaling and root planing, sometimes called “deep cleaning”? | Total periodontitis | 114.12 | 0 | 97.4 | 137.81 | 0 | 97.8 |
|  | Moderate-to-severe periodontitis | 183.54 | 0 | 98.4 | 856.23 | 0 | 99.6 |
|  | Severe periodontitis | 11.59 | 0.01 | 74.1 | 29.65 | 0 | 89.9 |
| Q4: Have you ever had any teeth become loose on their own, without an injury? | Total periodontitis | 25.56 | 0 | 84.3 | 42.4 | 0 | 90.6 |
|  | Moderate-to-severe periodontitis | 22.7 | 0 | 91.2 | 72.32 | 0 | 97.2 |
|  | Severe periodontitis | 25.02 | 0 | 92 | 18.52 | 0 | 89.2 |
| Q5: Have you ever been told by a dental professional that you lost bone around your teeth? | Total periodontitis | 14.62 | 0 | 86.3 | 53.51 | 0 | 96.3 |
|  | Moderate-to-severe periodontitis | 39.27 | 0 | 92.4 | 56.16 | 0 | 94.7 |
|  | Severe periodontitis | 212.34 | 0 | 97.6 | 325.41 | 0 | 98.5 |
| Q6: During the past 3 months, have you noticed a tooth that doesn’t look right? | Total periodontitis | 98.76 | 0 | 97 | 221.66 | 0 | 98.6 |
|  | Moderate-to-severe periodontitis | 258.3 | 0 | 96.5 | 631.35 | 0 | 98.6 |
|  | Severe periodontitis | 94.51 | 0 | 89.4 | 765.41 | 0 | 98.7 |
| Q7: Do you use dental floss/other device to clean your teeth? | Total periodontitis | 69.49 | 0 | 95.7 | 88.39 | 0 | 96.6 |
|  | Moderate-to-severe periodontitis | 330.41 | 0 | 97.3 | 603.39 | 0 | 98.5 |
|  | Severe periodontitis | 120.85 | 0 | 91.7 | 864.89 | 0 | 98.8 |
| Q8: Do you use mouthwash or other dental rinse product to treat dental disease or dental problems? | Total periodontitis | 72.46 | 0 | 95.9 | 6.74 | 0.08 | 55.5 |
|  | Moderate-to-severe periodontitis | 142.08 | 0 | 93.7 | 20.88 | 0.01 | 56.9 |
|  | Severe periodontitis | 124.52 | 0 | 90.4 | 107.62 | 0 | 88.8 |
| Q9: Do you have bleeding gums? | Total periodontitis | 125.39 | 0 | 97.6 | 1 | 0.8 | 0 |
|  | Moderate-to-severe periodontitis | 30.06 | 0 | 73.4 | 26.46 | 0 | 69.8 |
|  | Severe periodontitis | 71.76 | 0 | 87.5 | 69.85 | 0 | 87.1 |
| Q10: Do you have tooth loss? | Moderate-to-severe periodontitis | 74.85 | 0 | 97.3 | 10.63 | 0 | 81.2 |
|  | Severe periodontitis | 124.48 | 0 | 95.2 | 126.54 | 0 | 95.3 |
| Q11: Do you have tooth loss because of mobility? | Severe periodontitis | 89.77 | 0 | 91.1 | 210.74 | 0 | 96.2 |
| Q12: Are you satisfied with your breath/taste? | Moderate-to-severe periodontitis | 74.78 | 0 | 94.7 | 38.04 | 0 | 89.5 |
|  | Severe periodontitis | 373.34 | 0 | 97.1 | 365.72 | 0 | 97 |
| Q13: Have you ever had periodontal surgery? | Moderate-to-severe periodontitis | 165.65 | 0 | 92.8 | 748.69 | 0 | 98.4 |
|  | Severe periodontitis | 14.76 | 0 | 86.5 | 15.17 | 0 | 86.8 |
| Q14: Frequency of tooth brushing | Severe periodontitis | 111.55 | 0 | 93.7 | 164.36 | 0 | 95.7 |
| Q15: Frequency of Dental checkups | Moderate-to-severe periodontitis | 36.24 | 0 | 83.4 | 177.65 | 0 | 96.6 |
|  | Severe periodontitis | 89.18 | 0 | 96.6 | 40.55 | 0 | 92.6 |
| Q16: Do you have sore gums? | Moderate-to-severe periodontitis | 247.2 | 0 | 97.6 | 104.38 | 0 | 94.3 |
|  | Severe periodontitis | 126.05 | 0 | 94.4 | 246.77 | 0 | 97.2 |

**Figure S2. Q1 "Do you think you might have gum disease?" for detecting total periodontitis: (a) Forest plot of sensitivity, (b) Forest plot of specificity, (c) Forest plot of diagnostic odds ratio (DOR), (d) Baujat plot, (e) Deeks's funnel plot.**

**
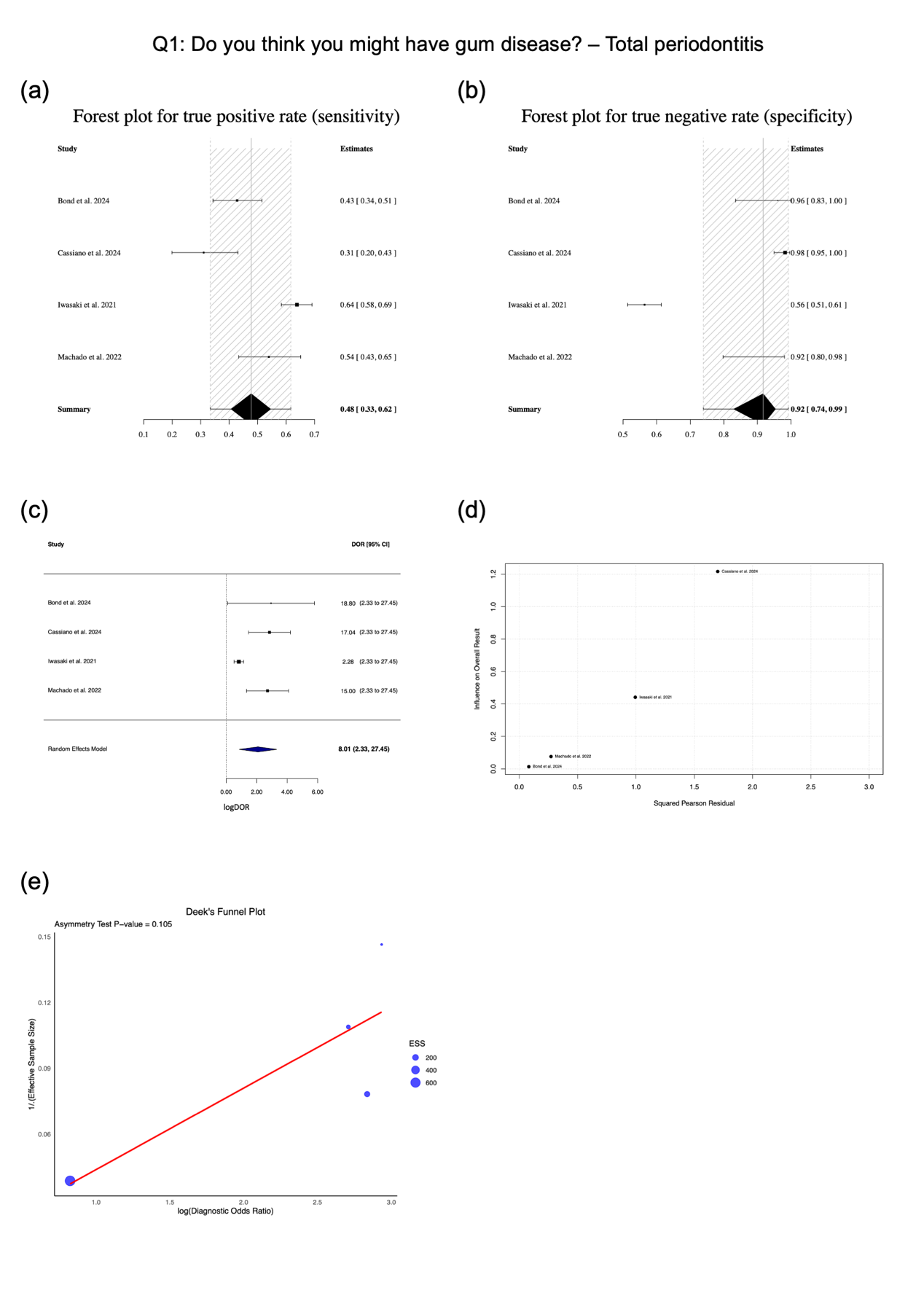
**

**Figure S3. Q1 "Do you think you might have gum disease?" for detecting moderate-to-severe periodontitis: (a) Forest plot of sensitivity, (b) Forest plot of specificity, (c) Forest plot of diagnostic odds ratio (DOR), (d) Baujat plot, (e) Deeks's funnel plot.**

**
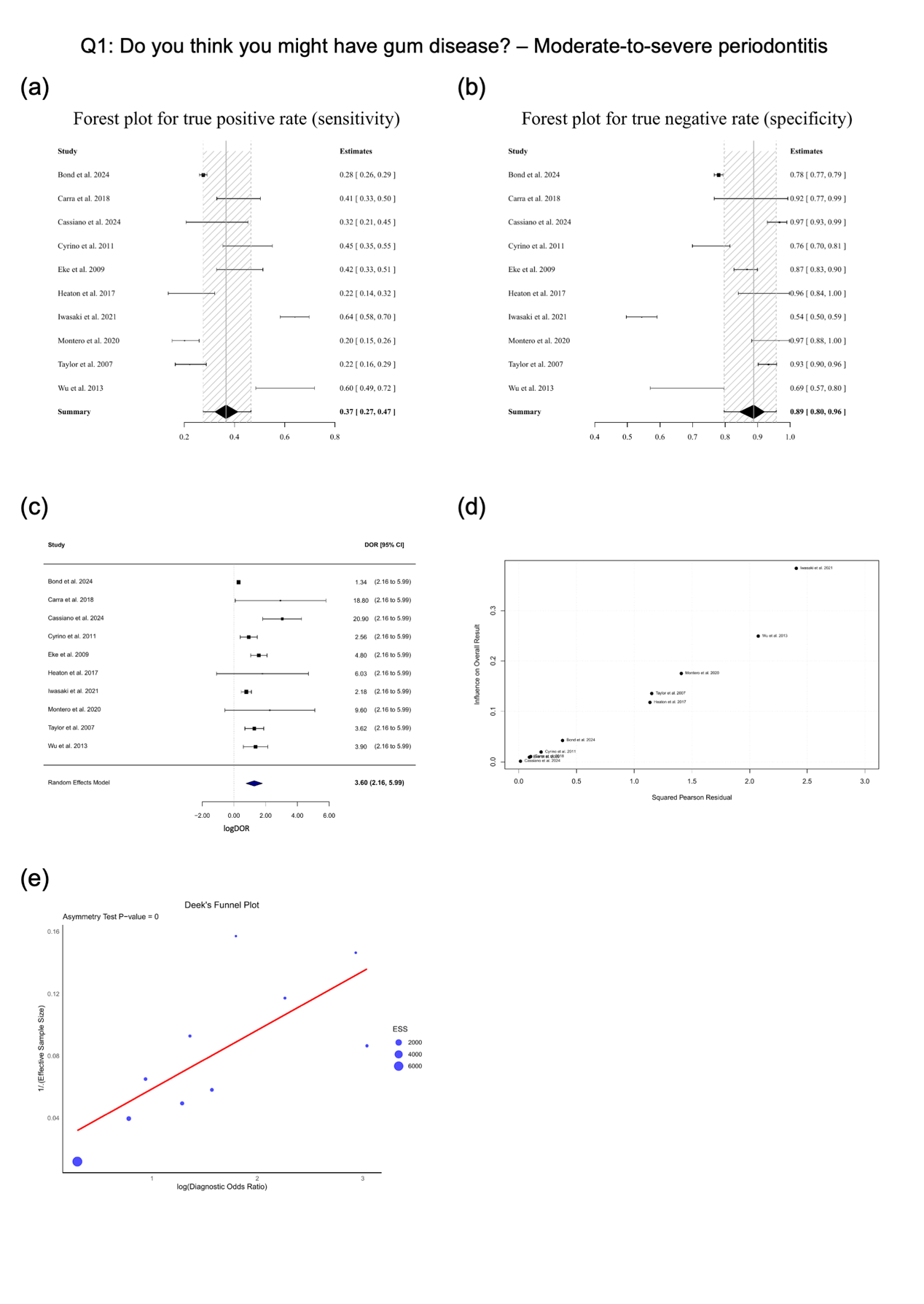
**

**Figure S4. Q1 "Do you think you might have gum disease?" for detecting severe periodontitis: (a) Forest plot of sensitivity, (b) Forest plot of specificity, (c) Forest plot of diagnostic odds ratio (DOR), (d) Baujat plot, (e) Deeks's funnel plot.**

**
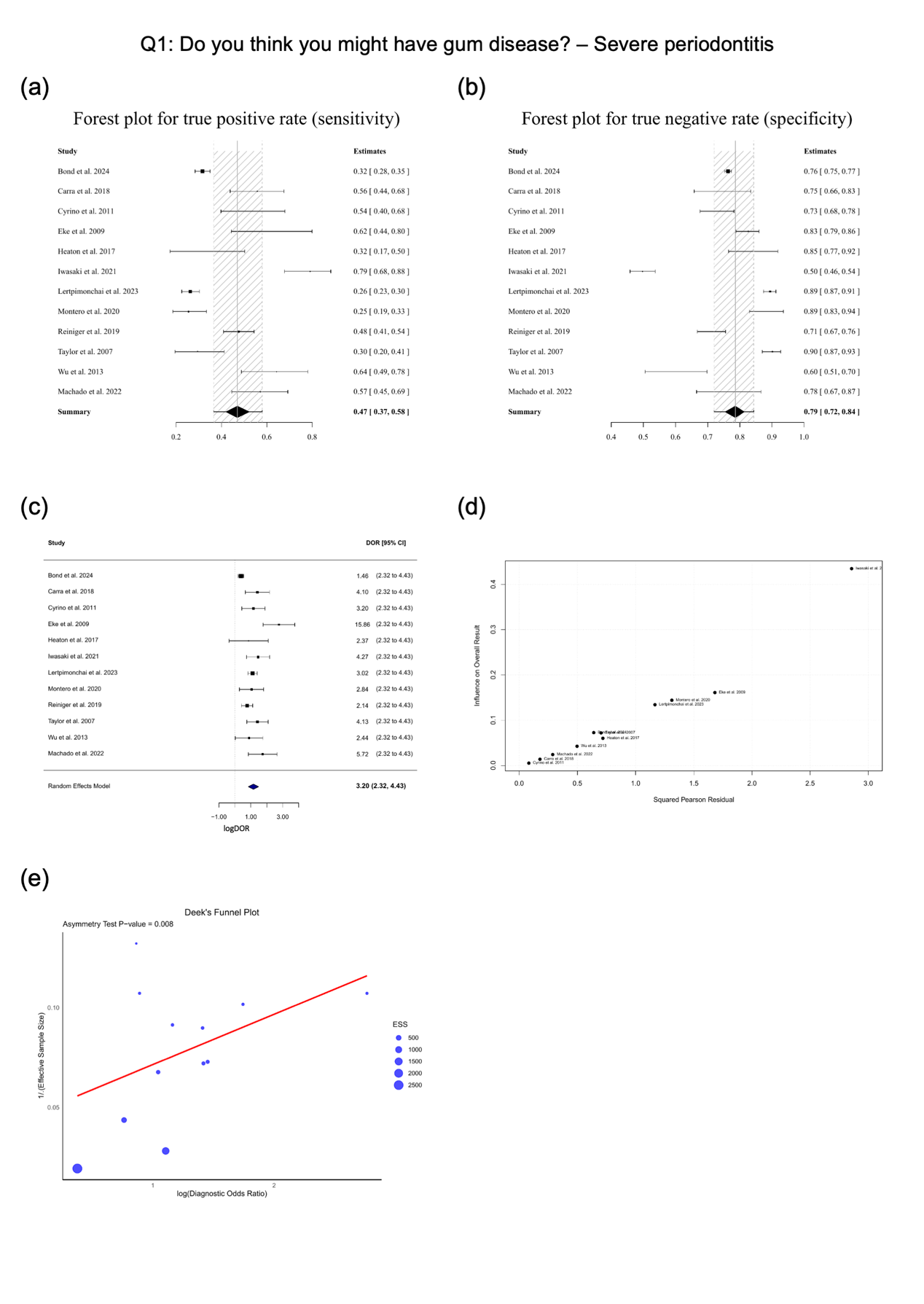
**

**Figure S5. Q2 "** **Overall, how would you rate the health of your teeth and gums?" for detecting total periodontitis: (a) Forest plot of sensitivity, (b) Forest plot of specificity, (c) Forest plot of diagnostic odds ratio (DOR), (d) Baujat plot, (e) Deeks's funnel plot.**

**
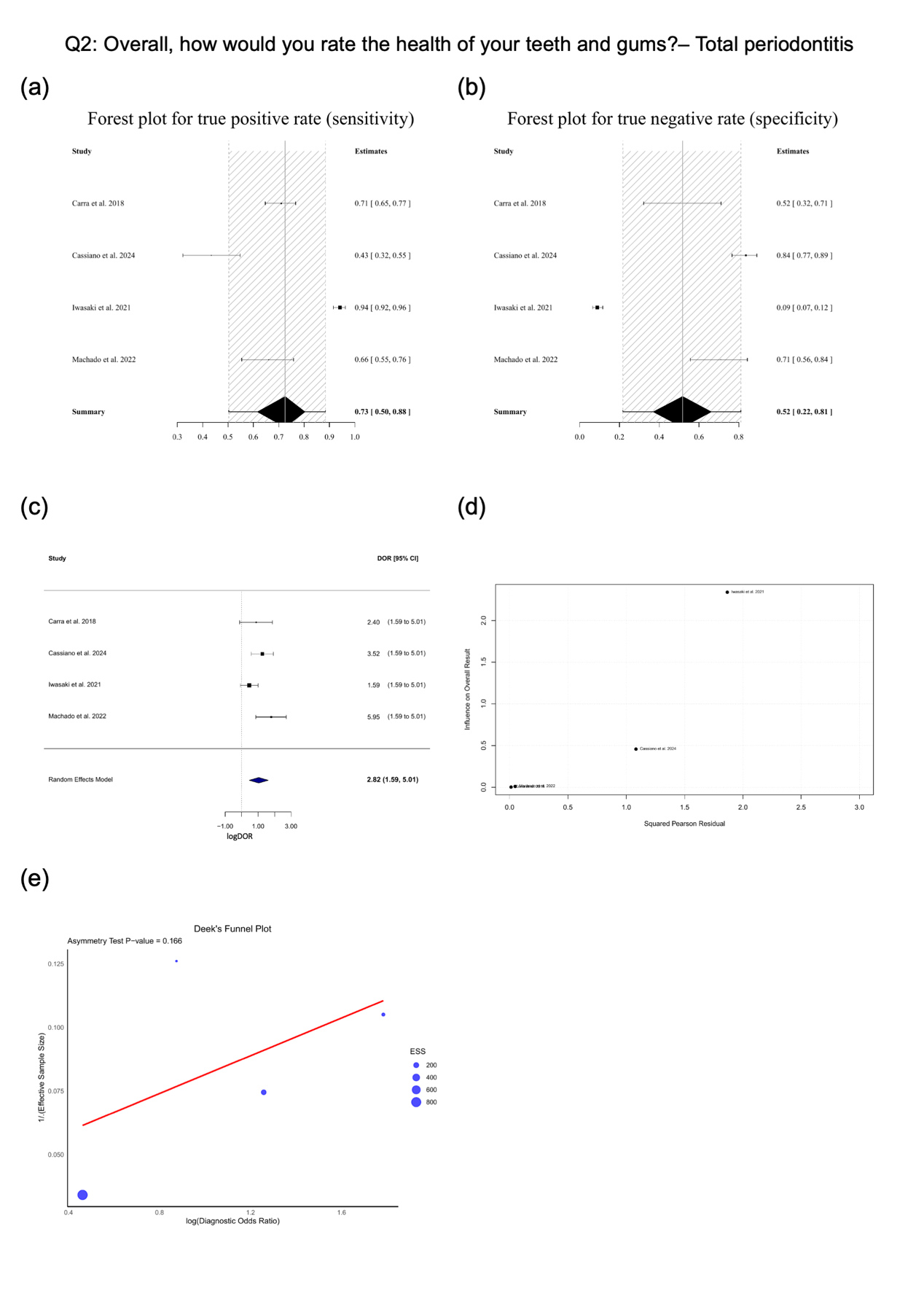
**

**Figure S6. Q2 "** **Overall, how would you rate the health of your teeth and gums?" for detecting moderate-to-severe periodontitis: (a) Forest plot of sensitivity, (b) Forest plot of specificity, (c) Forest plot of diagnostic odds ratio (DOR), (d) Baujat plot, (e) Deeks's funnel plot.**

**
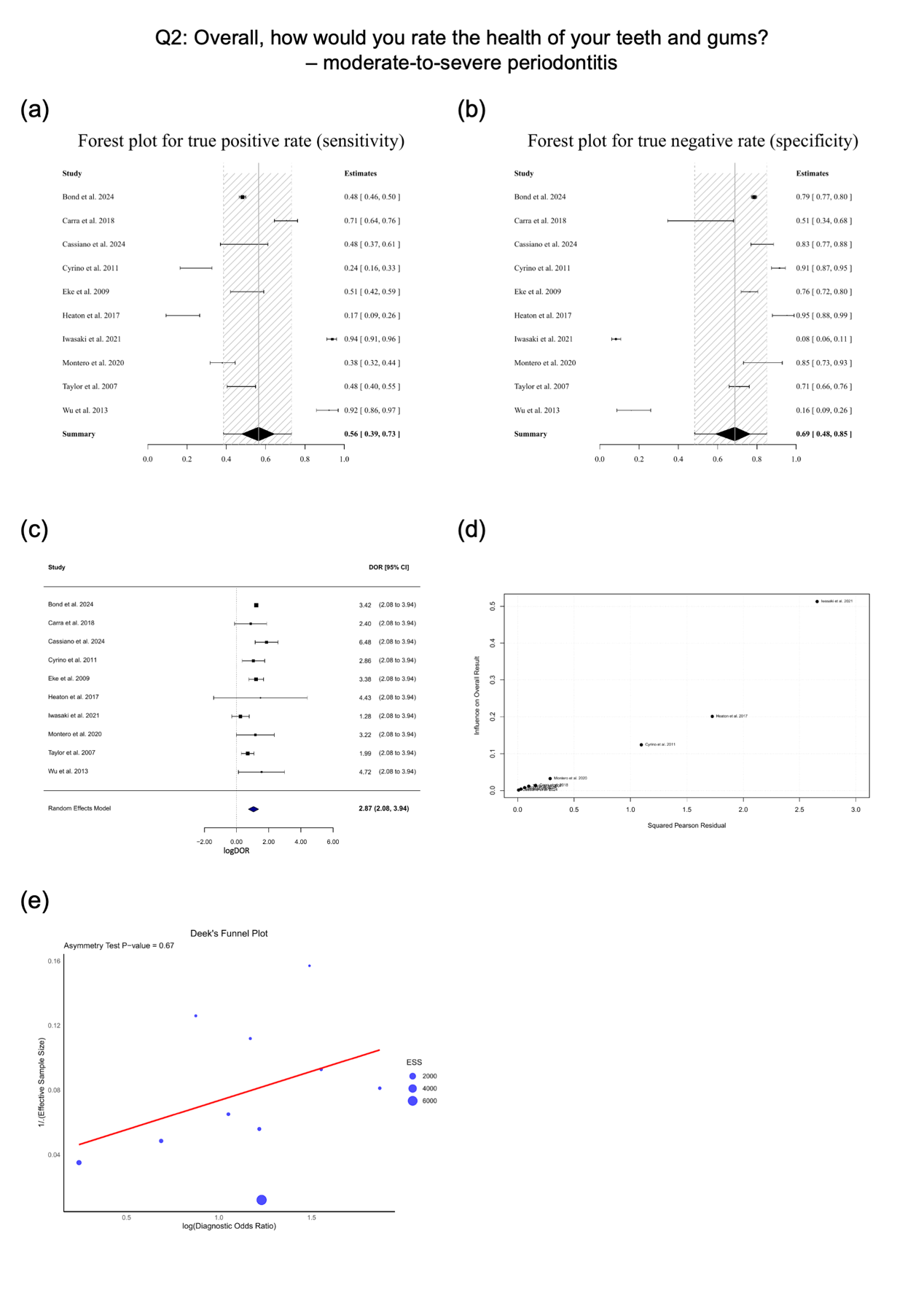
**

**Figure S7. Q2 "** **Overall, how would you rate the health of your teeth and gums?" for detecting severe periodontitis: (a) Forest plot of sensitivity, (b) Forest plot of specificity, (c) Forest plot of diagnostic odds ratio (DOR), (d) Baujat plot, (e) Deeks's funnel plot.**

**
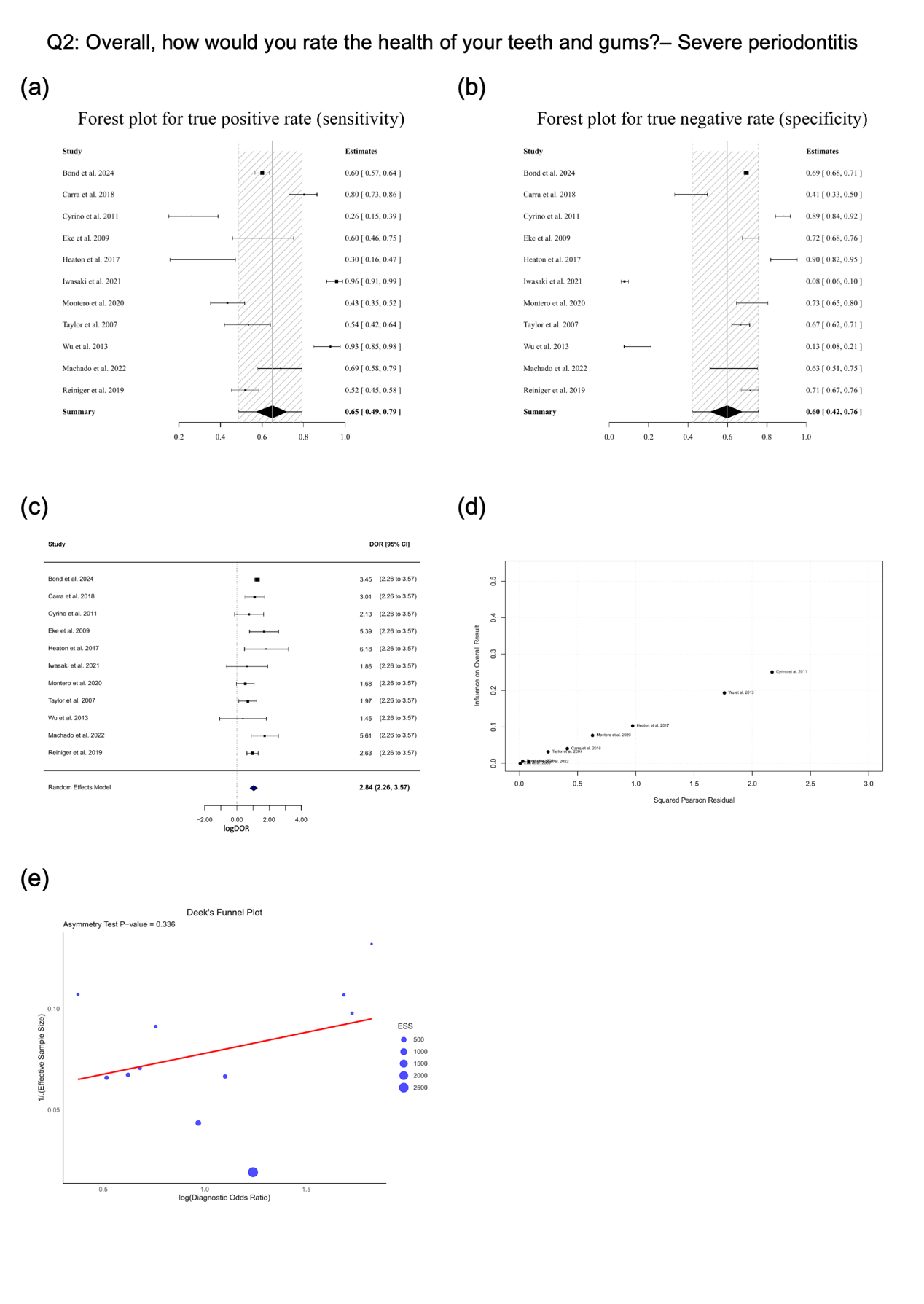
**

**Figure S8. Q3 "Have you ever had treatment for gum disease such as scaling and root planing, sometimes called ‘deep cleaning’?" for detecting total periodontitis: (a) Forest plot of sensitivity, (b) Forest plot of specificity, (c) Forest plot of diagnostic odds ratio (DOR), (d) Baujat plot, (e) Deeks's funnel plot.**

**
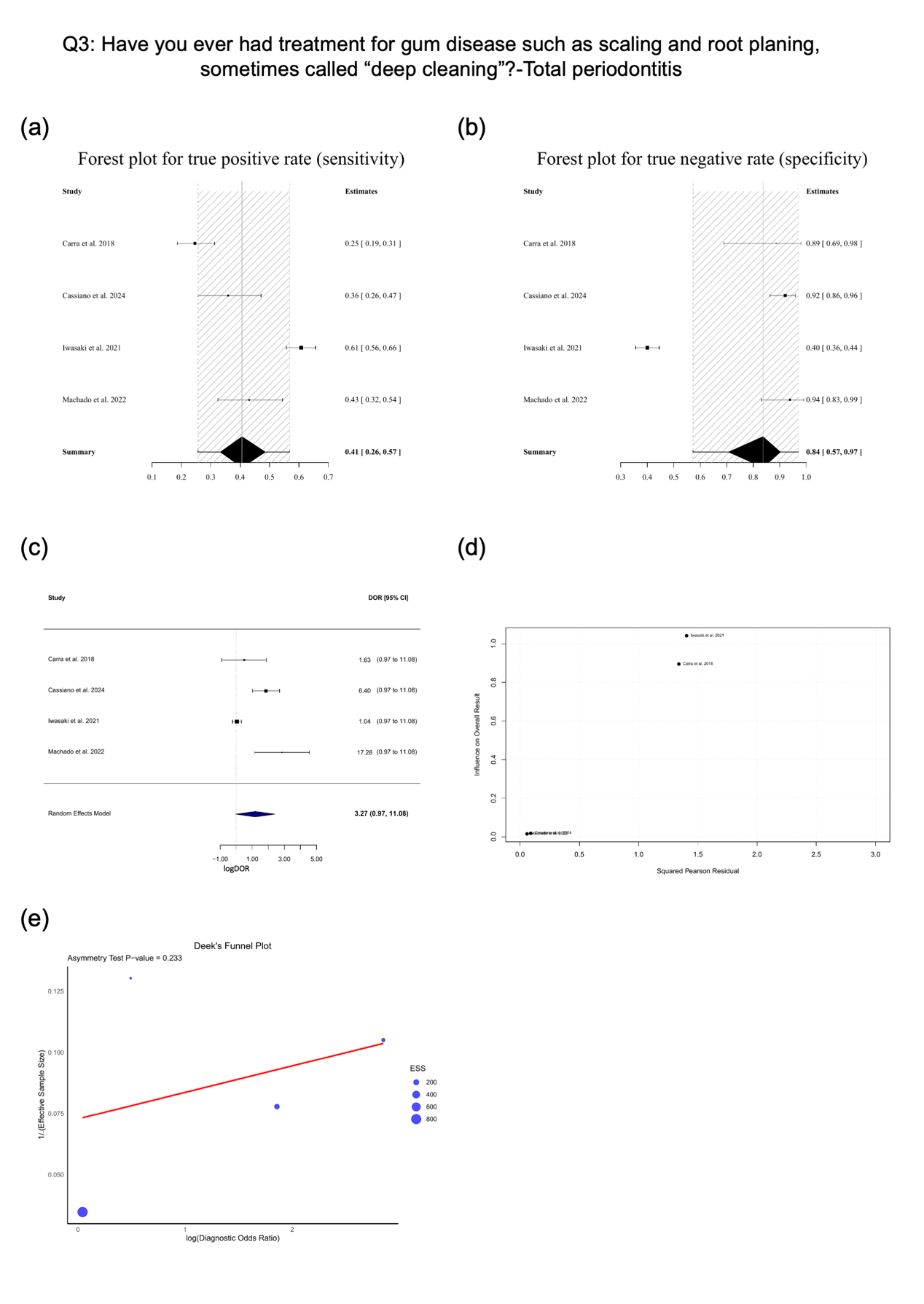
**

**Figure S9. Q3 "Have you ever had treatment for gum disease such as scaling and root planing, sometimes called ‘deep cleaning’?" for detecting moderate-to-severe periodontitis: (a) Forest plot of sensitivity, (b) Forest plot of specificity, (c) Forest plot of diagnostic odds ratio (DOR), (d) Baujat plot, (e) Deeks's funnel plot.**

**
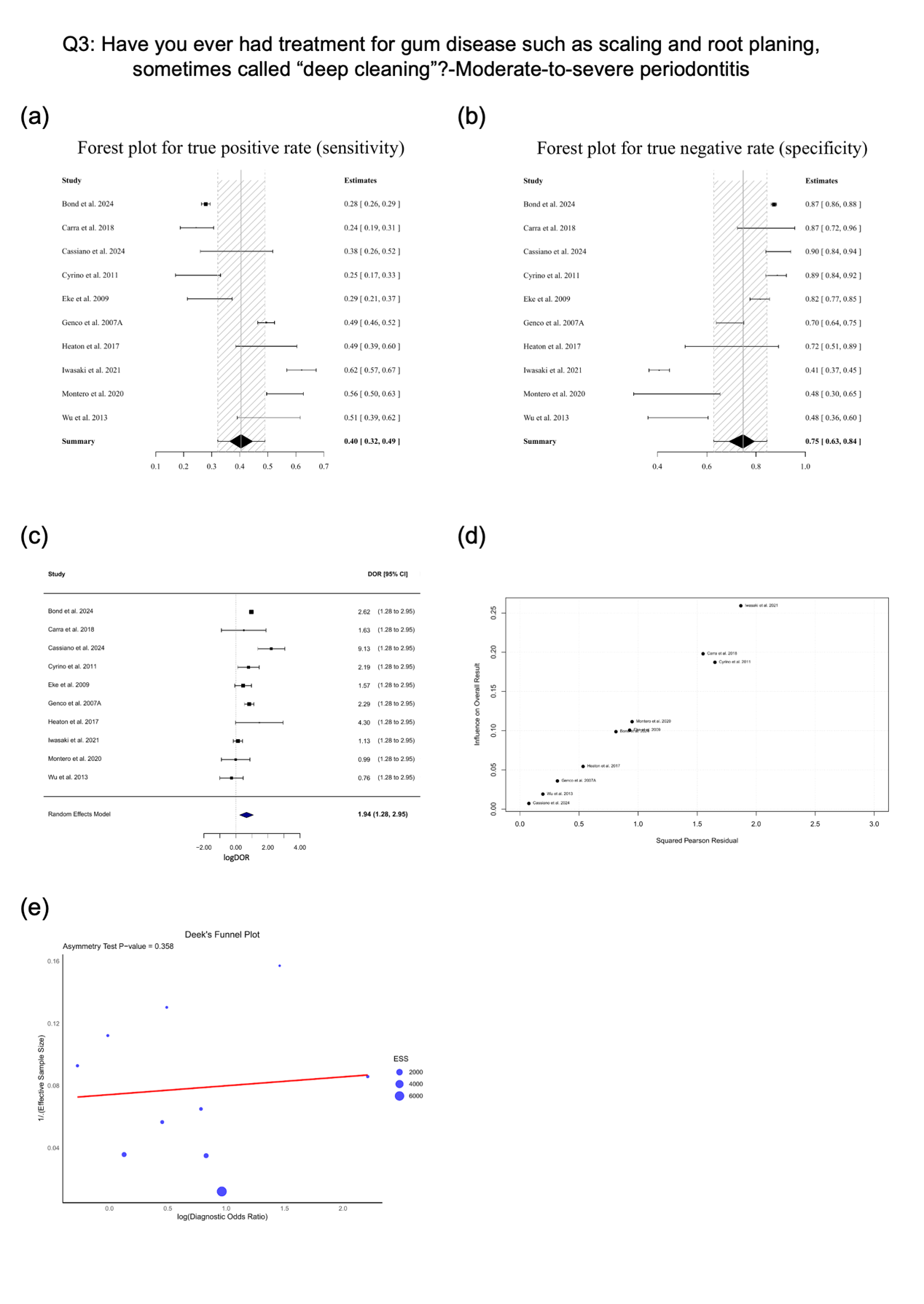
**

**Figure S10. Q3 "Have you ever had treatment for gum disease such as scaling and root planing, sometimes called ‘deep cleaning’?" for detecting severe periodontitis: (a) Forest plot of sensitivity, (b) Forest plot of specificity, (c) Forest plot of diagnostic odds ratio (DOR), (d) Baujat plot, (e) Deeks's funnel plot.**

**
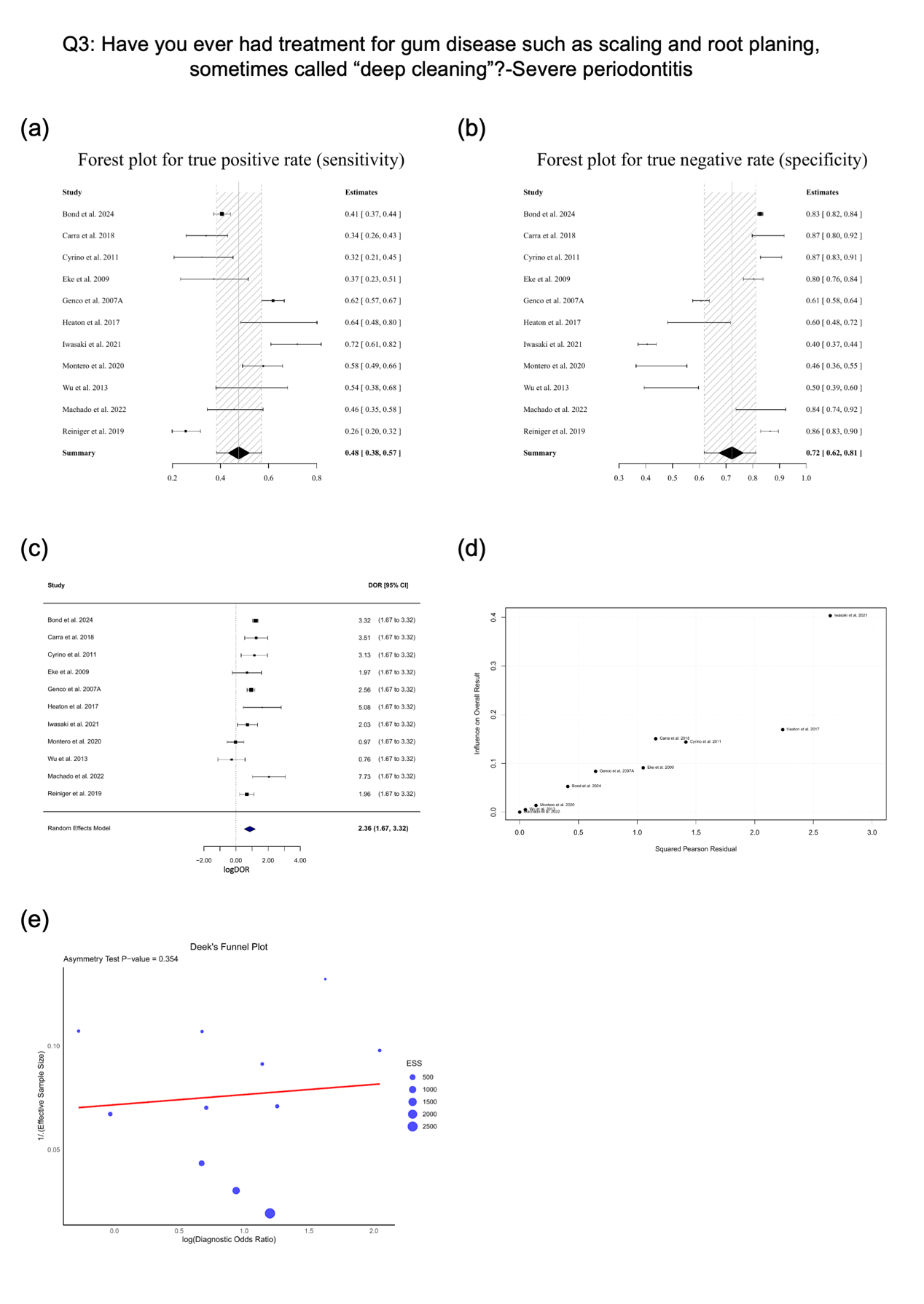
**

**Figure S11. Q4 "Have you ever had any teeth become loose on their own, without an injury?" for detecting total periodontitis: (a) Forest plot of sensitivity, (b) Forest plot of specificity, (c) Forest plot of diagnostic odds ratio (DOR), (d) Baujat plot, (e) Deeks's funnel plot.**

**
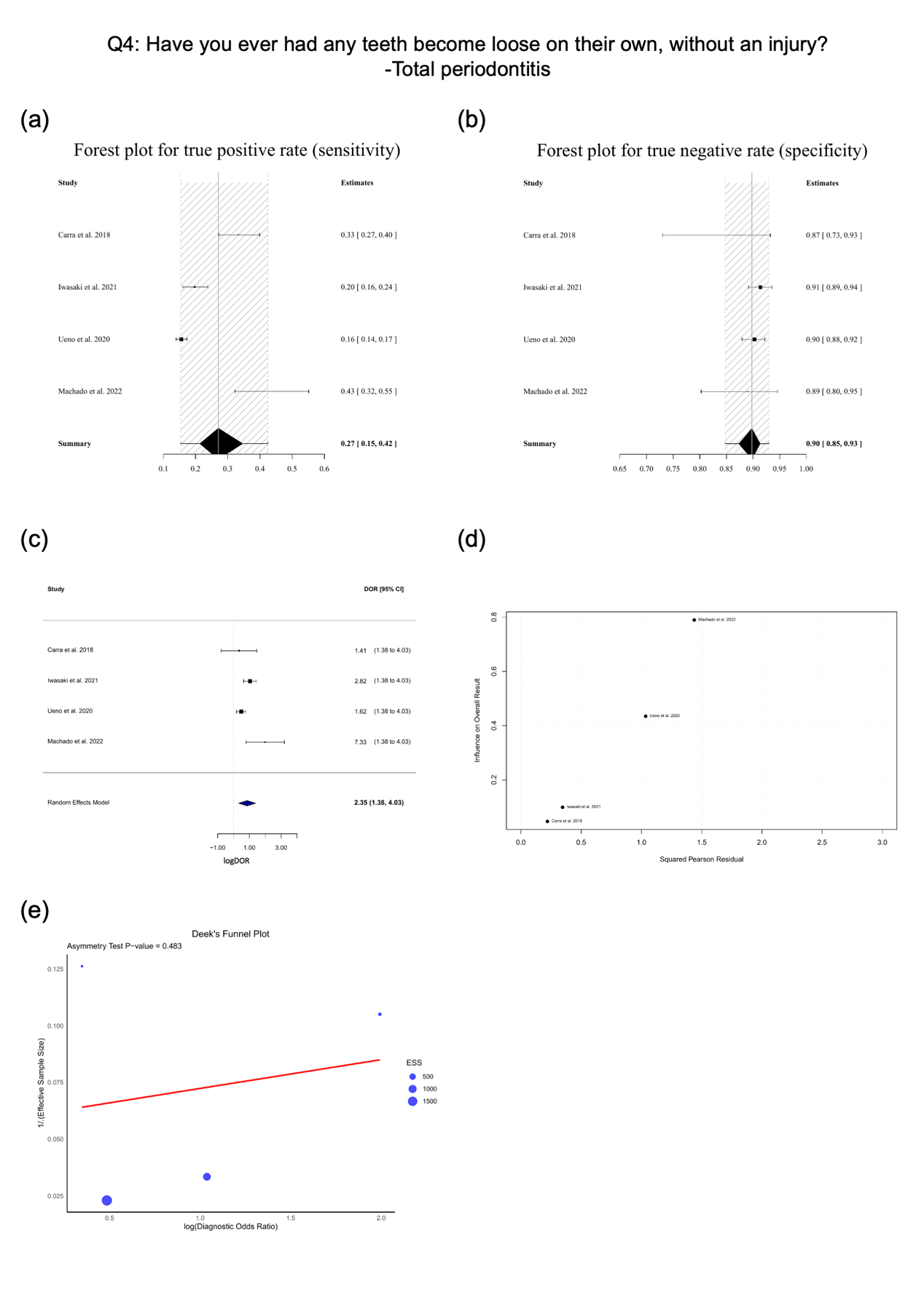
**

**Figure S12. Q4 "Have you ever had any teeth become loose on their own, without an injury?" for detecting moderate-to-severe periodontitis: (a) Forest plot of sensitivity, (b) Forest plot of specificity, (c) Forest plot of diagnostic odds ratio (DOR), (d) Baujat plot, (e) Deeks's funnel plot.**

**
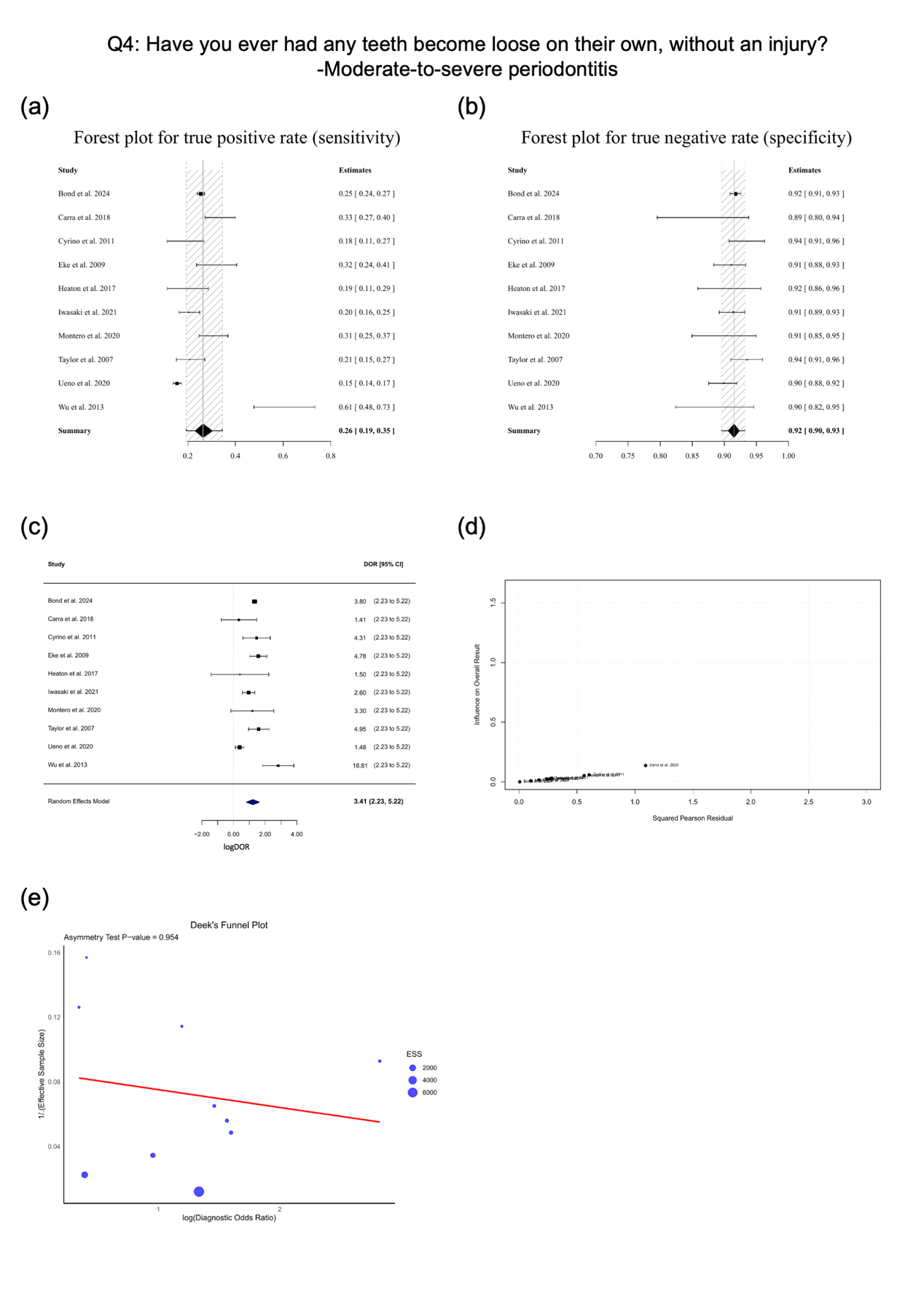
**

**Figure S13. Q4 "Have you ever had any teeth become loose on their own, without an injury?" for detecting severe periodontitis: (a) Forest plot of sensitivity, (b) Forest plot of specificity, (c) Forest plot of diagnostic odds ratio (DOR), (d) Baujat plot, (e) Deeks's funnel plot.**

**
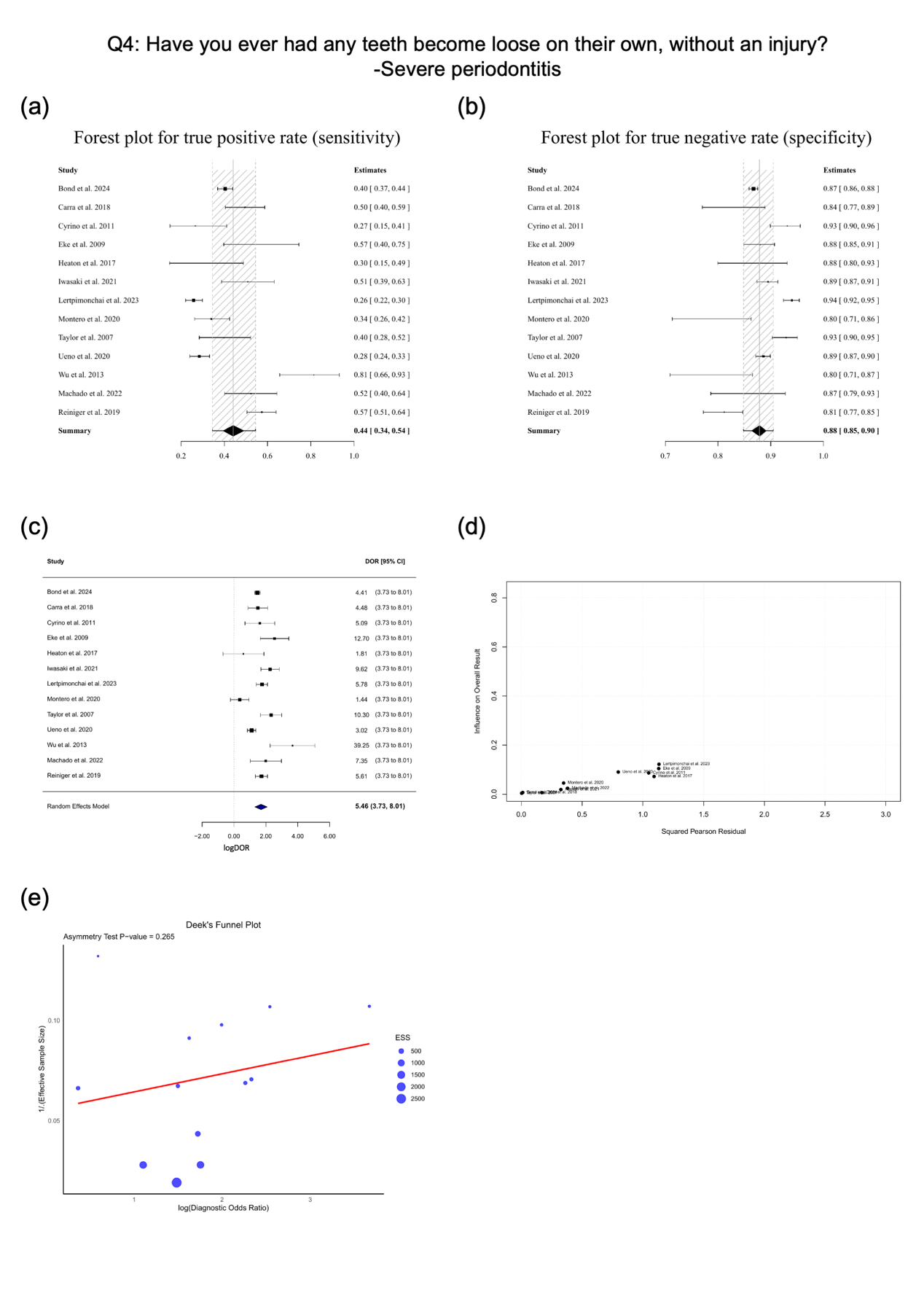
**

**Figure S14. Q5 "Have you ever been told by a dental professional that you lost bone around your teeth?" for detecting total periodontitis: (a) Forest plot of sensitivity, (b) Forest plot of specificity, (c) Forest plot of diagnostic odds ratio (DOR), (d) Baujat plot, (e) Deeks's funnel plot.**

**
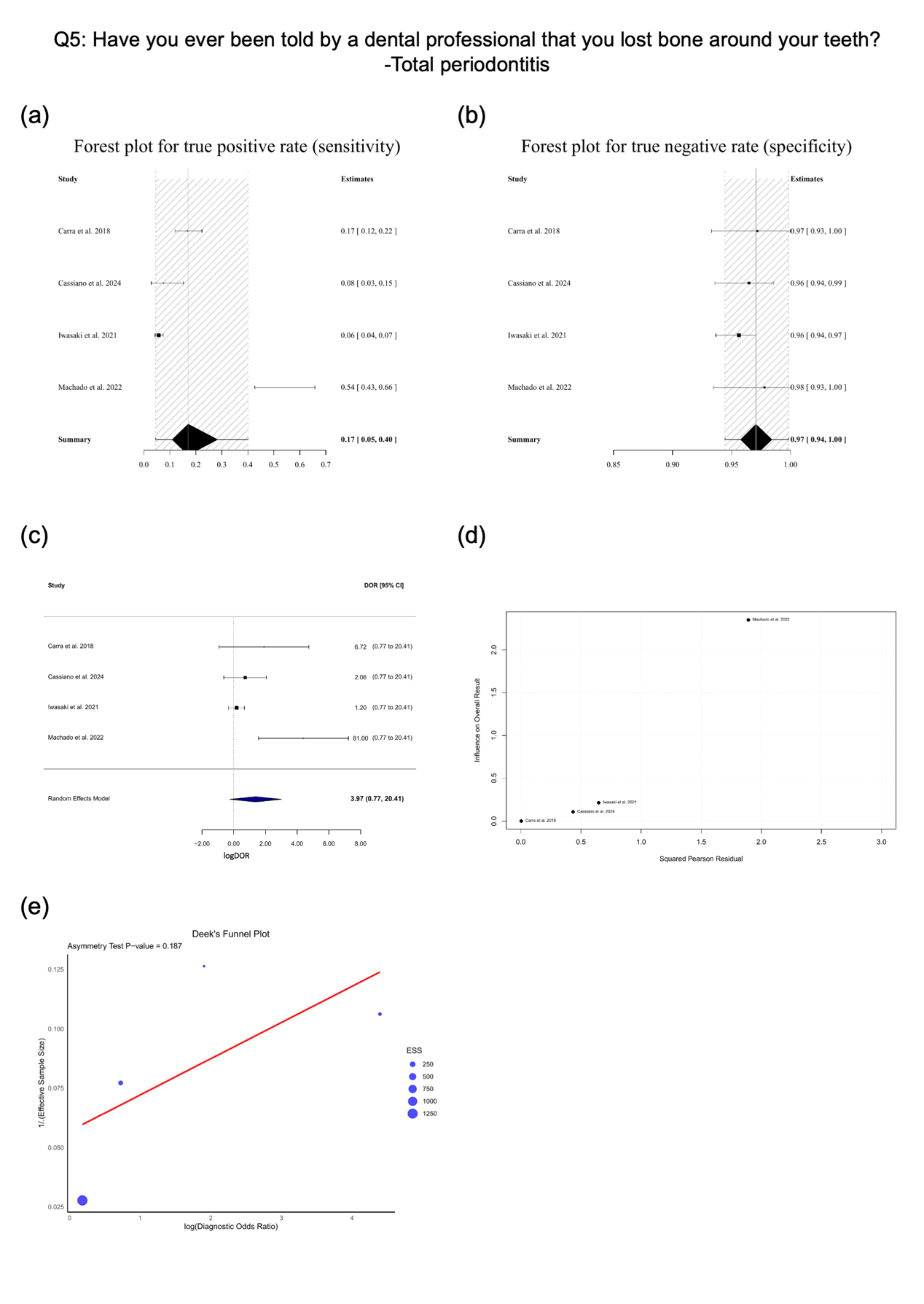
**

**Figure S15. Q5 "Have you ever been told by a dental professional that you lost bone around your teeth?" for detecting moderate-to-severe periodontitis: (a) Forest plot of sensitivity, (b) Forest plot of specificity, (c) Forest plot of diagnostic odds ratio (DOR), (d) Baujat plot, (e) Deeks's funnel plot.**

**
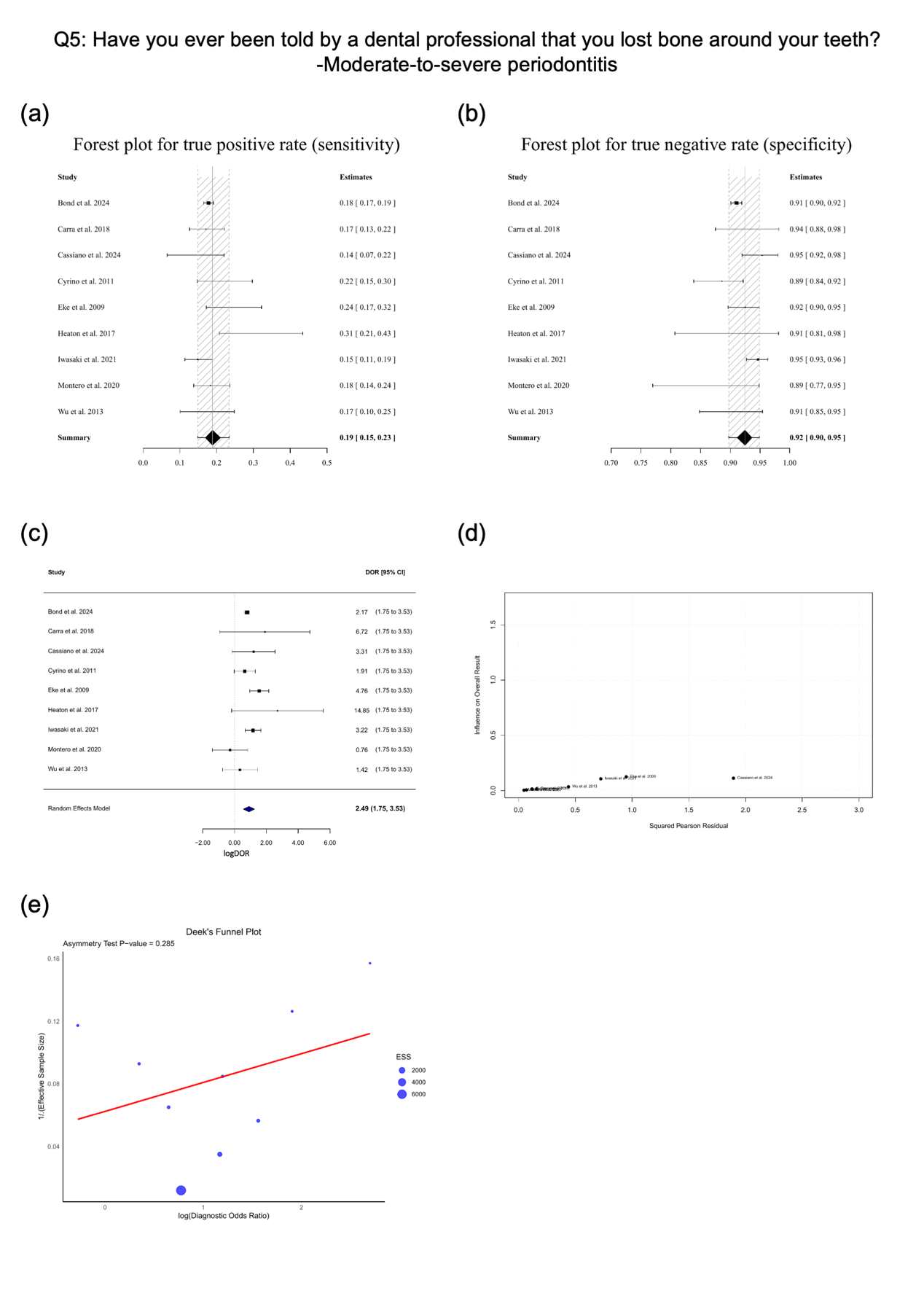
**

**Figure S16. Q5 "Have you ever been told by a dental professional that you lost bone around your teeth?" for detecting severe periodontitis: (a) Forest plot of sensitivity, (b) Forest plot of specificity, (c) Forest plot of diagnostic odds ratio (DOR), (d) Baujat plot, (e) Deeks's funnel plot.**

**
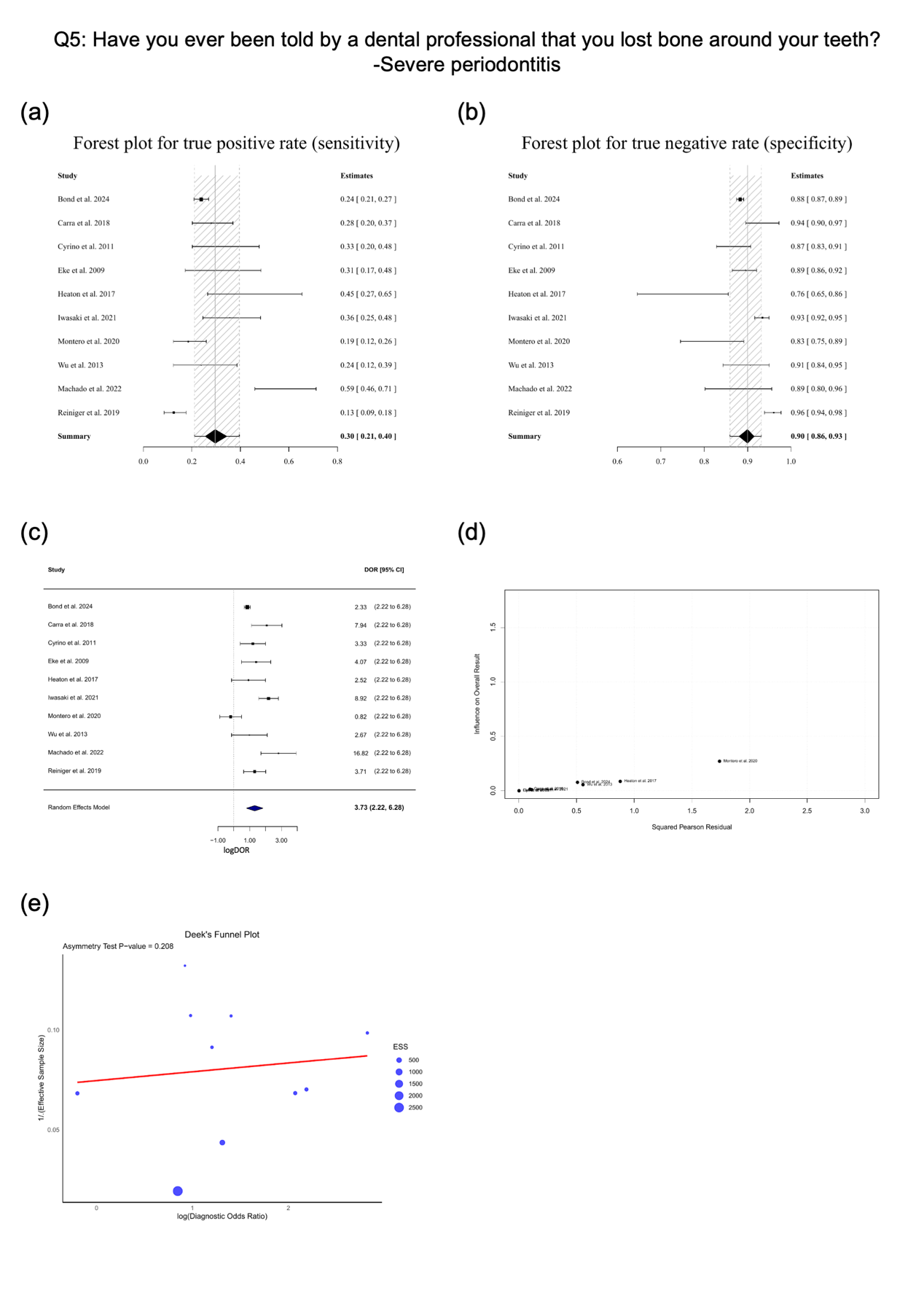
**

**Figure S17. Q6 "During the past 3 months, have you noticed a tooth that doesn’t look right?" for detecting total periodontitis: (a) Forest plot of sensitivity, (b) Forest plot of specificity, (c) Forest plot of diagnostic odds ratio (DOR), (d) Baujat plot, (e) Deeks's funnel plot.**

**
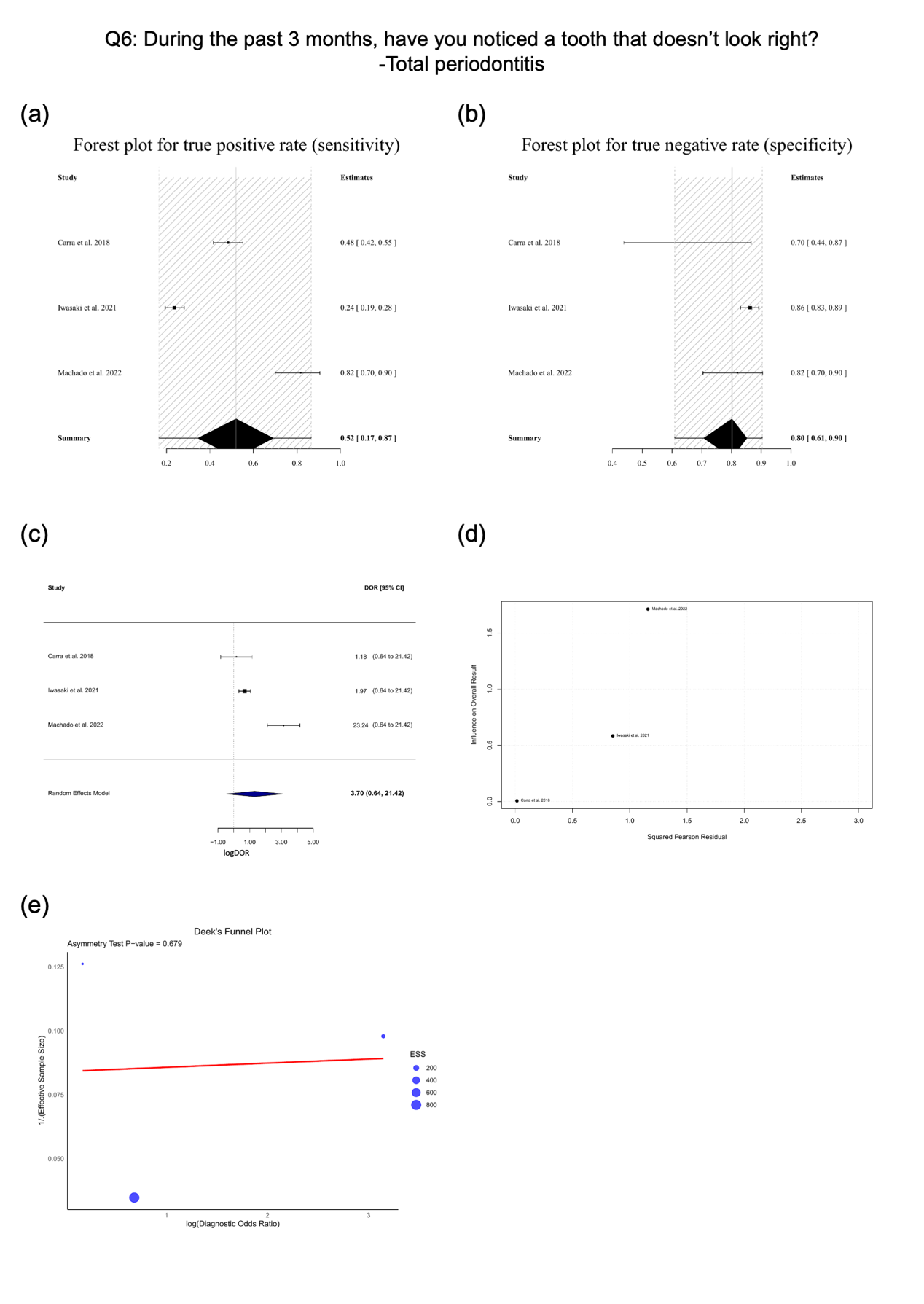
**

**Figure S18. Q6 "During the past 3 months, have you noticed a tooth that doesn’t look right?" for detecting moderate-to-severe periodontitis: (a) Forest plot of sensitivity, (b) Forest plot of specificity, (c) Forest plot of diagnostic odds ratio (DOR), (d) Baujat plot, (e) Deeks's funnel plot.**

**
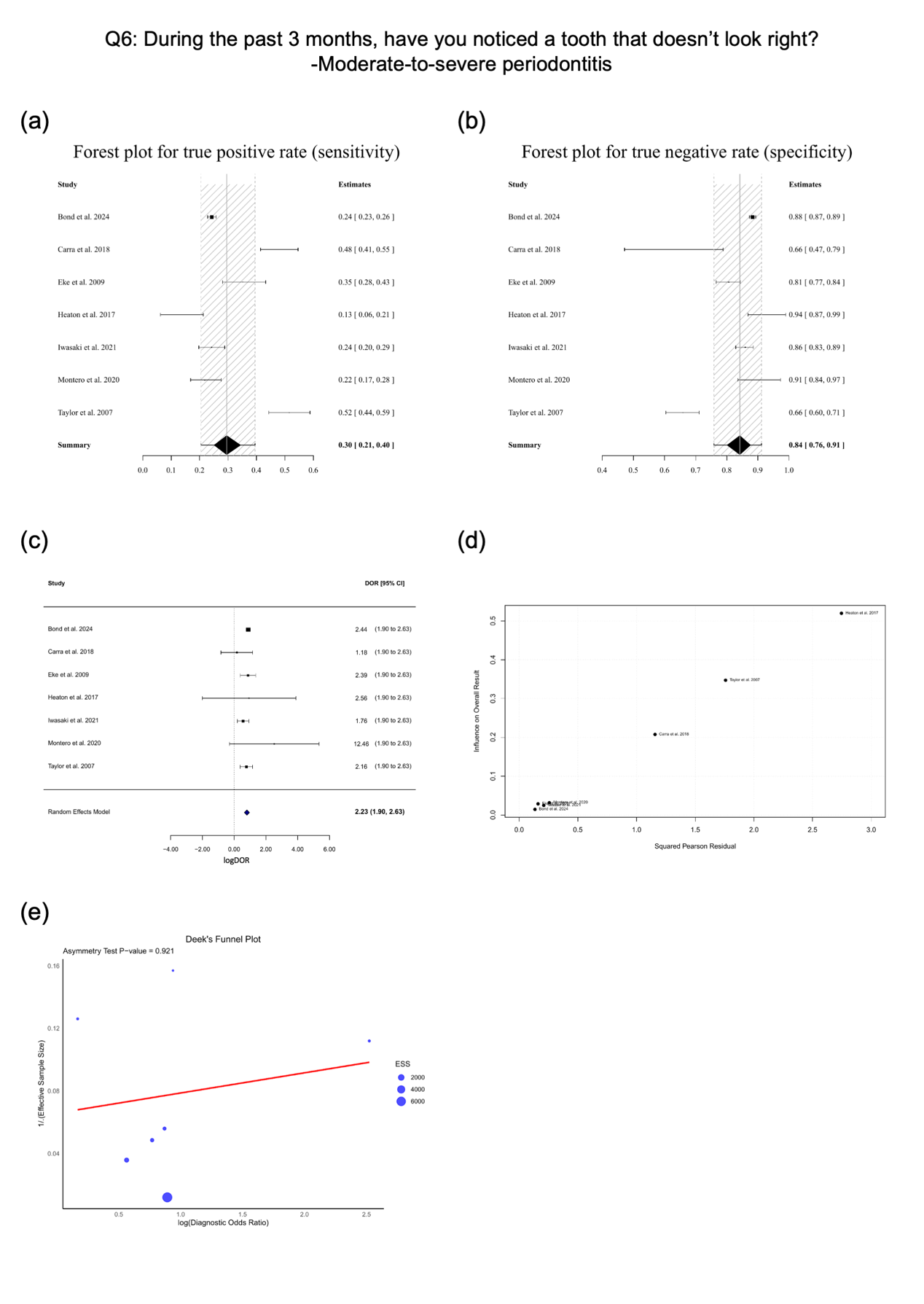
**

**Figure S19. Q6 "During the past 3 months, have you noticed a tooth that doesn’t look right?" for detecting severe periodontitis: (a) Forest plot of sensitivity, (b) Forest plot of specificity, (c) Forest plot of diagnostic odds ratio (DOR), (d) Baujat plot, (e) Deeks's funnel plot.**

**
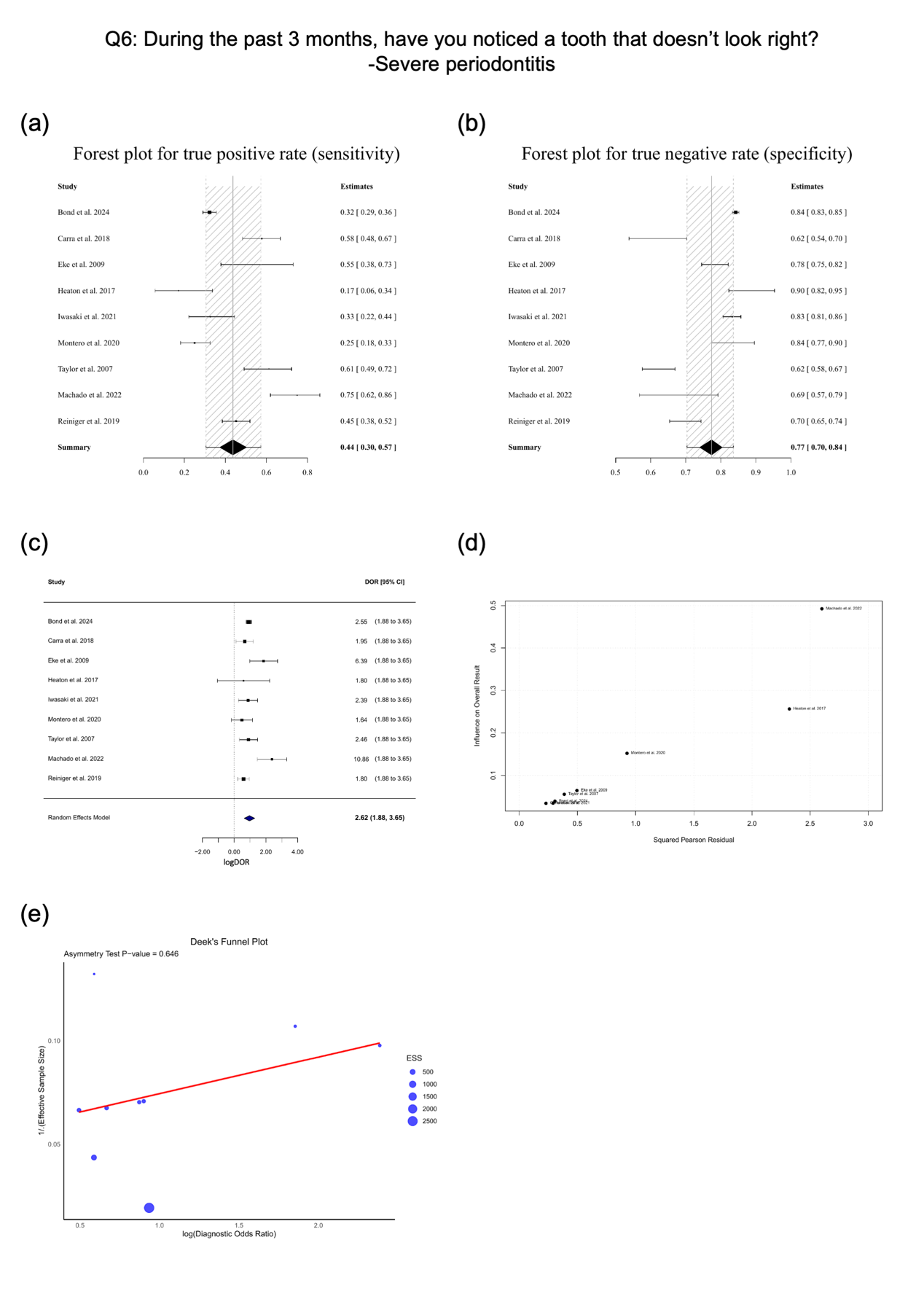
**

**Figure S20. Q7 "Do you use dental floss/other device to clean your teeth?" for detecting total periodontitis: (a) Forest plot of sensitivity, (b) Forest plot of specificity, (c) Forest plot of diagnostic odds ratio (DOR), (d) Baujat plot, (e) Deeks's funnel plot.**

**
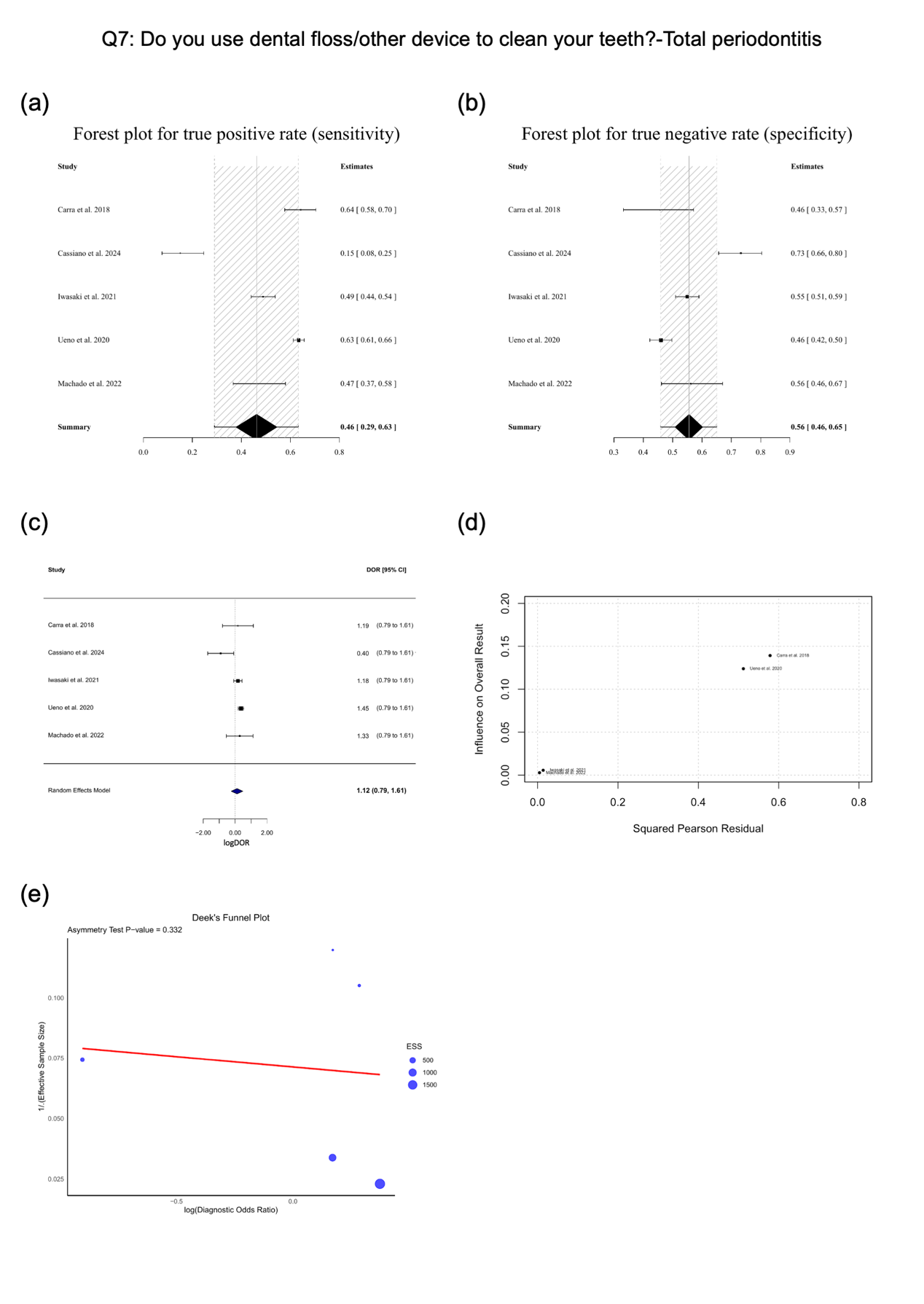
**

**Figure S21. Q7 "Do you use dental floss/other device to clean your teeth?" for detecting moderate-to-severe periodontitis: (a) Forest plot of sensitivity, (b) Forest plot of specificity, (c) Forest plot of diagnostic odds ratio (DOR), (d) Baujat plot, (e) Deeks's funnel plot.**

**
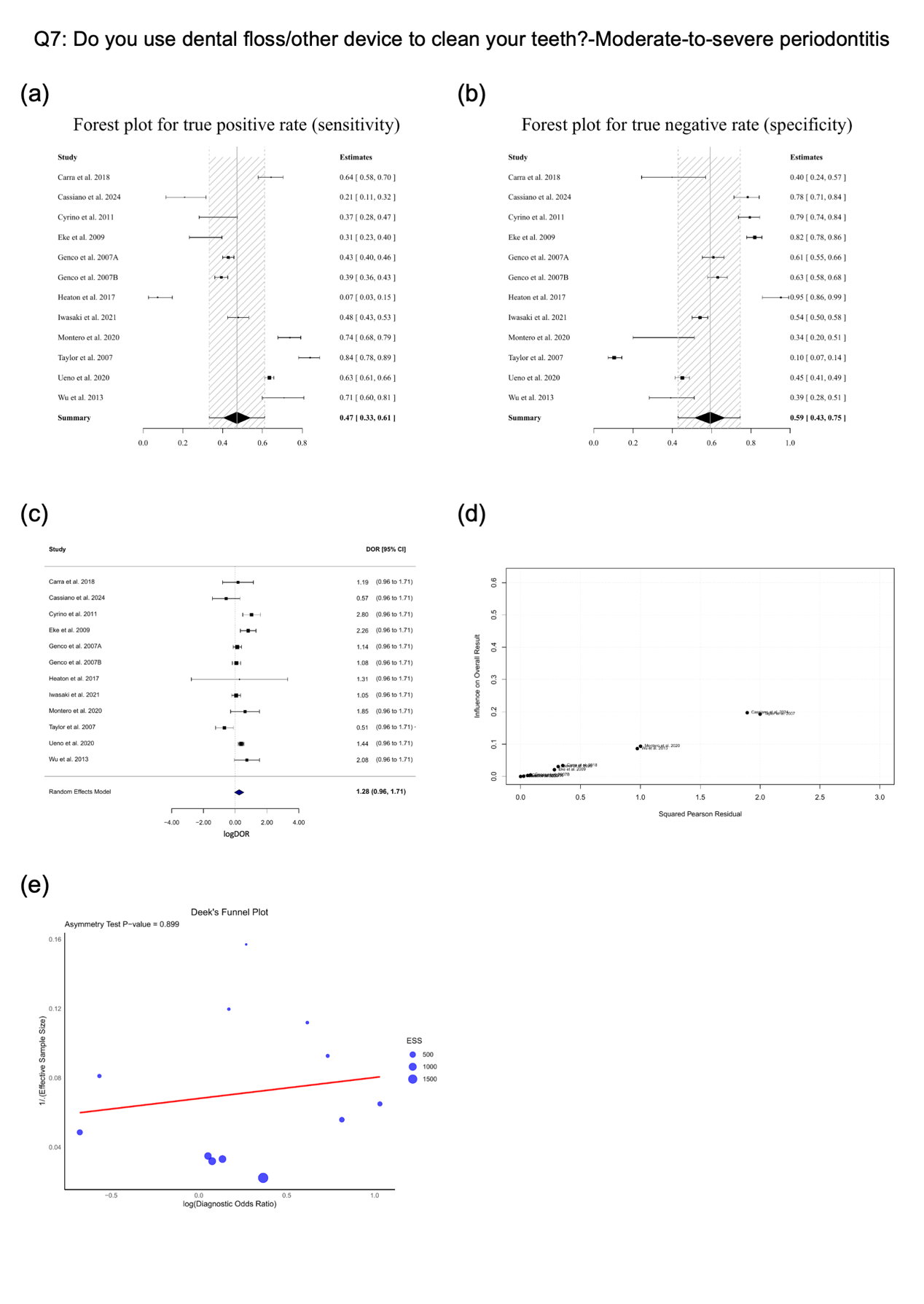
**

**Figure S22. Q7 "Do you use dental floss/other device to clean your teeth?" for detecting severe periodontitis: (a) Forest plot of sensitivity, (b) Forest plot of specificity, (c) Forest plot of diagnostic odds ratio (DOR), (d) Baujat plot, (e) Deeks's funnel plot.**

**
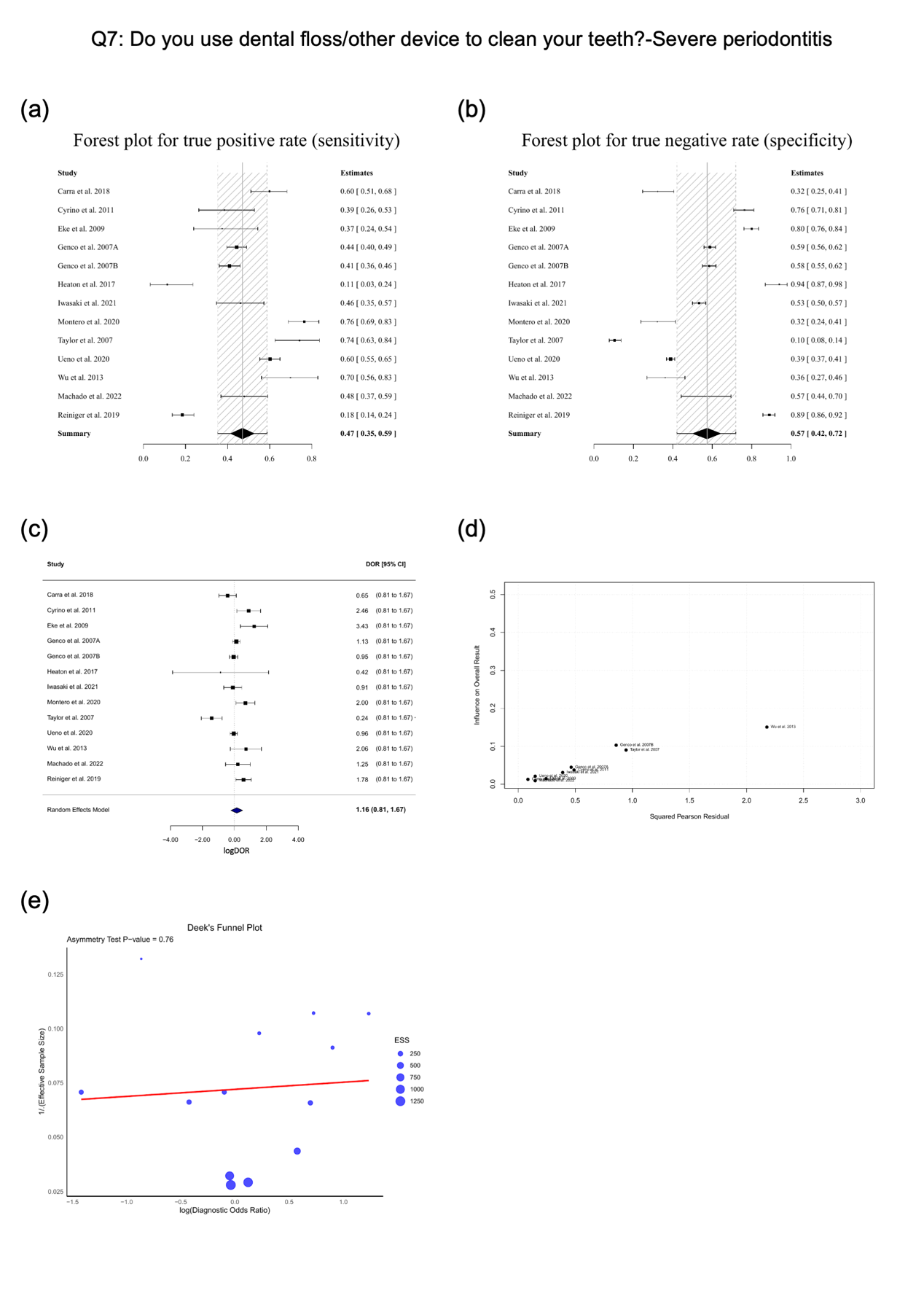
**

**Figure S23. Q8 "Do you use mouthwash or other dental rinse product to treat dental disease or dental problems?" for detecting total periodontitis: (a) Forest plot of sensitivity, (b) Forest plot of specificity, (c) Forest plot of diagnostic odds ratio (DOR), (d) Baujat plot, (e) Deeks's funnel plot.**

**
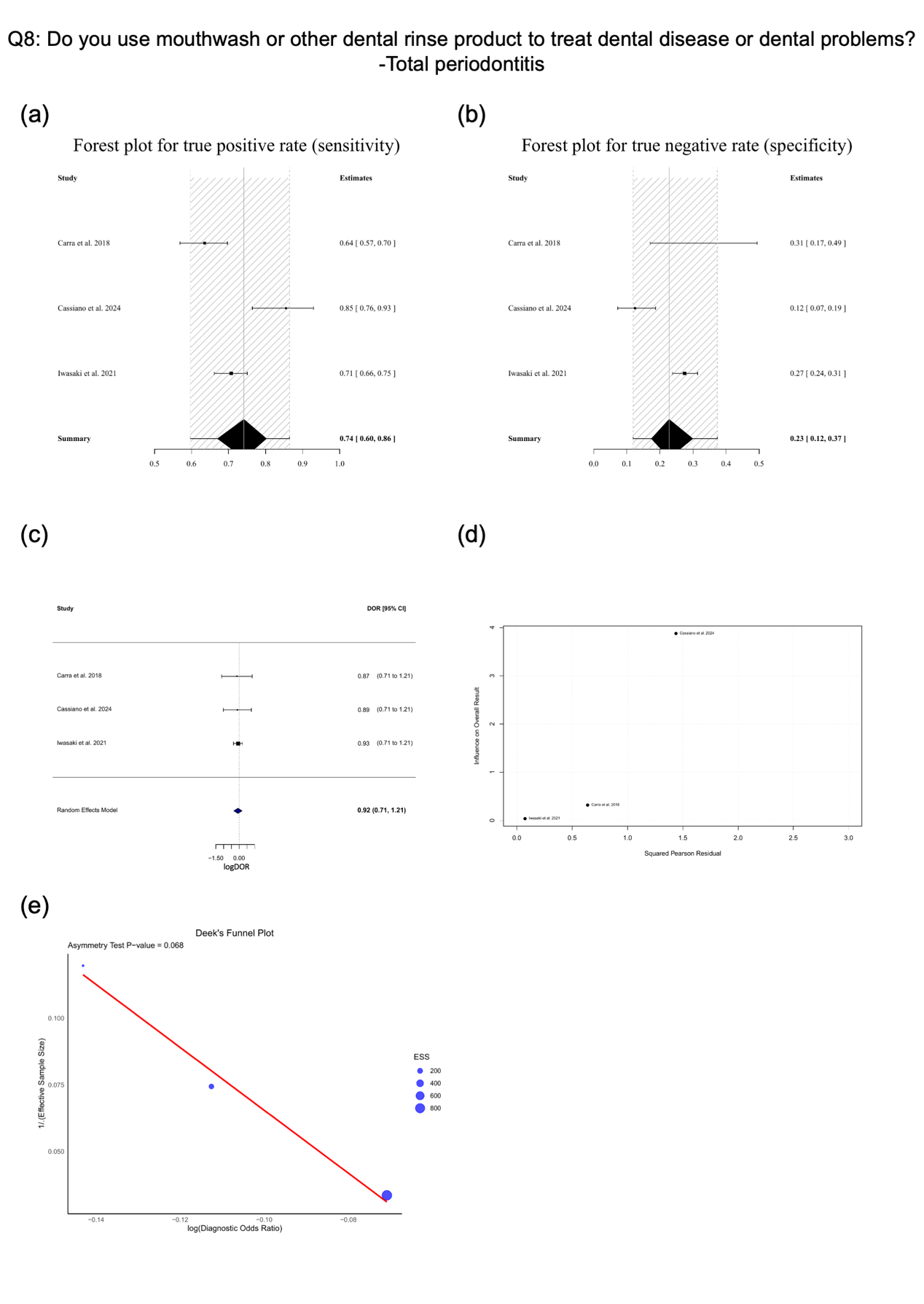
**

**Figure S24. Q8 "Do you use mouthwash or other dental rinse product to treat dental disease or dental problems?" for detecting moderate-to-severe periodontitis: (a) Forest plot of sensitivity, (b) Forest plot of specificity, (c) Forest plot of diagnostic odds ratio (DOR), (d) Baujat plot, (e) Deeks's funnel plot.**

**
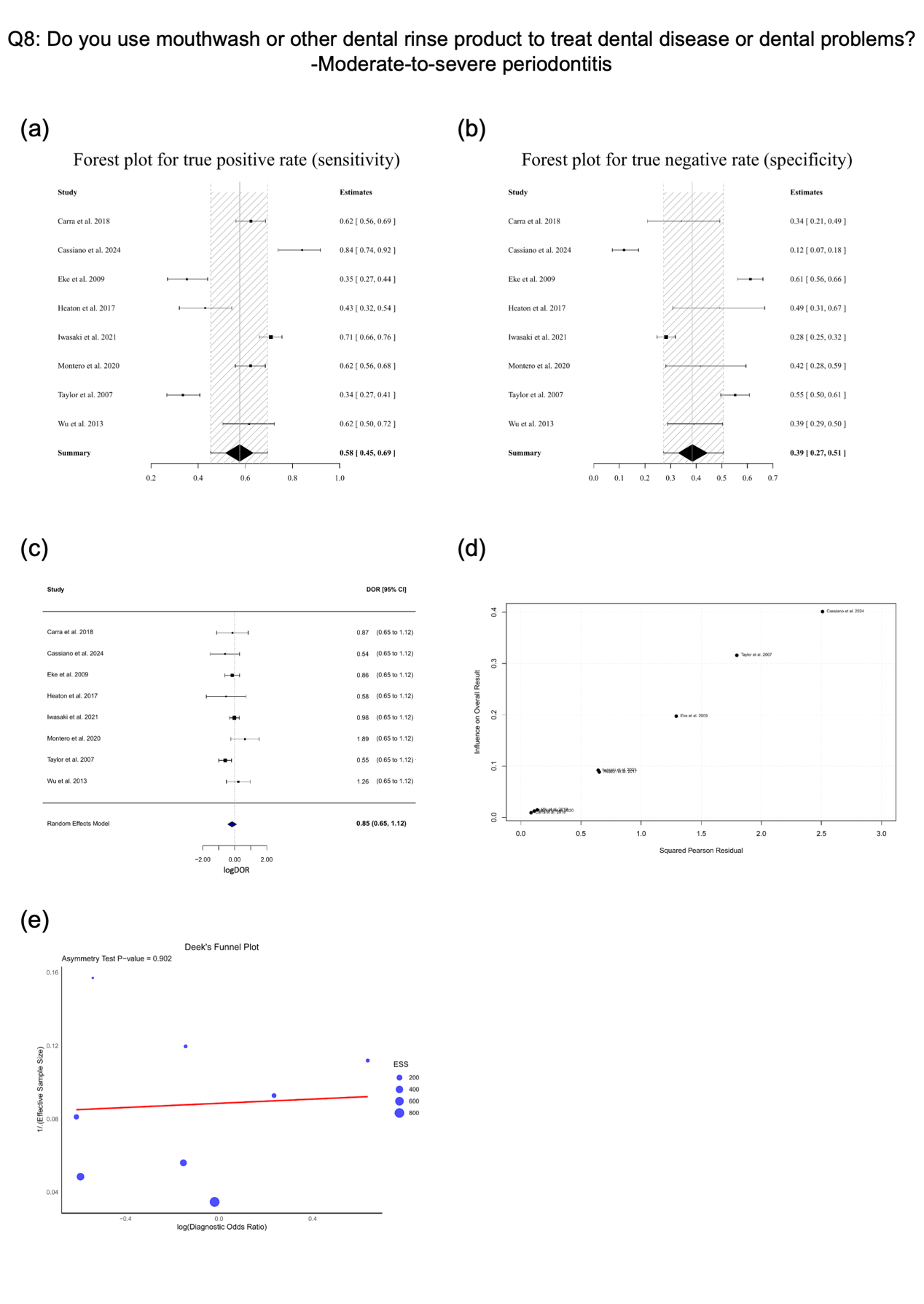
**

**Figure S25. Q8 "Do you use mouthwash or other dental rinse product to treat dental disease or dental problems?" for detecting severe periodontitis: (a) Forest plot of sensitivity, (b) Forest plot of specificity, (c) Forest plot of diagnostic odds ratio (DOR), (d) Baujat plot, (e) Deeks's funnel plot.**

**
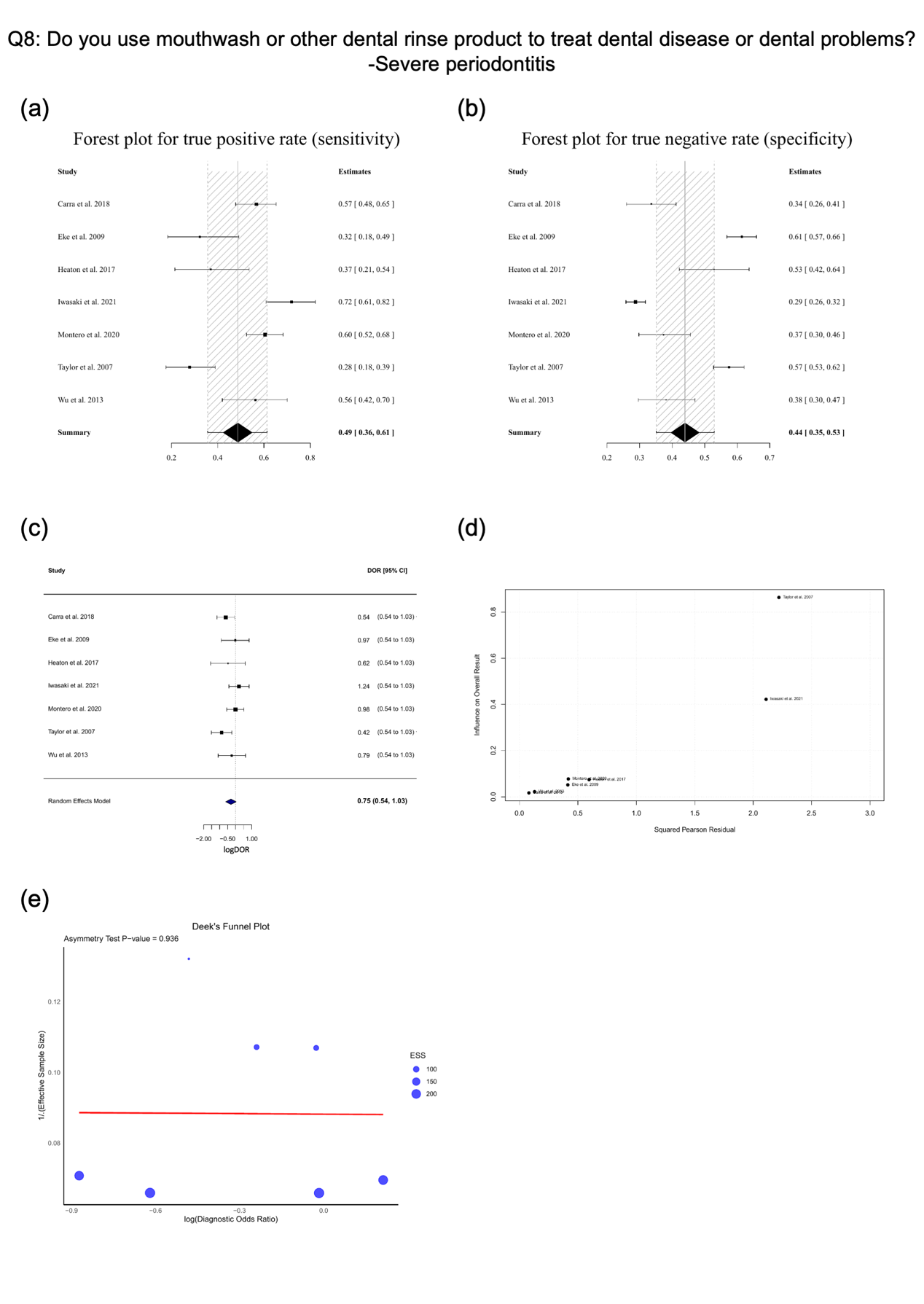
**

**Figure S26. Q9 "Do you have bleeding gums?" for detecting total periodontitis: (a) Forest plot of sensitivity, (b) Forest plot of specificity, (c) Forest plot of diagnostic odds ratio (DOR), (d) Baujat plot, (e) Deeks's funnel plot, (f) HSROC curve.**

**
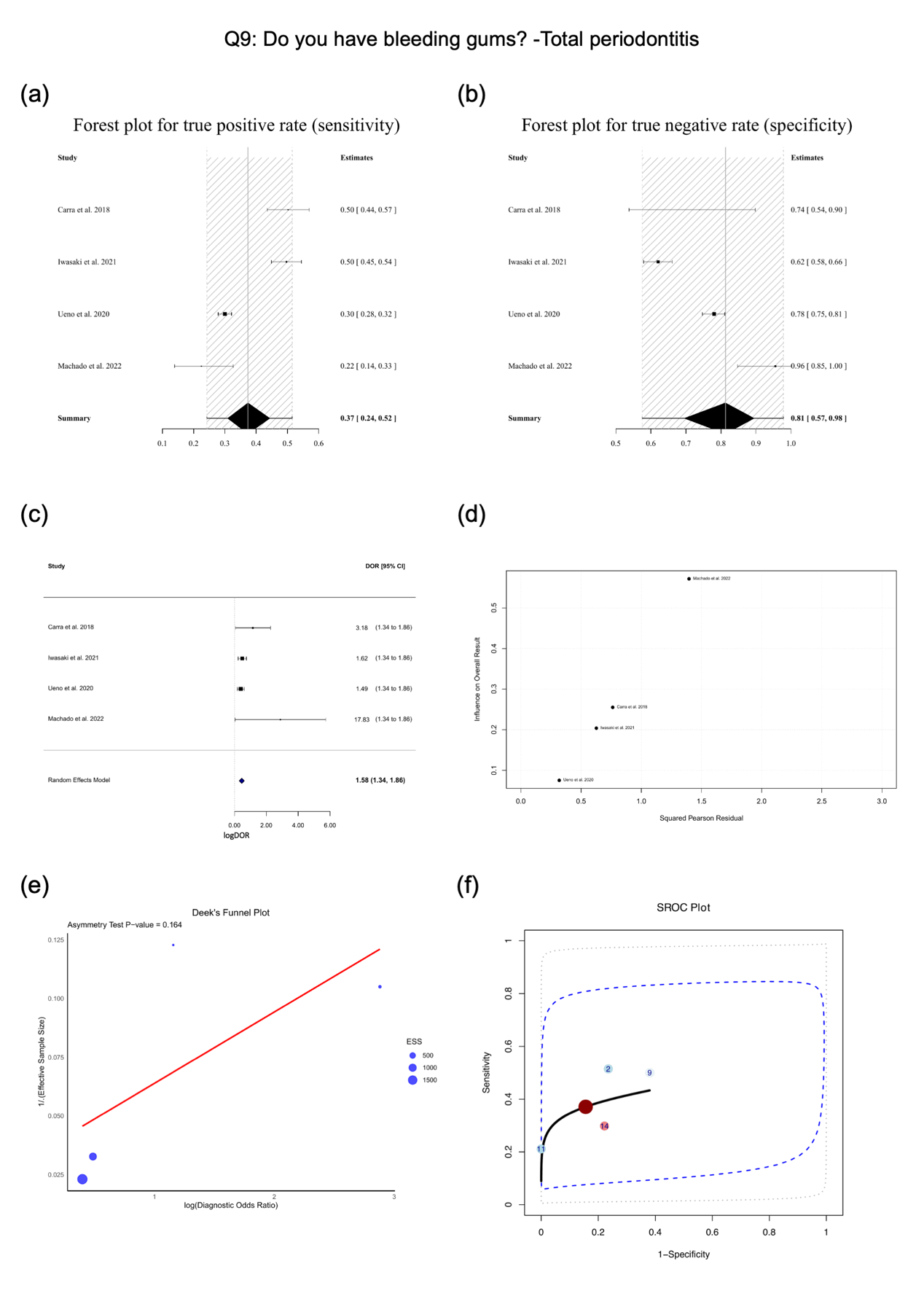
**

**Figure S27. Q9 "Do you have bleeding gums?" for detecting moderate-to-severe periodontitis: (a) Forest plot of sensitivity, (b) Forest plot of specificity, (c) Forest plot of diagnostic odds ratio (DOR), (d) Baujat plot, (e) Deeks's funnel plot, (f) HSROC curve.**

**
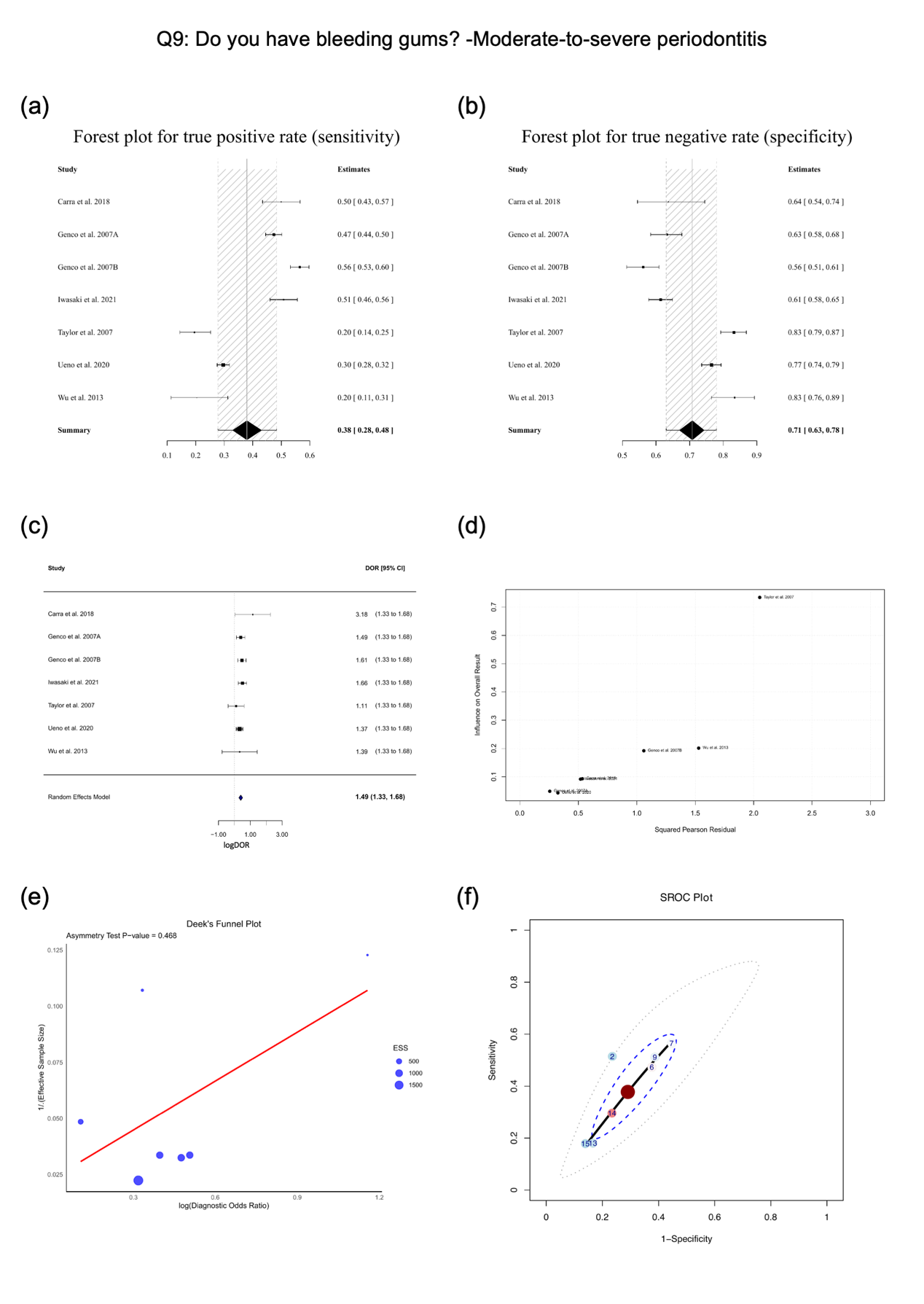
**

**Figure S28. Q9 "Do you have bleeding gums?" for detecting severe periodontitis: (a) Forest plot of sensitivity, (b) Forest plot of specificity, (c) Forest plot of diagnostic odds ratio (DOR), (d) Baujat plot, (e) Deeks's funnel plot, (f) HSROC curve.**

**
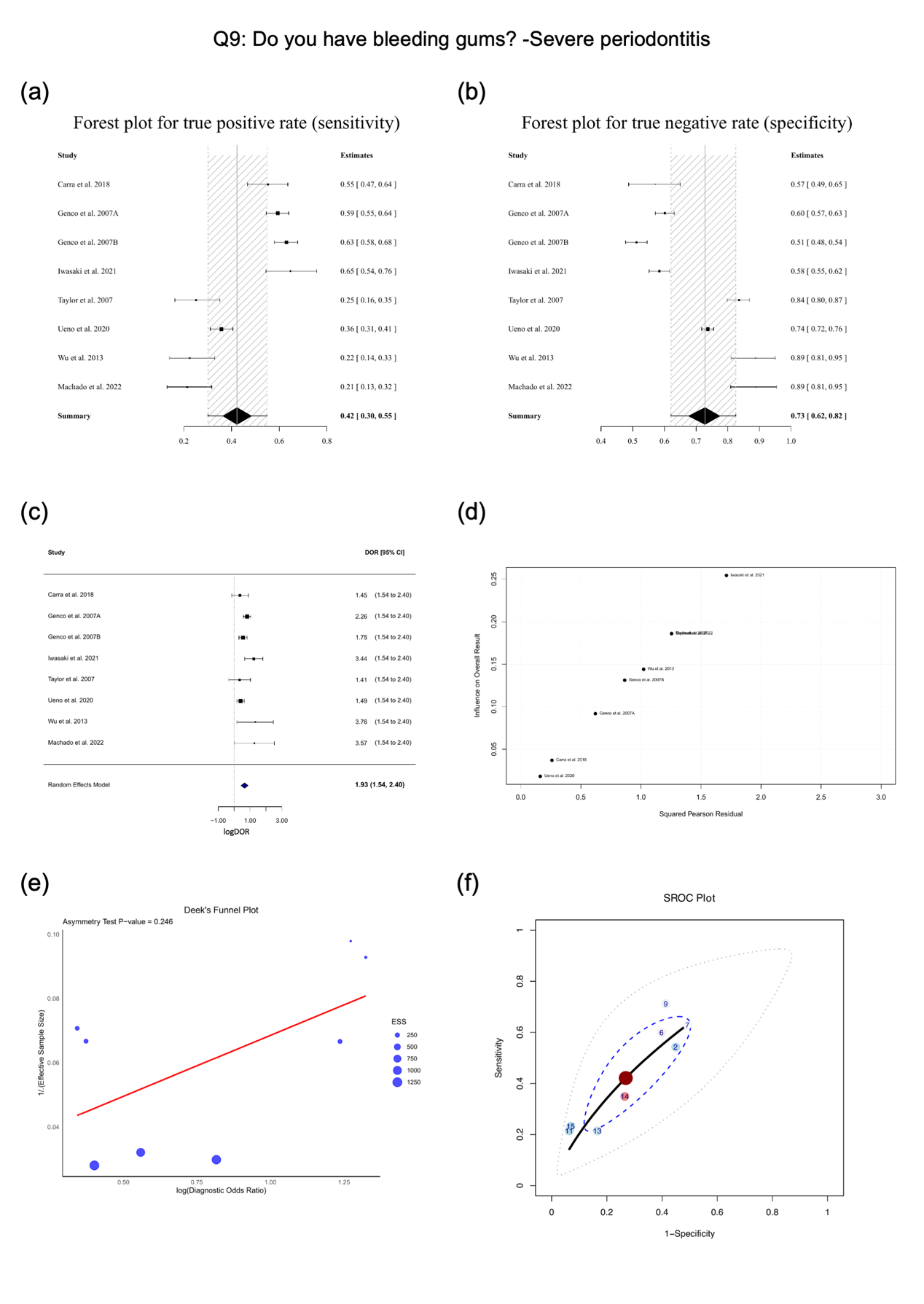
**

**Figure S29. Q10 "Do you have tooth loss?" for detecting moderate-to-severe periodontitis: (a) Forest plot of sensitivity, (b) Forest plot of specificity, (c) Forest plot of diagnostic odds ratio (DOR), (d) Baujat plot, (e) Deeks's funnel plot, (f) HSROC curve.**

**
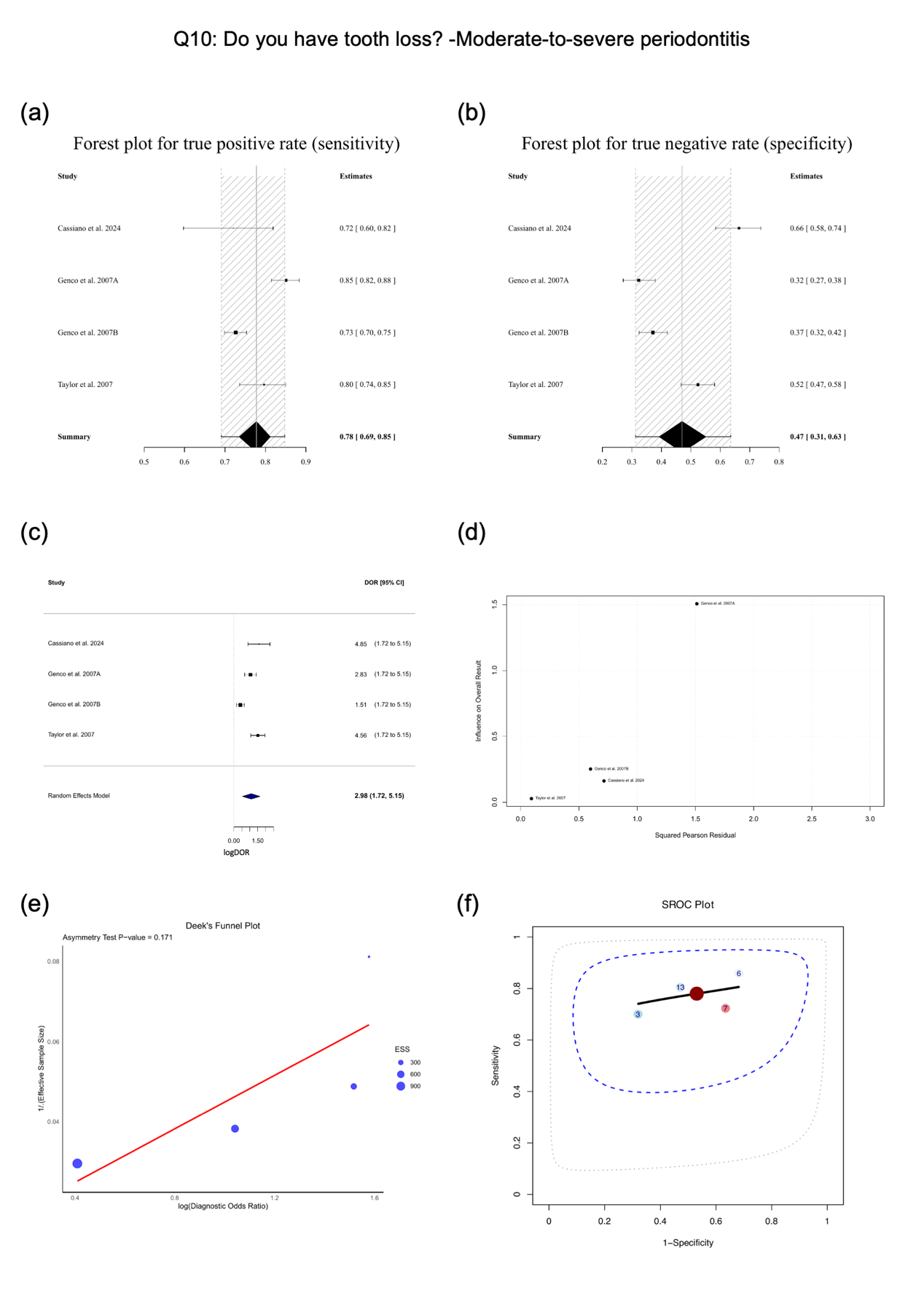
**

**Figure S30. Q10 "Do you have tooth loss?" for detecting severe periodontitis: (a) Forest plot of sensitivity, (b) Forest plot of specificity, (c) Forest plot of diagnostic odds ratio (DOR), (d) Baujat plot, (e) Deeks's funnel plot, (f) HSROC curve.**

**
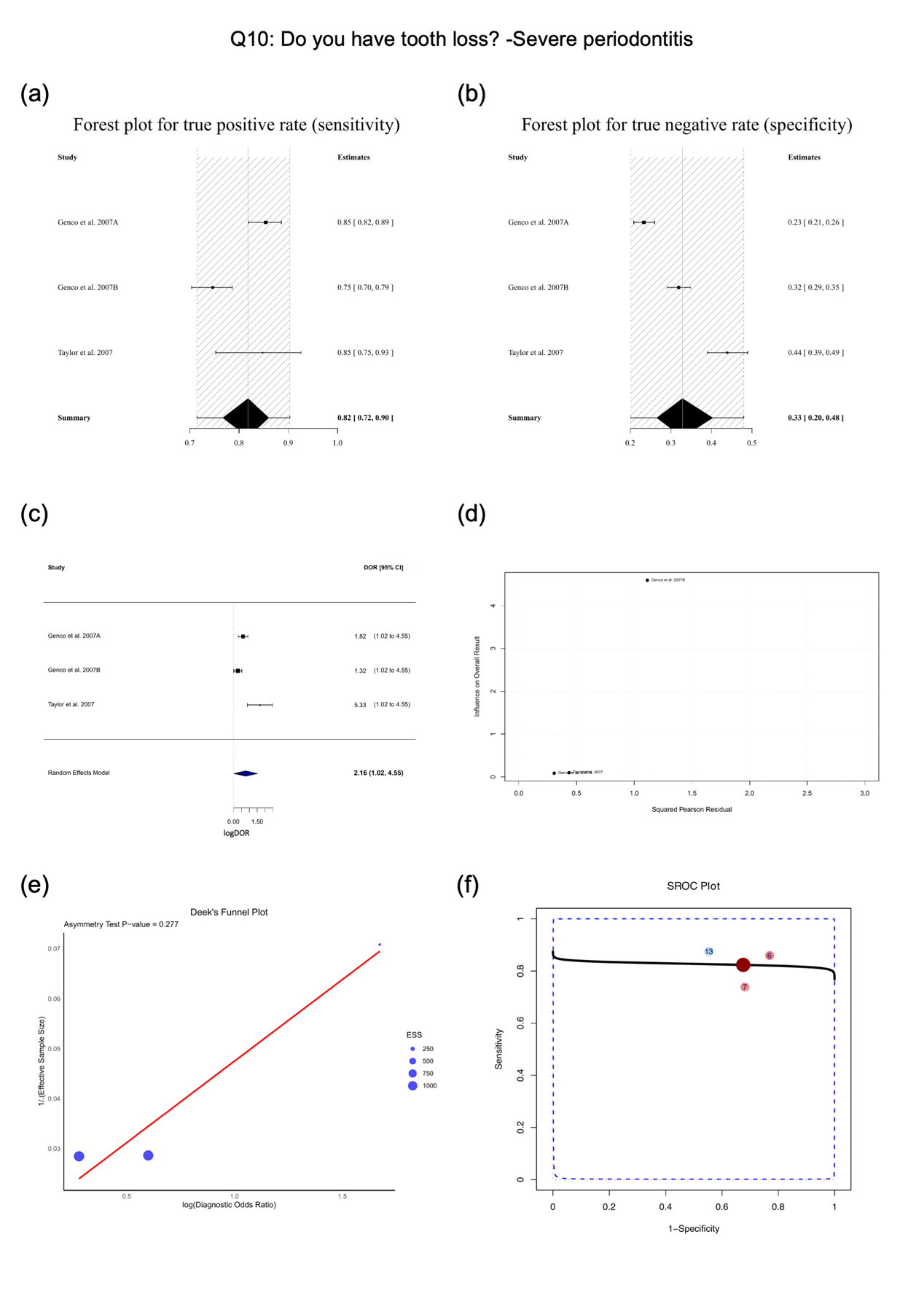
**

**Figure S31. Q11 "Do you have tooth loss because of mobility?" for detecting severe periodontitis: (a) Forest plot of sensitivity, (b) Forest plot of specificity, (c) Forest plot of diagnostic odds ratio (DOR), (d) Baujat plot, (e) Deeks's funnel plot, (f) HSROC curve.**

**
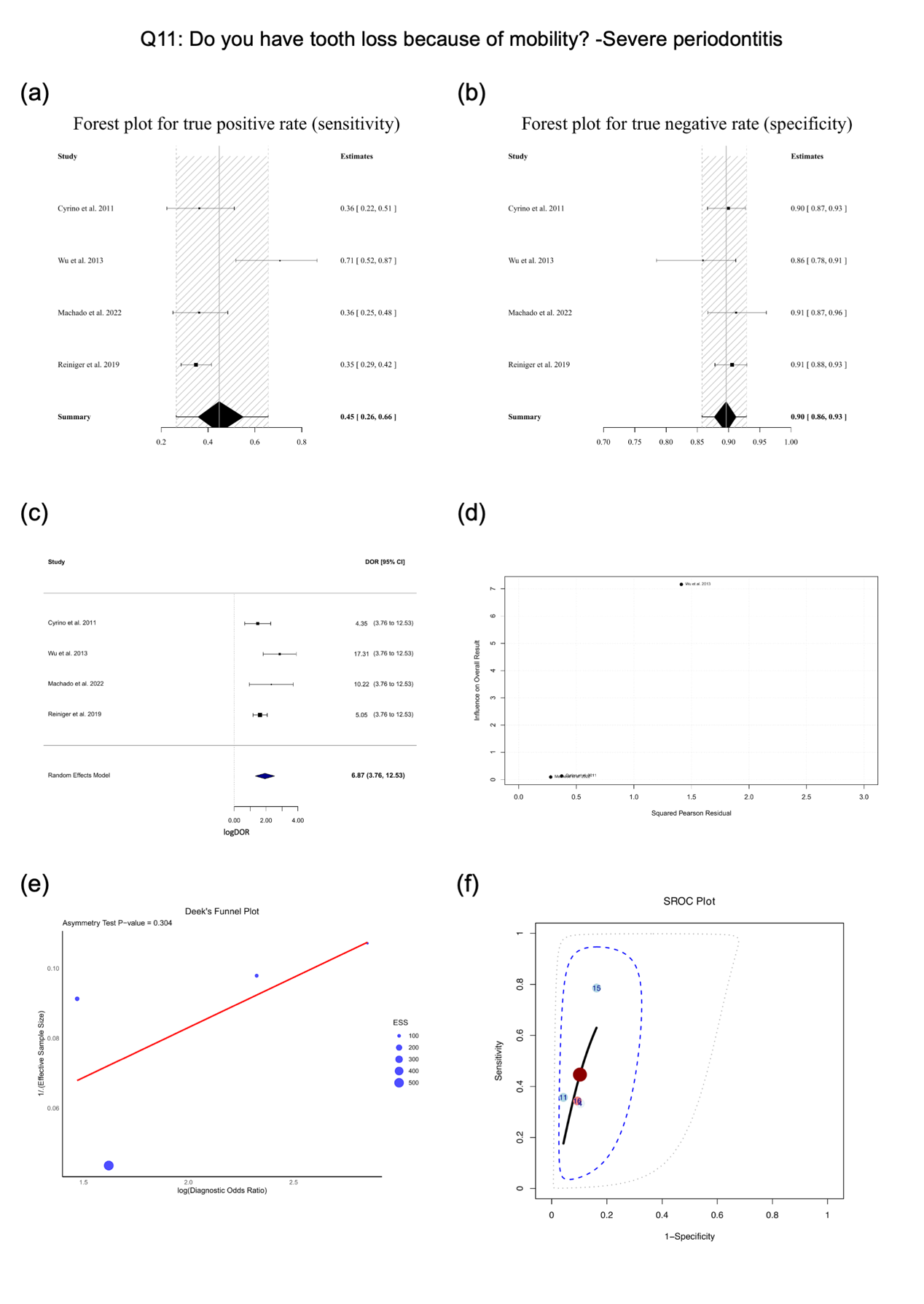
**

**Figure S32. Q12 "Are you satisfied with your breath/taste?" for detecting moderate-to-severe periodontitis: (a) Forest plot of sensitivity, (b) Forest plot of specificity, (c) Forest plot of diagnostic odds ratio (DOR), (d) Baujat plot, (e) Deeks's funnel plot, (f) HSROC curve.**

**
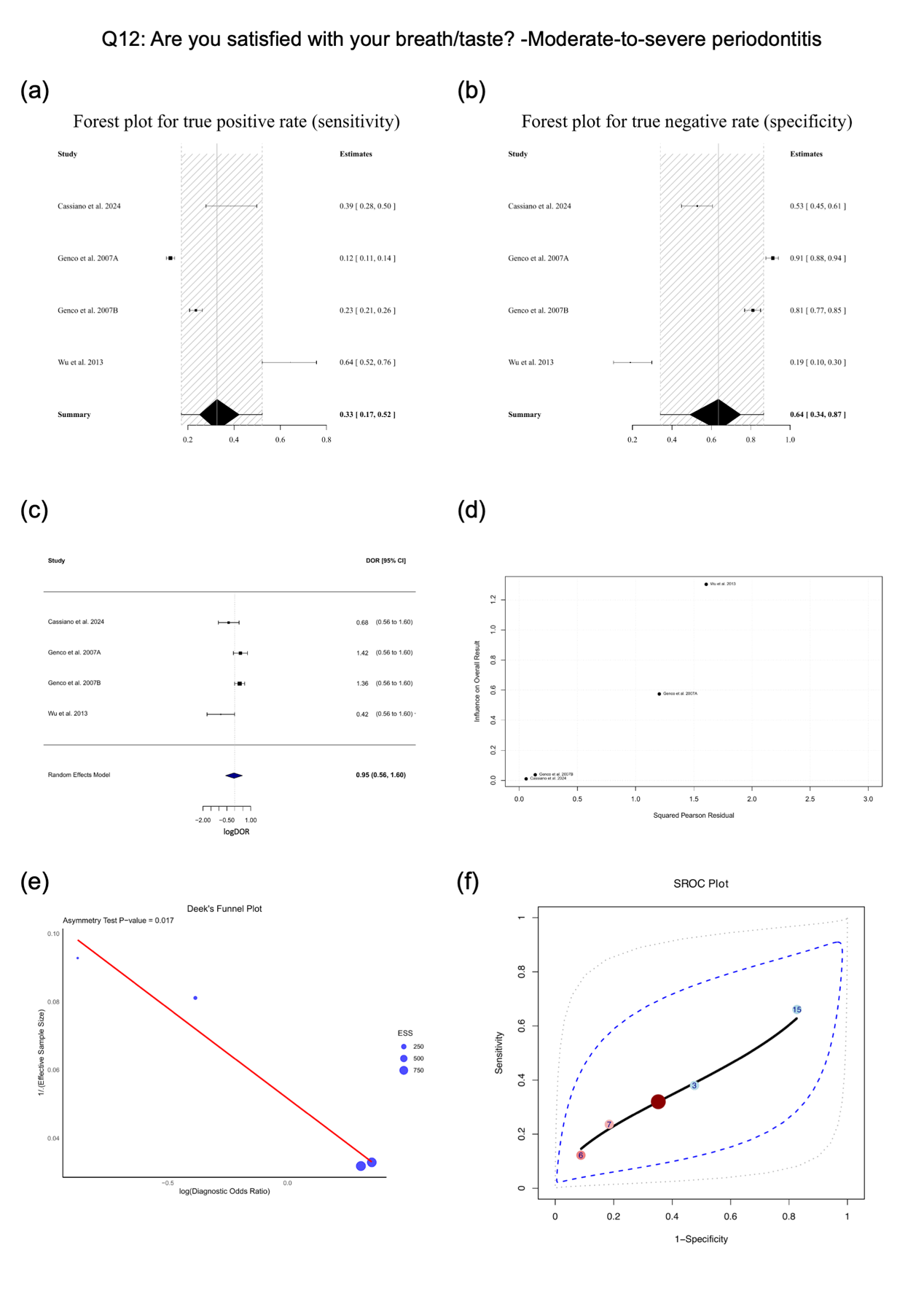
**

**Figure S33. Q12 "Are you satisfied with your breath/taste?" for detecting severe periodontitis: (a) Forest plot of sensitivity, (b) Forest plot of specificity, (c) Forest plot of diagnostic odds ratio (DOR), (d) Baujat plot, (e) Deeks's funnel plot, (f) HSROC curve.**

**
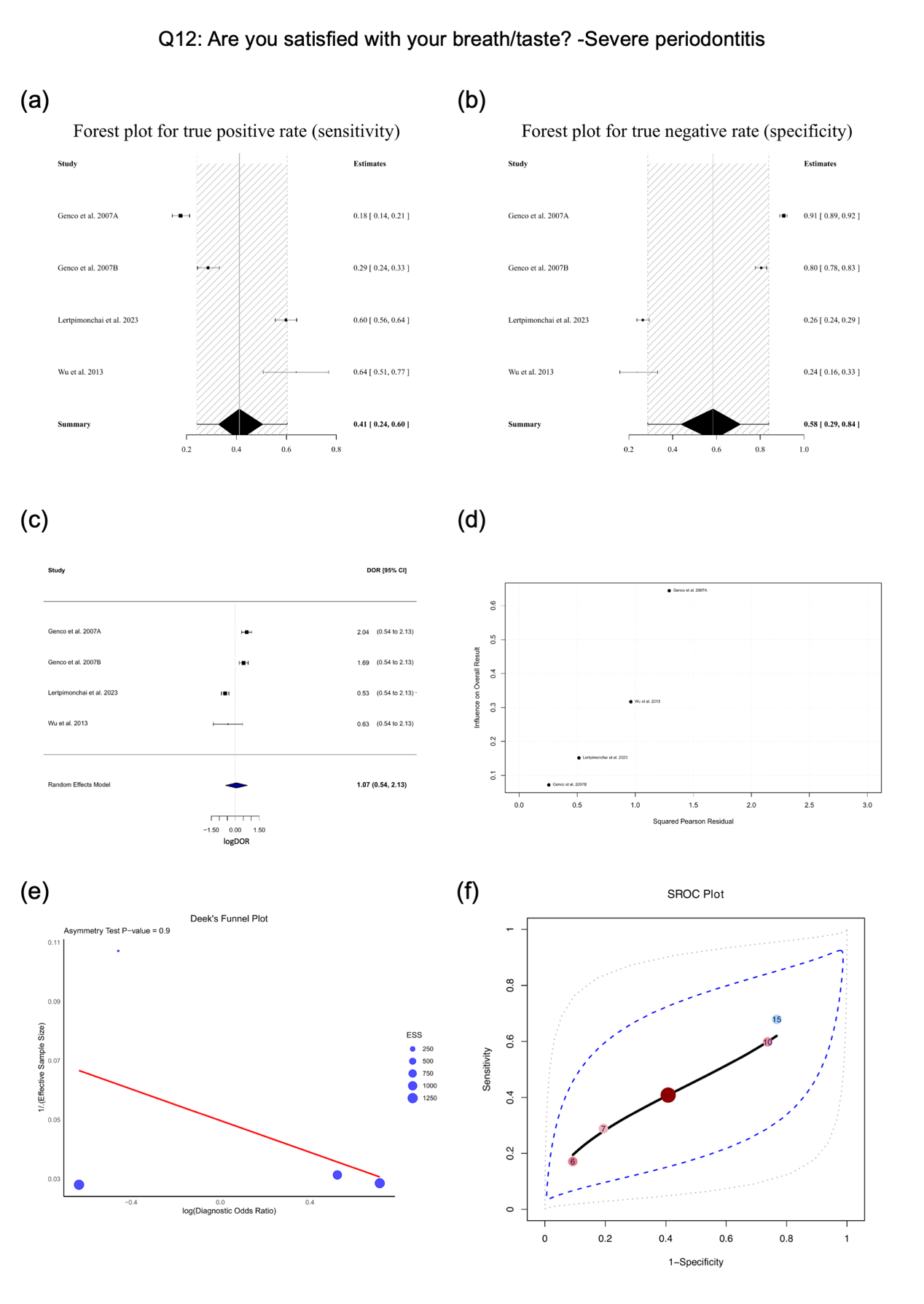
**

**Figure S34. Q13 "Are you satisfied with your breath/taste?" for detecting moderate-to-severe periodontitis: (a) Forest plot of sensitivity, (b) Forest plot of specificity, (c) Forest plot of diagnostic odds ratio (DOR), (d) Baujat plot, (e) Deeks's funnel plot, (f) HSROC curve.**

**
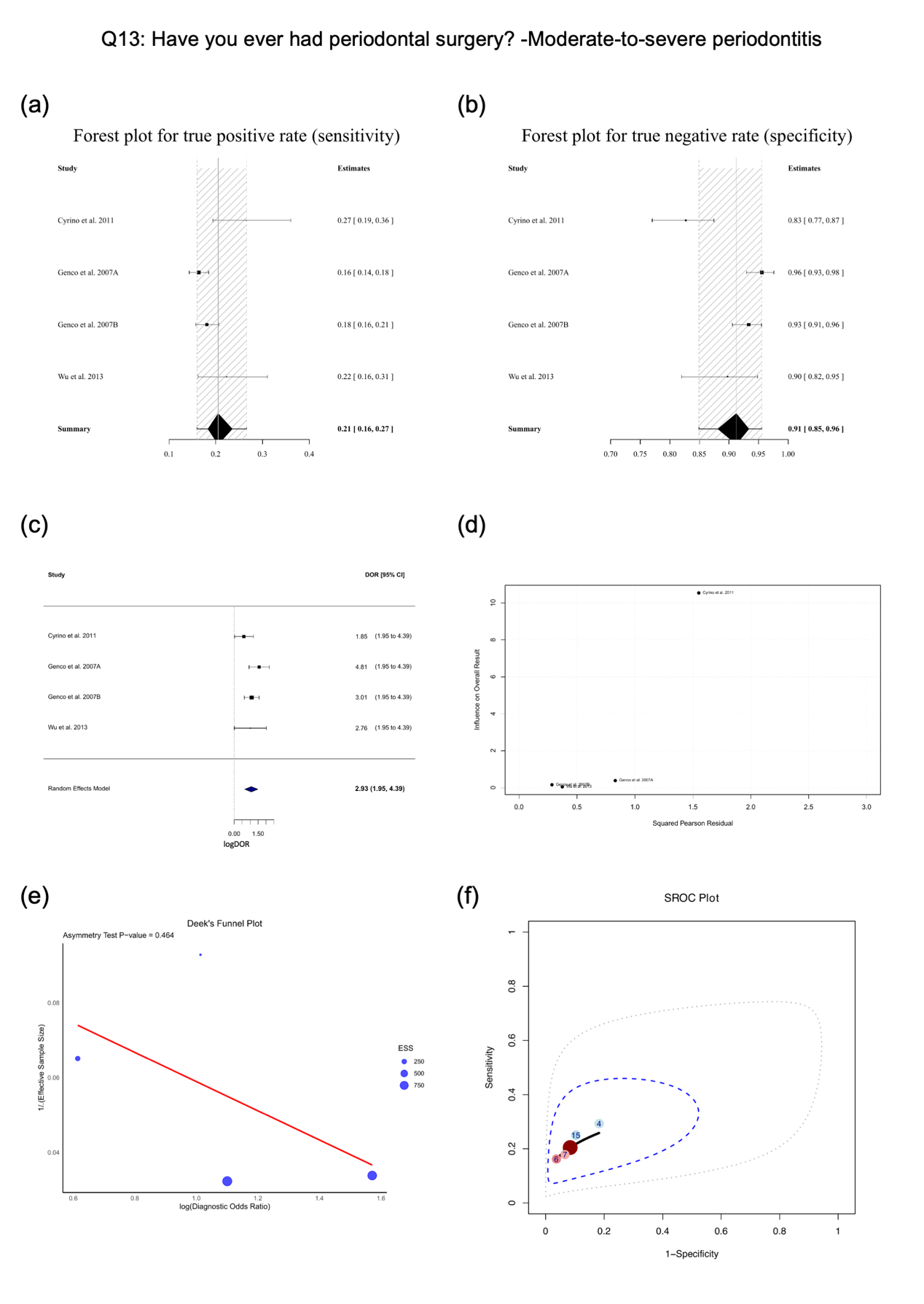
**

**Figure S35. Q13 "Are you satisfied with your breath/taste?" for detecting severe periodontitis: (a) Forest plot of sensitivity, (b) Forest plot of specificity, (c) Forest plot of diagnostic odds ratio (DOR), (d) Baujat plot, (e) Deeks's funnel plot, (f) HSROC curve.**

**
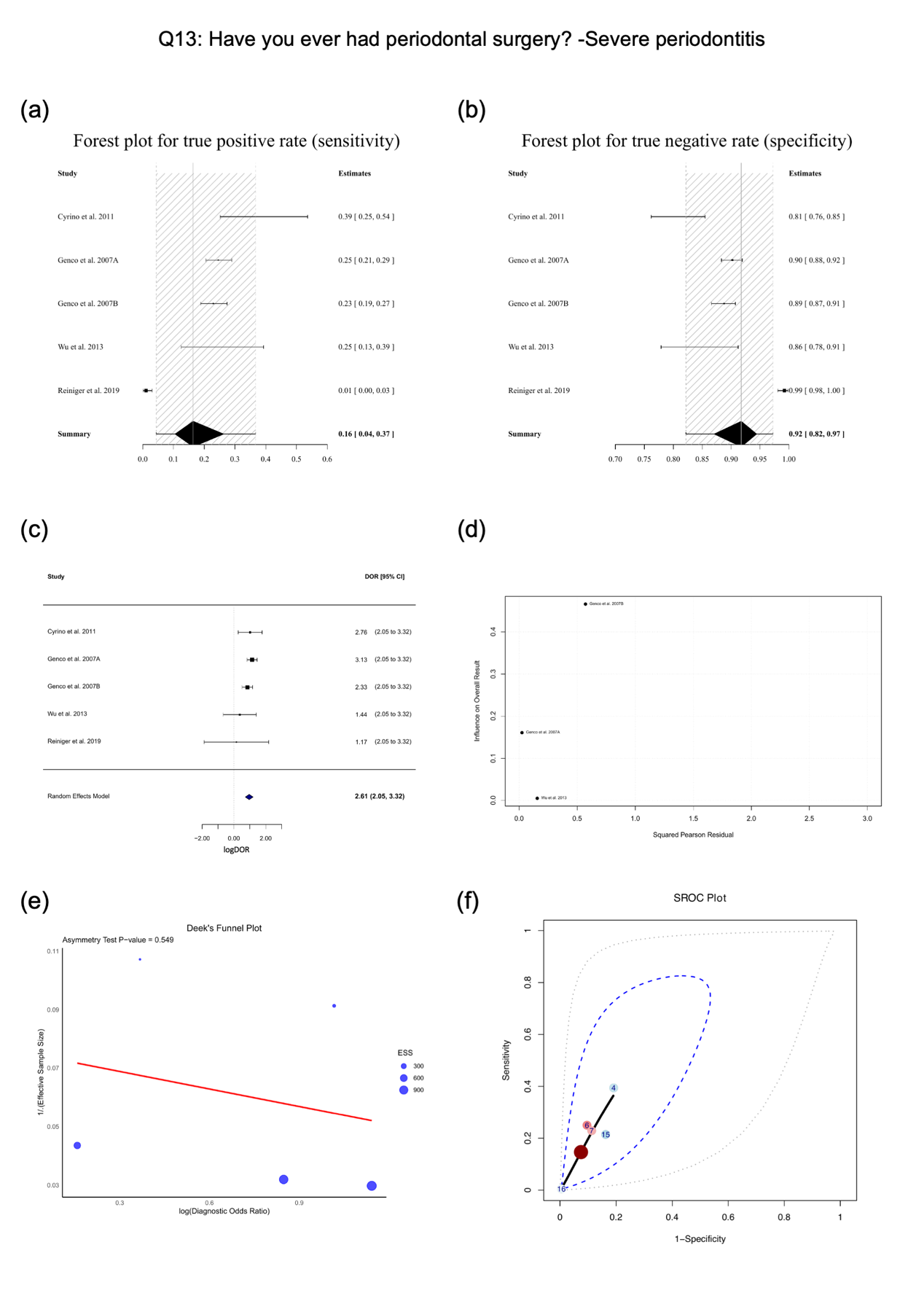
**

**Figure S36. Q14 "Frequency of tooth brushing" for detecting severe periodontitis: (a) Forest plot of sensitivity, (b) Forest plot of specificity, (c) Forest plot of diagnostic odds ratio (DOR), (d) Baujat plot, (e) Deeks's funnel plot, (f) HSROC curve.**

**
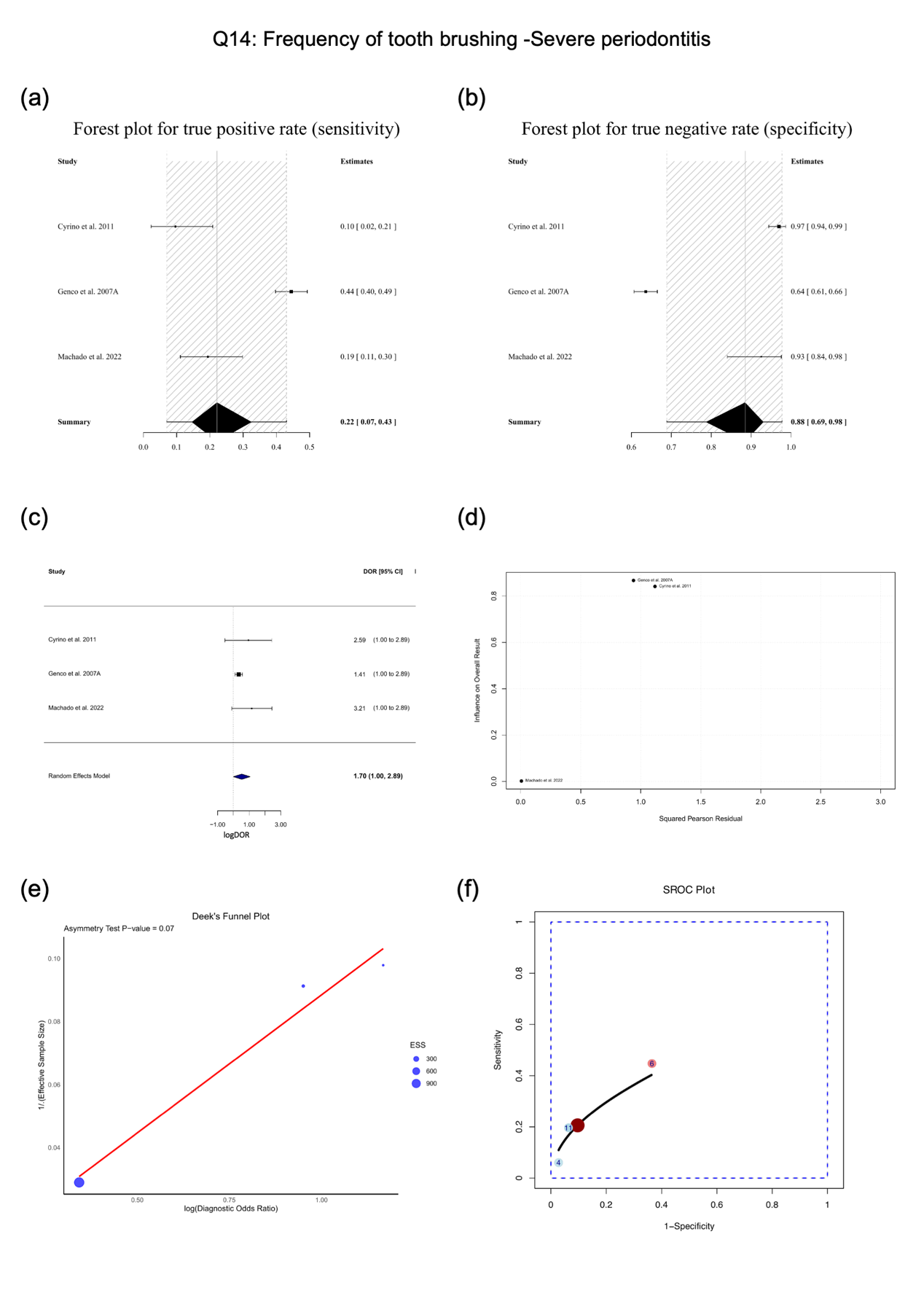
**

**Figure S37. Q15 "Frequency of Dental checkups" for detecting moderate-to-severe periodontitis: (a) Forest plot of sensitivity, (b) Forest plot of specificity, (c) Forest plot of diagnostic odds ratio (DOR), (d) Baujat plot, (e) Deeks's funnel plot, (f) HSROC curve.**

**
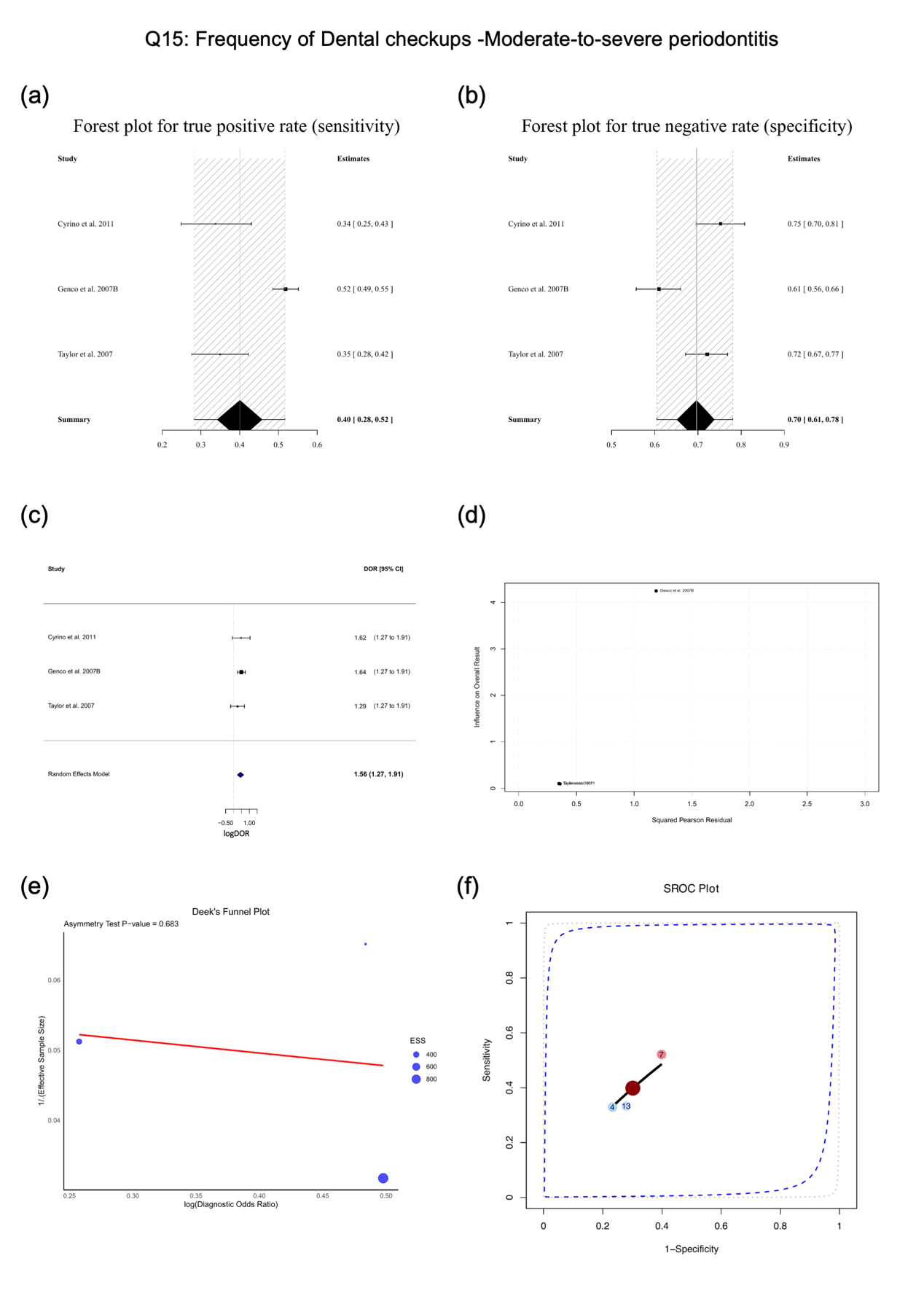
**

**Figure S38. Q15 "Frequency of Dental checkups" for detecting severe periodontitis: (a) Forest plot of sensitivity, (b) Forest plot of specificity, (c) Forest plot of diagnostic odds ratio (DOR), (d) Baujat plot, (e) Deeks's funnel plot, (f) HSROC curve.**

**
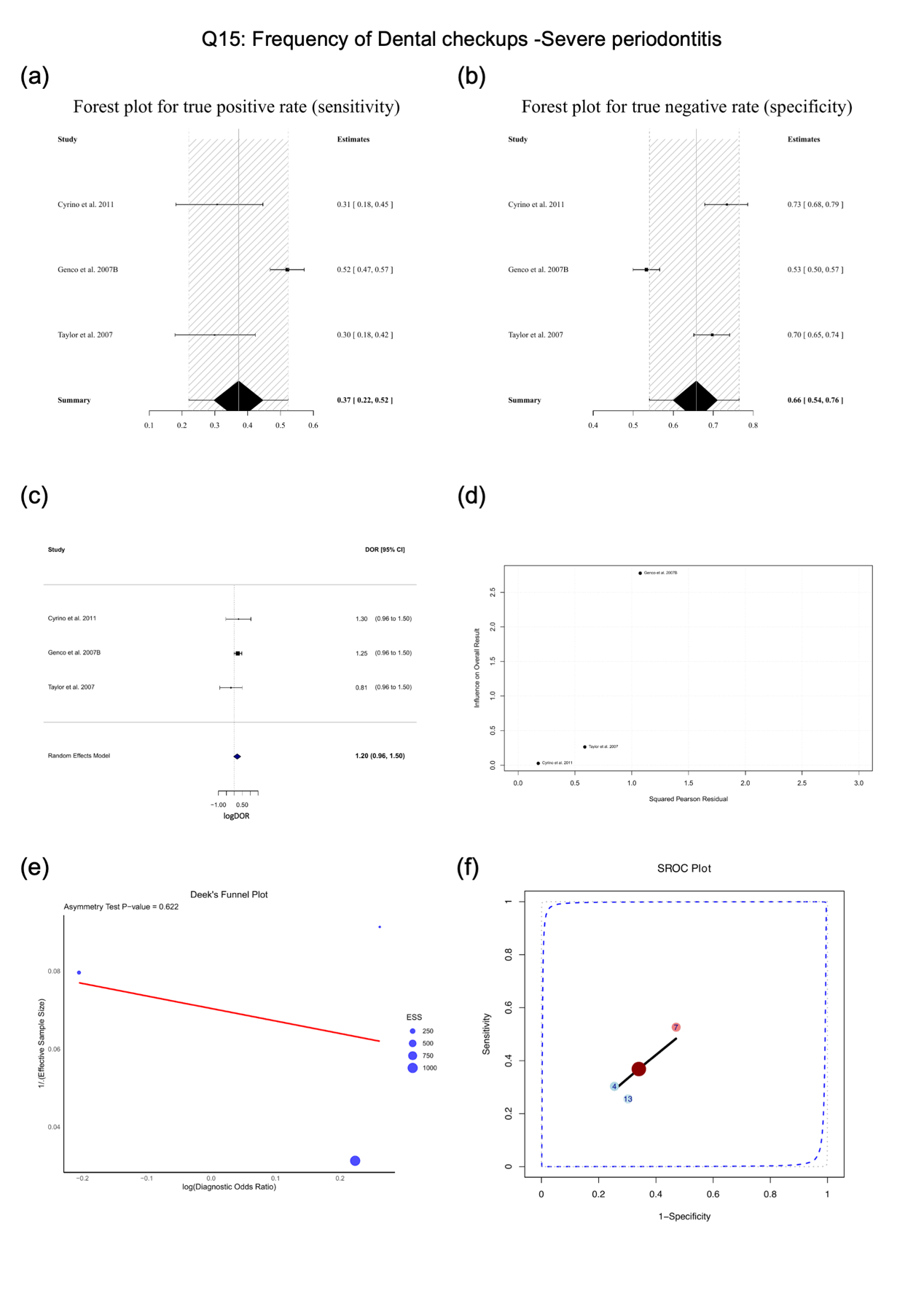
**

**Figure S39. Q16 "Do you have sore gums?" for detecting moderate-to-severe periodontitis: (a) Forest plot of sensitivity, (b) Forest plot of specificity, (c) Forest plot of diagnostic odds ratio (DOR), (d) Baujat plot, (e) Deeks's funnel plot, (f) HSROC curve.**

**
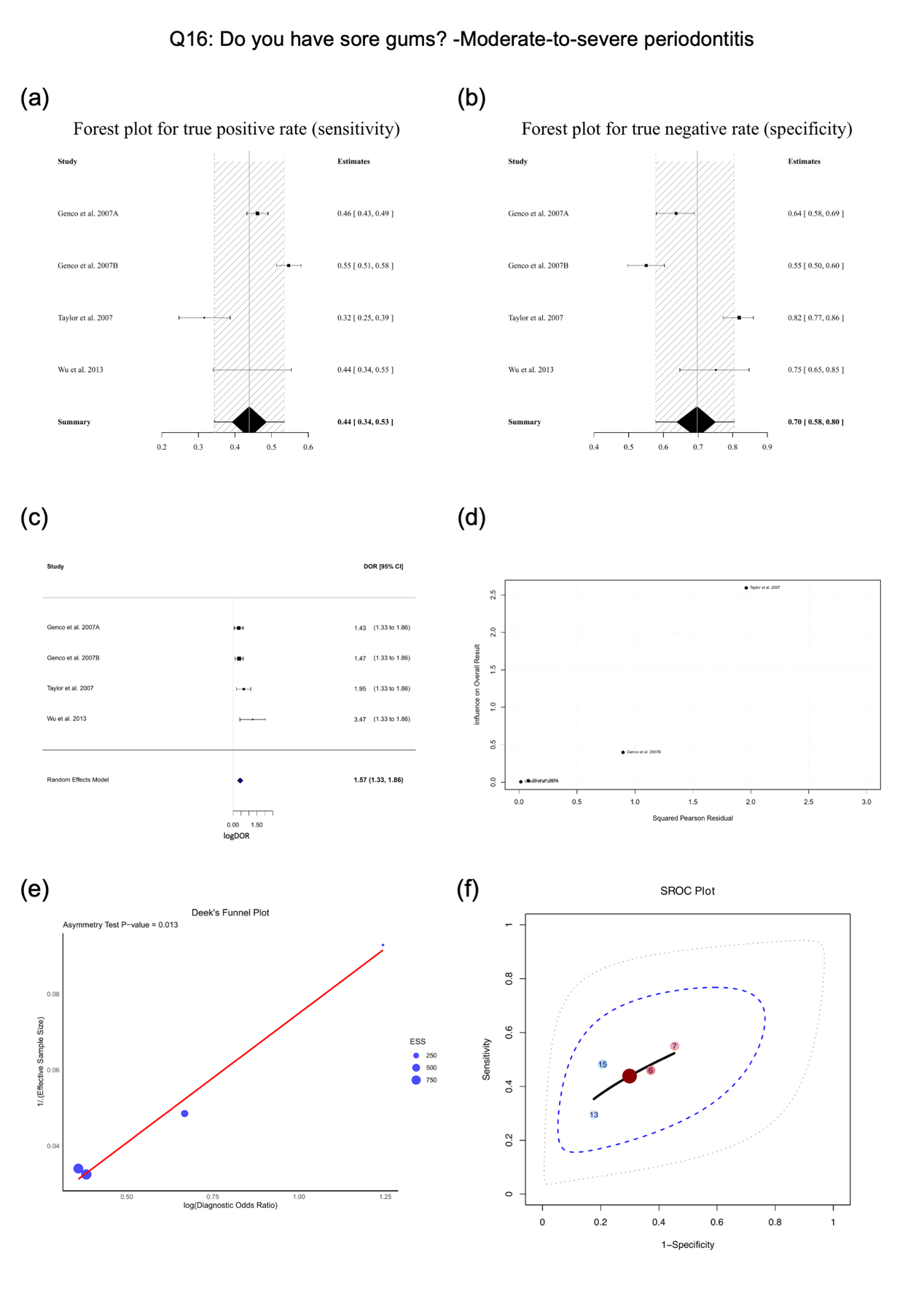
**

**Figure S40. Q16 "Do you have sore gums?" for detecting severe periodontitis: (a) Forest plot of sensitivity, (b) Forest plot of specificity, (c) Forest plot of diagnostic odds ratio (DOR), (d) Baujat plot, (e) Deeks's funnel plot, (f) HSROC curve.**

**
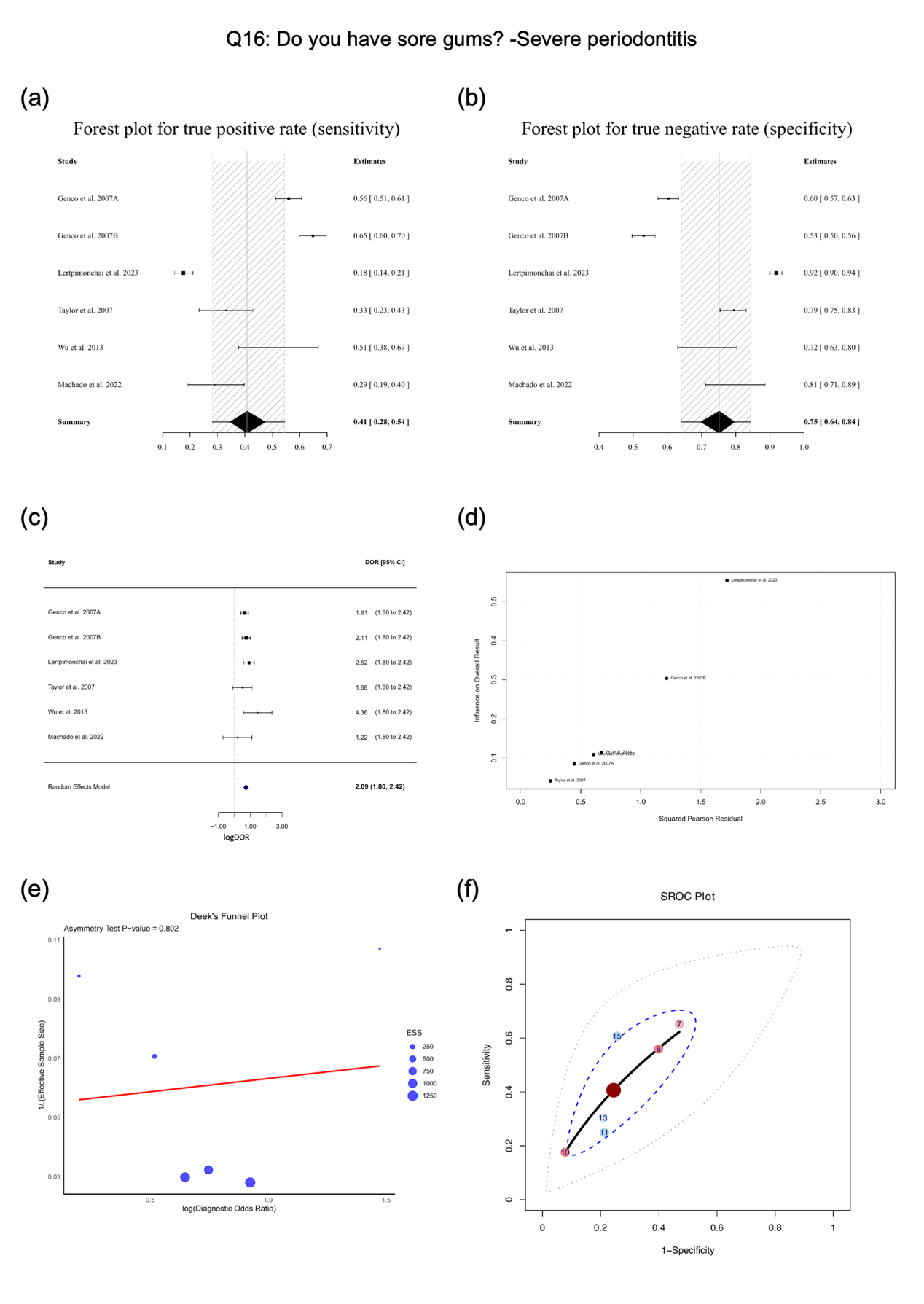
**

**Table S5. Leave-one-out analysis**

| **Questions** | **Clinical diagnose** | **Leave-out study** | **Diagnostic odds ratio (DOR)** | **95%CI lower** | **95%CI upper** |
| --- | --- | --- | --- | --- | --- |
| Q1: Do you think you might have gum disease? | Total periodontitis | Bond et al. 2024 | 7.22 | 1.79 | 29.11 |
|  |  | Cassiano et al. 2024 | 6.34 | 1.41 | 28.47 |
|  |  | Iwasaki et al. 2021 | 16.27 | 6.47 | 40.89 |
|  |  | Machado et al. 2022 | 6.77 | 1.42 | 32.32 |
|  | Moderate-to-severe periodontitis | Bond et al. 2024 | 4.06 | 2.59 | 6.35 |
|  |  | Carra et al. 2018 | 3.42 | 2.06 | 5.68 |
|  |  | Cassiano et al. 2024 | 2.87 | 1.91 | 4.31 |
|  |  | Cyrino et al. 2011 | 3.93 | 2.16 | 7.17 |
|  |  | Eke et al. 2009 | 3.53 | 1.96 | 6.34 |
|  |  | Heaton et al. 2017 | 3.56 | 2.11 | 6.01 |
|  |  | Iwasaki et al. 2021 | 4.04 | 2.23 | 7.31 |
|  |  | Montero et al. 2020 | 3.5 | 2.09 | 5.88 |
|  |  | Taylor et al. 2007 | 3.73 | 2.04 | 6.81 |
|  |  | Wu et al. 2013 | 3.68 | 2.04 | 6.64 |
|  | Severe periodontitis | Bond et al. 2024 | 3.47 | 2.65 | 4.54 |
|  |  | Carra et al. 2018 | 3.16 | 2.23 | 4.46 |
|  |  | Cyrino et al. 2011 | 3.23 | 2.27 | 4.6 |
|  |  | Eke et al. 2009 | 2.81 | 2.13 | 3.71 |
|  |  | Heaton et al. 2017 | 3.27 | 2.32 | 4.59 |
|  |  | Iwasaki et al. 2021 | 3.14 | 2.22 | 4.44 |
|  |  | Lertpimonchai et al. 2023 | 3.27 | 2.26 | 4.72 |
|  |  | Montero et al. 2020 | 3.26 | 2.29 | 4.64 |
|  |  | Reiniger et al. 2019 | 3.39 | 2.38 | 4.83 |
|  |  | Taylor et al. 2007 | 3.15 | 2.22 | 4.46 |
|  |  | Wu et al. 2013 | 3.29 | 2.32 | 4.66 |
|  |  | Machado et al. 2022 | 3.07 | 2.2 | 4.28 |
| Q2: Overall, how would you rate the health of your teeth and gums? | Total periodontitis | Carra et al. 2018 | 3 | 1.42 | 6.37 |
|  |  | Cassiano et al. 2024 | 2.66 | 1.21 | 5.84 |
|  |  | Iwasaki et al. 2021 | 3.71 | 2.31 | 5.96 |
|  |  | Machado et al. 2022 | 2.29 | 1.34 | 3.94 |
|  | Moderate-to-severe periodontitis | Bond et al. 2024 | 2.77 | 1.89 | 4.05 |
|  |  | Carra et al. 2018 | 2.91 | 2.06 | 4.12 |
|  |  | Cassiano et al. 2024 | 2.62 | 1.95 | 3.53 |
|  |  | Cyrino et al. 2011 | 2.88 | 2.01 | 4.13 |
|  |  | Eke et al. 2009 | 2.8 | 1.94 | 4.05 |
|  |  | Heaton et al. 2017 | 2.85 | 2.07 | 3.94 |
|  |  | Iwasaki et al. 2021 | 3.18 | 2.47 | 4.1 |
|  |  | Montero et al. 2020 | 2.85 | 2.03 | 4.01 |
|  |  | Taylor et al. 2007 | 3.07 | 2.16 | 4.36 |
|  |  | Wu et al. 2013 | 2.81 | 2.02 | 3.91 |
|  | Severe periodontitis | Bond et al. 2024 | 2.63 | 2.05 | 3.37 |
|  |  | Carra et al. 2018 | 2.81 | 2.16 | 3.65 |
|  |  | Cyrino et al. 2011 | 2.88 | 2.26 | 3.67 |
|  |  | Eke et al. 2009 | 2.74 | 2.16 | 3.46 |
|  |  | Heaton et al. 2017 | 2.78 | 2.2 | 3.51 |
|  |  | Iwasaki et al. 2021 | 2.87 | 2.27 | 3.63 |
|  |  | Montero et al. 2020 | 3.07 | 2.53 | 3.73 |
|  |  | Taylor et al. 2007 | 2.97 | 2.35 | 3.75 |
|  |  | Wu et al. 2013 | 2.88 | 2.29 | 3.63 |
|  |  | Machado et al. 2022 | 2.72 | 2.16 | 3.44 |
|  |  | Reiniger et al. 2019 | 2.86 | 2.16 | 3.79 |
| Q3: Have you ever had treatment for gum disease such as scaling and root planing, sometimes called “deep cleaning”? | Total periodontitis | Carra et al. 2018 | 4.19 | 0.83 | 21.12 |
|  |  | Cassiano et al. 2024 | 2.64 | 0.53 | 13.2 |
|  |  | Iwasaki et al. 2021 | 5.39 | 1.67 | 17.43 |
|  |  | Machado et al. 2022 | 2.16 | 0.68 | 6.86 |
|  | Moderate-to-severe periodontitis | Bond et al. 2024 | 1.86 | 1.15 | 3.01 |
|  |  | Carra et al. 2018 | 1.97 | 1.25 | 3.08 |
|  |  | Cassiano et al. 2024 | 1.69 | 1.22 | 2.33 |
|  |  | Cyrino et al. 2011 | 1.92 | 1.19 | 3.09 |
|  |  | Eke et al. 2009 | 2 | 1.24 | 3.24 |
|  |  | Genco et al. 2007A | 1.9 | 1.17 | 3.1 |
|  |  | Heaton et al. 2017 | 1.86 | 1.2 | 2.87 |
|  |  | Iwasaki et al. 2021 | 2.11 | 1.34 | 3.31 |
|  |  | Montero et al. 2020 | 2.07 | 1.33 | 3.23 |
|  |  | Wu et al. 2013 | 2.14 | 1.42 | 3.22 |
|  | Severe periodontitis | Bond et al. 2024 | 2.24 | 1.53 | 3.28 |
|  |  | Carra et al. 2018 | 2.27 | 1.57 | 3.29 |
|  |  | Cyrino et al. 2011 | 2.31 | 1.59 | 3.35 |
|  |  | Eke et al. 2009 | 2.4 | 1.65 | 3.49 |
|  |  | Genco et al. 2007A | 2.34 | 1.57 | 3.49 |
|  |  | Heaton et al. 2017 | 2.26 | 1.59 | 3.21 |
|  |  | Iwasaki et al. 2021 | 2.4 | 1.63 | 3.53 |
|  |  | Montero et al. 2020 | 2.61 | 1.98 | 3.43 |
|  |  | Wu et al. 2013 | 2.56 | 1.87 | 3.49 |
|  |  | Machado et al. 2022 | 2.19 | 1.58 | 3.02 |
|  |  | Reiniger et al. 2019 | 2.42 | 1.64 | 3.58 |
| Q4: Have you ever had any teeth become loose on their own, without an injury? | Total periodontitis | Carra et al. 2018 | 2.7 | 1.33 | 5.48 |
|  |  | Iwasaki et al. 2021 | 2.31 | 0.94 | 5.7 |
|  |  | Ueno et al. 2020 | 2.94 | 1.53 | 5.66 |
|  |  | Machado et al. 2022 | 1.99 | 1.28 | 3.1 |
|  | Moderate-to-severe periodontitis | Bond et al. 2024 | 3.36 | 2.04 | 5.55 |
|  |  | Carra et al. 2018 | 3.65 | 2.35 | 5.67 |
|  |  | Cyrino et al. 2011 | 3.33 | 2.08 | 5.35 |
|  |  | Eke et al. 2009 | 3.26 | 2.02 | 5.26 |
|  |  | Heaton et al. 2017 | 3.53 | 2.27 | 5.49 |
|  |  | Iwasaki et al. 2021 | 3.56 | 2.18 | 5.8 |
|  |  | Montero et al. 2020 | 3.42 | 2.16 | 5.41 |
|  |  | Taylor et al. 2007 | 3.26 | 2.03 | 5.23 |
|  |  | Ueno et al. 2020 | 3.91 | 2.77 | 5.52 |
|  |  | Wu et al. 2013 | 2.96 | 2.07 | 4.22 |
|  | Severe periodontitis | Bond et al. 2024 | 5.63 | 3.66 | 8.66 |
|  |  | Carra et al. 2018 | 5.6 | 3.67 | 8.54 |
|  |  | Cyrino et al. 2011 | 5.52 | 3.64 | 8.36 |
|  |  | Eke et al. 2009 | 5.14 | 3.49 | 7.55 |
|  |  | Heaton et al. 2017 | 5.77 | 3.91 | 8.5 |
|  |  | Iwasaki et al. 2021 | 5.19 | 3.47 | 7.76 |
|  |  | Lertpimonchai et al. 2023 | 5.47 | 3.56 | 8.4 |
|  |  | Montero et al. 2020 | 5.97 | 4.38 | 8.15 |
|  |  | Taylor et al. 2007 | 5.17 | 3.48 | 7.69 |
|  |  | Ueno et al. 2020 | 5.83 | 3.89 | 8.75 |
|  |  | Wu et al. 2013 | 4.98 | 3.52 | 7.04 |
|  |  | Machado et al. 2022 | 5.38 | 3.57 | 8.1 |
|  |  | Reiniger et al. 2019 | 5.49 | 3.57 | 8.43 |
| Q5: Have you ever been told by a dental professional that you lost bone around your teeth? | Total periodontitis | Carra et al. 2018 | 4.07 | 0.44 | 37.28 |
|  |  | Cassiano et al. 2024 | 6.38 | 0.53 | 76.71 |
|  |  | Iwasaki et al. 2021 | 7.94 | 0.93 | 68.03 |
|  |  | Machado et al. 2022 | 1.33 | 0.84 | 2.11 |
|  | Moderate-to-severe periodontitis | Bond et al. 2024 | 2.58 | 1.6 | 4.16 |
|  |  | Carra et al. 2018 | 2.45 | 1.71 | 3.5 |
|  |  | Cassiano et al. 2024 | 2.44 | 1.66 | 3.58 |
|  |  | Cyrino et al. 2011 | 2.6 | 1.7 | 3.96 |
|  |  | Eke et al. 2009 | 2.2 | 1.93 | 2.51 |
|  |  | Heaton et al. 2017 | 2.42 | 1.7 | 3.45 |
|  |  | Iwasaki et al. 2021 | 2.34 | 1.52 | 3.62 |
|  |  | Montero et al. 2020 | 2.69 | 1.96 | 3.71 |
|  |  | Wu et al. 2013 | 2.61 | 1.79 | 3.79 |
|  | Severe periodontitis | Bond et al. 2024 | 4.02 | 2.25 | 7.18 |
|  |  | Carra et al. 2018 | 3.46 | 2 | 5.99 |
|  |  | Cyrino et al. 2011 | 3.8 | 2.12 | 6.8 |
|  |  | Eke et al. 2009 | 3.72 | 2.09 | 6.62 |
|  |  | Heaton et al. 2017 | 3.89 | 2.21 | 6.87 |
|  |  | Iwasaki et al. 2021 | 3.33 | 1.96 | 5.65 |
|  |  | Montero et al. 2020 | 4.4 | 2.85 | 6.78 |
|  |  | Wu et al. 2013 | 3.86 | 2.19 | 6.82 |
|  |  | Machado et al. 2022 | 3.25 | 1.99 | 5.29 |
|  |  | Reiniger et al. 2019 | 3.76 | 2.09 | 6.75 |
| Q6: During the past 3 months, have you noticed a tooth that doesn’t look right? | Total periodontitis | Carra et al. 2018 | 6.46 | 0.58 | 72.21 |
|  |  | Iwasaki et al. 2021 | 5.23 | 0.28 | 96.85 |
|  |  | Machado et al. 2022 | 1.86 | 1.33 | 2.61 |
|  | Moderate-to-severe periodontitis | Bond et al. 2024 | 2 | 1.6 | 2.5 |
|  |  | Carra et al. 2018 | 2.29 | 1.97 | 2.66 |
|  |  | Eke et al. 2009 | 2.17 | 1.77 | 2.66 |
|  |  | Heaton et al. 2017 | 2.23 | 1.89 | 2.63 |
|  |  | Iwasaki et al. 2021 | 2.39 | 2.13 | 2.69 |
|  |  | Montero et al. 2020 | 2.21 | 1.87 | 2.62 |
|  |  | Taylor et al. 2007 | 2.2 | 1.79 | 2.72 |
|  | Severe periodontitis | Bond et al. 2024 | 2.7 | 1.76 | 4.15 |
|  |  | Carra et al. 2018 | 2.78 | 1.87 | 4.12 |
|  |  | Eke et al. 2009 | 2.36 | 1.85 | 3.01 |
|  |  | Heaton et al. 2017 | 2.67 | 1.88 | 3.81 |
|  |  | Iwasaki et al. 2021 | 2.7 | 1.8 | 4.05 |
|  |  | Montero et al. 2020 | 2.8 | 1.93 | 4.05 |
|  |  | Taylor et al. 2007 | 2.7 | 1.79 | 4.05 |
|  |  | Machado et al. 2022 | 2.31 | 1.93 | 2.77 |
|  |  | Reiniger et al. 2019 | 2.83 | 1.93 | 4.14 |
| Q7: Do you use Dental Floss/other device to clean your teeth? | Total periodontitis | Carra et al. 2018 | 1.08 | 0.68 | 1.71 |
|  |  | Cassiano et al. 2024 | 1.34 | 1.14 | 1.59 |
|  |  | Iwasaki et al. 2021 | 1.04 | 0.59 | 1.84 |
|  |  | Ueno et al. 2020 | 0.97 | 0.59 | 1.59 |
|  |  | Machado et al. 2022 | 1.05 | 0.65 | 1.7 |
|  | Moderate-to-severe periodontitis | Carra et al. 2018 | 1.29 | 0.94 | 1.75 |
|  |  | Cassiano et al. 2024 | 1.35 | 1.01 | 1.8 |
|  |  | Cyrino et al. 2011 | 1.19 | 0.91 | 1.54 |
|  |  | Eke et al. 2009 | 1.21 | 0.9 | 1.61 |
|  |  | Genco et al. 2007A | 1.3 | 0.93 | 1.81 |
|  |  | Genco et al. 2007B | 1.31 | 0.94 | 1.82 |
|  |  | Heaton et al. 2017 | 1.28 | 0.96 | 1.71 |
|  |  | Iwasaki et al. 2021 | 1.31 | 0.95 | 1.82 |
|  |  | Montero et al. 2020 | 1.25 | 0.92 | 1.7 |
|  |  | Taylor et al. 2007 | 1.38 | 1.09 | 1.76 |
|  |  | Ueno et al. 2020 | 1.26 | 0.9 | 1.76 |
|  |  | Wu et al. 2013 | 1.24 | 0.91 | 1.67 |
|  | Severe periodontitis | Carra et al. 2018 | 1.23 | 0.84 | 1.8 |
|  |  | Cyrino et al. 2011 | 1.1 | 0.76 | 1.59 |
|  |  | Eke et al. 2009 | 1.08 | 0.76 | 1.53 |
|  |  | Genco et al. 2007A | 1.17 | 0.78 | 1.76 |
|  |  | Genco et al. 2007B | 1.19 | 0.79 | 1.79 |
|  |  | Heaton et al. 2017 | 1.18 | 0.82 | 1.71 |
|  |  | Iwasaki et al. 2021 | 1.19 | 0.8 | 1.78 |
|  |  | Montero et al. 2020 | 1.11 | 0.76 | 1.63 |
|  |  | Taylor et al. 2007 | 1.28 | 0.99 | 1.66 |
|  |  | Ueno et al. 2020 | 1.19 | 0.79 | 1.79 |
|  |  | Wu et al. 2013 | 1.12 | 0.77 | 1.64 |
|  |  | Machado et al. 2022 | 1.16 | 0.78 | 1.72 |
|  |  | Reiniger et al. 2019 | 1.12 | 0.76 | 1.65 |
| Q8: Do you use mouthwash or other dental rinse product to treat dental disease or dental problems? | Total periodontitis | Carra et al. 2018 | 0.93 | 0.7 | 1.23 |
|  |  | Cassiano et al. 2024 | 0.93 | 0.7 | 1.23 |
|  |  | Iwasaki et al. 2021 | 0.88 | 0.45 | 1.72 |
|  | Moderate-to-severe periodontitis | Carra et al. 2018 | 0.85 | 0.63 | 1.14 |
|  |  | Cassiano et al. 2024 | 0.88 | 0.66 | 1.18 |
|  |  | Eke et al. 2009 | 0.86 | 0.61 | 1.2 |
|  |  | Heaton et al. 2017 | 0.87 | 0.65 | 1.15 |
|  |  | Iwasaki et al. 2021 | 0.82 | 0.58 | 1.15 |
|  |  | Montero et al. 2020 | 0.8 | 0.62 | 1.04 |
|  |  | Taylor et al. 2007 | 0.96 | 0.77 | 1.19 |
|  |  | Wu et al. 2013 | 0.82 | 0.61 | 1.09 |
|  | Severe periodontitis | Carra et al. 2018 | 0.81 | 0.56 | 1.16 |
|  |  | Eke et al. 2009 | 0.72 | 0.5 | 1.04 |
|  |  | Heaton et al. 2017 | 0.76 | 0.53 | 1.08 |
|  |  | Iwasaki et al. 2021 | 0.68 | 0.49 | 0.94 |
|  |  | Montero et al. 2020 | 0.7 | 0.48 | 1.01 |
|  |  | Taylor et al. 2007 | 0.83 | 0.61 | 1.13 |
|  |  | Wu et al. 2013 | 0.74 | 0.51 | 1.07 |
| Q9: Do you have bleeding gums? | Total periodontitis | Carra et al. 2018 | 1.55 | 1.32 | 1.83 |
|  |  | Iwasaki et al. 2021 | 2.35 | 0.95 | 5.77 |
|  |  | Ueno et al. 2020 | 2.33 | 1.06 | 5.12 |
|  |  | Machado et al. 2022 | 1.57 | 1.33 | 1.84 |
|  | Moderate-to-severe periodontitis | Carra et al. 2018 | 1.48 | 1.31 | 1.66 |
|  |  | Genco et al. 2007A | 1.49 | 1.31 | 1.7 |
|  |  | Genco et al. 2007B | 1.46 | 1.28 | 1.67 |
|  |  | Iwasaki et al. 2021 | 1.45 | 1.28 | 1.66 |
|  |  | Taylor et al. 2007 | 1.52 | 1.34 | 1.71 |
|  |  | Ueno et al. 2020 | 1.55 | 1.35 | 1.79 |
|  |  | Wu et al. 2013 | 1.49 | 1.33 | 1.68 |
|  | Severe periodontitis | Carra et al. 2018 | 2.01 | 1.57 | 2.59 |
|  |  | Genco et al. 2007A | 1.85 | 1.43 | 2.39 |
|  |  | Genco et al. 2007B | 2.01 | 1.5 | 2.68 |
|  |  | Iwasaki et al. 2021 | 1.8 | 1.48 | 2.18 |
|  |  | Taylor et al. 2007 | 1.99 | 1.56 | 2.55 |
|  |  | Ueno et al. 2020 | 2.06 | 1.63 | 2.6 |
|  |  | Wu et al. 2013 | 1.88 | 1.51 | 2.34 |
|  |  | Machado et al. 2022 | 1.89 | 1.51 | 2.36 |
| Q10: Do you have tooth loss? | Moderate-to-severe periodontitis | Cassiano et al. 2024 | 2.63 | 1.4 | 4.96 |
|  |  | Genco et al. 2007A | 3.09 | 1.42 | 6.72 |
|  |  | Genco et al. 2007B | 3.76 | 2.6 | 5.43 |
|  |  | Taylor et al. 2007 | 2.59 | 1.36 | 4.94 |
|  | Severe periodontitis | Genco et al. 2007A | 2.52 | 0.64 | 9.84 |
|  |  | Genco et al. 2007B | 2.92 | 1.03 | 8.3 |
|  |  | Taylor et al. 2007 | 1.53 | 1.12 | 2.1 |
| Q11: Do you have tooth loss because of mobility? | Severe periodontitis | Cyrino et al. 2011 | 8.49 | 3.74 | 19.27 |
|  |  | Wu et al. 2013 | 5.16 | 3.53 | 7.53 |
|  |  | Machado et al. 2022 | 6.58 | 3.18 | 13.61 |
|  |  | Reiniger et al. 2019 | 8.6 | 3.51 | 21.06 |
| Q12: Are you satisfied with your breath/taste? | Moderate-to-severe periodontitis | Cassiano et al. 2024 | 1.03 | 0.53 | 2 |
|  |  | Genco et al. 2007A | 0.8 | 0.4 | 1.58 |
|  |  | Genco et al. 2007B | 0.79 | 0.39 | 1.61 |
|  |  | Wu et al. 2013 | 1.19 | 0.84 | 1.7 |
|  | Severe periodontitis | Genco et al. 2007A | 0.85 | 0.39 | 1.84 |
|  |  | Genco et al. 2007B | 0.9 | 0.37 | 2.19 |
|  |  | Lertpimonchai et al. 2023 | 1.52 | 0.92 | 2.51 |
|  |  | Wu et al. 2013 | 1.22 | 0.53 | 2.78 |
| Q13: Have you ever had periodontal surgery? | Moderate-to-severe periodontitis | Cyrino et al. 2011 | 3.43 | 2.42 | 4.87 |
|  |  | Genco et al. 2007A | 2.51 | 1.74 | 3.62 |
|  |  | Genco et al. 2007B | 2.9 | 1.55 | 5.43 |
|  |  | Wu et al. 2013 | 2.96 | 1.79 | 4.89 |
|  | Severe periodontitis | Cyrino et al. 2011 | 2.55 | 1.91 | 3.41 |
|  |  | Genco et al. 2007A | 2.27 | 1.71 | 3.03 |
|  |  | Genco et al. 2007B | 2.86 | 2.16 | 3.78 |
|  |  | Wu et al. 2013 | 2.7 | 2.12 | 3.42 |
|  |  | Reiniger et al. 2019 | 2.64 | 2.08 | 3.35 |
| Q14: Frequency of tooth brushing | Severe periodontitis | Cyrino et al. 2011 | 1.67 | 0.87 | 3.2 |
|  |  | Genco et al. 2007A | 2.93 | 1.12 | 7.67 |
|  |  | Machado et al. 2022 | 1.43 | 1.14 | 1.79 |
| Q15: Frequency of Dental checkups | Moderate-to-severe periodontitis | Cyrino et al. 2011 | 1.55 | 1.24 | 1.93 |
|  |  | Genco et al. 2007B | 1.41 | 1 | 1.98 |
|  |  | Taylor et al. 2007 | 1.64 | 1.3 | 2.07 |
|  | Severe periodontitis | Cyrino et al. 2011 | 1.15 | 0.83 | 1.6 |
|  |  | Genco et al. 2007B | 1.01 | 0.6 | 1.7 |
|  |  | Taylor et al. 2007 | 1.25 | 0.99 | 1.59 |
| Q16: Do you have sore gums? | Moderate-to-severe periodontitis | Genco et al. 2007A | 1.88 | 1.26 | 2.8 |
|  |  | Genco et al. 2007B | 1.88 | 1.23 | 2.86 |
|  |  | Taylor et al. 2007 | 1.52 | 1.26 | 1.82 |
|  |  | Wu et al. 2013 | 1.52 | 1.28 | 1.8 |
|  | Severe periodontitis | Genco et al. 2007A | 2.2 | 1.83 | 2.66 |
|  |  | Genco et al. 2007B | 2.09 | 1.68 | 2.61 |
|  |  | Lertpimonchai et al. 2023 | 2 | 1.69 | 2.35 |
|  |  | Taylor et al. 2007 | 2.11 | 1.82 | 2.46 |
|  |  | Wu et al. 2013 | 2.04 | 1.76 | 2.37 |
|  |  | Machado et al. 2022 | 2.12 | 1.82 | 2.46 |
